# Quantitative genetic parameters for yield, plant growth and cone chemical traits in hop (*Humulus lupulus* L.)

**DOI:** 10.1186/1471-2156-15-22

**Published:** 2014-02-13

**Authors:** Erin L McAdam, René E Vaillancourt, Anthony Koutoulis, Simon P Whittock

**Affiliations:** 1 School of Biological Sciences, University of Tasmania, Private Bag 55 Hobart, TAS 7001, Australia; 2 Hop Products Australia, 26 Cambridge Road, Bellerive, TAS 7018, Australia

**Keywords:** *Humulus lupulus* L, Narrow-sense heritability, Genetic correlation, Hop acid, Dioecy, Quantitative genetics

## Abstract

**Background:**

Most traits targeted in the genetic improvement of hop are quantitative in nature. Improvement based on selection of these traits requires a comprehensive understanding of their inheritance. This study estimated quantitative genetic parameters for 20 traits related to three key objectives for the genetic improvement of hop: cone chemistry, cone yield and agronomic characteristics.

**Results:**

Significant heritable genetic variation was identified for α-acid and β-acid, as well as their components and relative proportions. Estimates of narrow-sense heritability for these traits (*h*^*2*^ = 0.15 to 0.29) were lower than those reported in previous hop studies, but were based on a broader suite of families (108 from European, North American and hybrid origins). Narrow-sense heritabilities are reported for hop growth traits for the first time (*h*^*2*^ = 0.04 to 0.20), relating to important agronomic characteristics such as emergence, height and lateral morphology. Cone chemistry and growth traits were significantly genetically correlated, such that families with more vigorous vegetative growth were associated with lower α-acid and β-acid levels. This trend may reflect the underlying population structure of founder genotypes (European and North American origins) as well as past selection in the Australian environment. Although male and female hop plants are thought to be indistinguishable until flowering, sex was found to influence variation in many growth traits, with male and female plants displaying differences in vegetative morphology from emergence to cone maturity.

**Conclusions:**

This study reveals important insights into the genetic control of quantitative hop traits. The information gained will provide hop breeders with a greater understanding of the additive genetic factors which affect selection of cone chemistry, yield and agronomic characteristics in hop, aiding in the future development of improved cultivars.

## Background

In the development of new crop cultivars, breeders are confronted with choosing among many potential selection criteria. In hop (*Humulus lupulus* L.) these criteria include yield per hectare, agronomic suitability (which is based on morphological characteristics of the plant) and brewing quality (which is primarily based on the chemical characteristics of the cone). Making genetic improvements to these criteria is complex as many of the traits relevant to them are quantitative characters, likely controlled by a large number of genes, each with small effects. Quantitative genetics is the study of the effect that genetics and the environment have on phenotypic variation, and provides extensive information on the inheritance of traits. Such information includes the amount of heritable genetic variation in traits available for selection, genetic correlations between traits as well as the degree to which genetic variation and correlations are influenced by environmental factors [1-3]. In hop, which is dioecious [4,5], quantitative genetic analysis of progeny trials has the added benefit of providing a means of assessing the genetic potential of male plants for traits expressed only in female plants. These traits include those relating to the yield and the quality of the commercially important hop cones. The information gained from quantitative genetic analysis can simplify the hop breeding process, improving estimates of the genetic gains that can be anticipated through selection methods and assisting with the development of clearly defined breeding aims for hop improvement. Hop growers can also be informed as to how a crop can be managed more efficiently through the control of environmental factors [3].

Hop is one of four essential ingredients of beer (the others being water, yeast and a carbohydrate source such as barley or wheat), added to provide bitterness, flavour and aroma as well as functioning as a natural preservative [6]. Female hop plants develop strobili (commonly called cones), which contain numerous glandular trichomes (lupulin glands) on their bracts [5,7,8]. The lupulin glands contain many secondary metabolites, including resins, essential oils and tannins [9]. The resins found in hop lupulin glands have not been found in any other plant species [9]; they comprise hard resins (including xanthohumol, iso-xanthohumol and flavone) and soft resins (also called hop acids), which are dominated by humulones (α-acids) and lupulones (β-acids) [10,11]. It is the α-acids that provide the bitter taste to beer [9]. β-acids also contribute to beer bitterness, as well as providing preservative activity [9,12-14]. The flavour and aroma of beer is derived from the hop essential oils, the composition of which is diverse (with more than 500 different compounds identified), but typically consisting of 90% terpenoids, dominated by myrcene, humulene, caryophyllene and farnesene [9-11,15,16]. Hop cultivars differ in their secondary metabolite profiles, in terms of the presence, amount and relative proportions of these compounds. As such, different hop cultivars produce different levels of bitterness and a variety of flavours and aromas [17,18]. Hop plants are perennial, wind-pollinated climbers, cultivated on strings suspended from a trellis [9]. Flowering is induced by shortening day length, after the plant has grown a minimum number of nodes [19,20]. Flowers develop at the terminal buds of lateral branches; female flowers develop into cones, which mature at the beginning of autumn [5,20]. The vegetative parts of the plant die back each year; the underground rootstock remains dormant over winter and re-sprouts in spring [9]. Hops have a native distribution between latitudes of approximately 35° and 70° North, from Western Europe, east to Siberia and Japan and across North America, except in highlands and deserts [21,22], but many hop cultivars are of European genetic origin, or are hybrids between European and North American germplasm [9,23].

Since the 1950s, several studies have examined the inheritance of quantitative traits in hop. Both clonal and progeny trials have been used to examine the heritability of traits relating to yield, including yield of cones (green or dry mass) per hectare and number of cones per plant [24-30]; cone chemistry and brewing quality, including α-acid, β-acid, their components and their relative proportions, as well as several essential oils [24,26-28,30-38]; and agronomic attributes, including hop storage index, morphology of cones, leaves, lateral and lupulin glands, vigour, flowering and cone maturity times and disease susceptibility [25,26,28-30,32,33,35,36,38,39]. These studies have documented a wide range of heritability estimates and variable genetic relationships between traits, and have generally found that hop cone chemistry, yield and plant morphology traits have a genetic basis. Many of the earliest of these studies estimated the inheritance of traits on the basis of phenotypic observation of the transmission of traits from parent to offspring, using little statistical analysis [30,32-34,36,37]. As such, these studies were unable to make full use of the information to separate genetic and environmental influences and therefore may be less reliable. Of those studies based on more sophisticated statistical procedures, the majority report broad-sense heritability and describe correlations on the basis of the total genetic variation [27-29,35,38,39]. Although the estimation of broad-sense heritability is able to discern between variation resulting from genotypic and environmental factors, it does not partition the genetic factor into additive, dominance and epistatic components [1,2]. The additive genetic component, which is based on the average effects of alleles, is the easiest type of genetic effect to predict and use in breeding [1,2]. As such, it is the only portion of genetic variation that is relevant to selection in current hop breeding programs [40].

Four studies have examined additive genetic variation in hop traits and have reported estimates of narrow-sense heritability (based only on additive genetic variation) [24-26,31]. These studies have examined 13 traits: five relating to hop acids (α-acid, β-acid, α-acid:β-acid, cohumulone and colupulone), five relating to essential oils (essential oil content, myrcene, β-caryophyllene, farnesene and humulene:β-caryophyllene), one relating to polyphenols (xanthohumol), one relating to yield (yield of dry cones) and one relating to agronomic attributes (hop storage index) [24-26,31]. While these studies provide information for selection of cone chemistry, hop storage index and yield, the inheritance of plant growth and agronomic suitability, as well as the relationship of these factors to cone chemistry and yield, has not been examined. These four studies report heritability estimates and genetic correlations that are derived from more accurate methods of calculation, but they are based on progeny trials consisting of too few families (12-25) [24-26,31] for the accurate estimation of quantitative genetic parameters [2,41]. Additionally, the families examined in these four studies were derived from a narrow genetic base, using parents of primarily European genetic origin [2,41]. As such, these results have to be treated carefully. Further heritability estimates and genetic correlations, from quantitative genetic analyses that includes a broader range of material and larger trials than those previously conducted, would expand our current understanding of the inheritance and genetic control of traits relating to cone chemistry, yield and agronomic characteristics in hop.

QTL have been identified for a number of traits relating to hop cone chemistry and yield, including α-acid and β-acid, as well as their components and relative proportions; total essential oil content and a number of individual essential oils; the polyphenols xanthohumol and desmethylxanthohumol; yield of dry cones; cone harvest index; and powdery mildew susceptibility [42-47]. These QTL indicate that variation in these traits has a genetic basis; but as many of these QTL have been identified in a single pedigree, environment and ontogenetic stage, a quantitative genetic analysis could offer insight into the degree of heritability of these traits in a broader range of hop material. Many of the QTL that have been identified for hop traits have been found to co-locate [42]. Quantititative genetic analyses could provide additional information about genetic correlations between traits, furthering the understanding of the genetic control of hop and providing important information for selective improvement of hop.

This study reports estimates of quantitative genetic parameters for 20 commercially important hop traits. Traits were selected on the basis of their relevance to hop breeding programs, and included α-acid and β-acid, two key brewing chemicals that impart the bitter taste and preservative activity to beer [9,12-14], as well as their components and relative proportions. Ten plant growth traits relating to agronomic features of the hop plant were evaluated, including traits related to emergence, height, lateral morphology and cone distribution. These agronomic traits are important for the cultivation of the hop plant, as well as being possible proxy selection indicators for chemical traits, where a correlation occurs. Yield of hop cones was evaluated by the weight of green cones per plant. The quantitative genetic parameters that were assessed included additive genetic variance and narrow-sense heritability, as well as the genetic correlations between traits and the degree to which variation and correlation of traits was affected by factors other than additive genetic effects (including the environment, agricultural practice, dominance, epistasis and error). Calculations of genetic parameters were based on a progeny trial consisting of the largest number of families (108) utilised for this purpose in hop, and families were derived from a broad genetic base of genotypes from European and North American genetic origins, as well as hybrids between the two. This study aims to increase our understanding of the inheritance of quantitative traits in hop as well as the genetic relationships between traits and the influence that elements besides additive genetic effects have on these factors. Such results would provide hop breeders with important information to assist selection and genetic gain in key traits, and would be of use in the planning of breeding programs for the development of superior hop cultivars.

## Results

### Genetic variation

Significant genetic variation was found between families for all cone chemical traits assessed (Table 1). For some cone chemical traits (colupulone, α-acid and β-acid) genetic variance was significant in only the first growing season (Table 1). No significant genetic variation was found between families for cone yield (assessed in only the second growing season) (Table 1); but significant genetic variation was found between families for all plant growth traits (Table 1). For one plant growth trait (internode length) significant genetic variance was detected in only the first growing season. For all traits, *C*V_A_ ranged from 0 to 2.67 (mean 0.12) (Table 1). The family least squares mean for each trait is reported in Additional file 1.

**Table 1:** Genetic variation and heritability of traits associated with cone chemistry, cone yield and plant growth in hop

			**Variance components**							**Fixed effects**				**Heritability**	
	**Trait**	**Age (months)**	**Rep.iblock**	**Additive**	**Error**	**V**_**P**_	***C*** **V**_**A**_	**t**	**Pr > t**	**Rep**	**P > F**	**Sex**	**P > F**	***h***^***2***^	**SE**
Plant growth	Number of shoots	11	0.09	0.06	0.88	1.03	0.05	2.75	P < 0.005	8.53	P < 0.0001	885.16	P < 0.0001	0.06	0.02
		24	0.56	0.40	4.97	5.93	0.09	2.87	P < 0.005	3.22	P < 0.05	1556.87	P < 0.0001	0.07	0.02
	Length of the longest shoot	11	0.32	0.33	5.86	6.52	0.01	2.31	P < 0.05	2.84	P < 0.05	1674.14	P < 0.0001	0.05	0.02
		24	0.09	0.37	1.84	2.31	0.05	4.28	P < 0.0001	2.39	NS	661.01	P < 0.0001	0.16	0.03
	Number of nodes on the longest shoot	11	0.56	0.40	4.97	5.93	0.09	2.87	P < 0.005	3.22	P < 0.05	1556.87	P < 0.0001	0.07	0.02
		24	0.03	0.03	0.23	0.29	0.06	3.38	P < 0.0005	0.74	NS	1407.14	P < 0.0001	0.10	0.03
	Height (at flower initiation)	13	0.05	0.09	1.32	1.45	0.08	2.73	P < 0.005	11.27	P < 0.0001	2550.49	P < 0.0001	0.06	0.02
		25	0.00	0.10	0.81	0.91	0.06	3.91	P < 0.0001	2.36	NS	7621.12	P < 0.0001	0.11	0.03
	Height (mid-season)	14	0.51	0.07	1.21	1.79	0.06	2.30	P < 0.05	1.55	NS	1177.69	P < 0.0001	0.04	0.02
		26	0.00	0.03	0.33	0.36	0.03	3.57	P < 0.0005	1.08	NS	23768.91	P < 0.0001	0.09	0.02
	Height (at cone maturity)	16	0.01	0.07	0.60	0.68	0.06	3.77	P < 0.0001	2.72	P < 0.05	7756.84	P < 0.0001	0.10	0.03
		28	0.01	0.08	0.58	0.66	0.06	4.03	P < 0.0001	2.94	P < 0.05	7709.27	P < 0.0001	0.12	0.03
	Lateral length	16	0.10	0.38	7.17	7.65	0.01	2.28	P < 0.05	0.77	NS	3218.57	P < 0.0001	0.05	0.02
		28	2.58	0.21	2.96	5.74	0.01	2.50	P < 0.01	0.27	NS	511.01	P < 0.0001	0.04	0.01
	Number of nodes on lateral	16	0.01	0.02	0.32	0.35	0.02	2.11	P < 0.05	1.51	NS	4820.12	P < 0.0001	0.04	0.02
		28	0.01	0.02	0.32	0.35	0.02	2.06	P < 0.05	1.50	NS	4851.78	P < 0.0001	0.04	0.02
	Internode length	16	0.00	0.06	0.23	0.29	0.01	2.31	P < 0.05	1.51	NS	4477.69	P < 0.0001	0.20	0.08
		28	1.39	0.00	1.82	3.21	0.00	0.00	NS	0.06	NS	216.63	P < 0.0001	0.04	0.02
	Height to the cones	16	0.01	0.03	0.33	0.38	0.10	2.54	P < 0.005	612.18	P < 0.0001	NA	NA	0.08	0.03
		28	0.01	0.04	0.32	0.37	0.12	2.86	P < 0.005	590.26	P < 0.0001	NA	NA	0.11	0.04
Yield	Green cone weight	28	0.01	0.00	0.11	0.12	0.02	0.33	NS	243.57	P < 0.0001	NA	NA	0.03	0.10
Cone chemistry	Cohumulone	16	0.00	0.03	0.07	0.10	0.06	4.14	P < 0.0001	1076.93	P < 0.0001	NA	NA	0.29	0.06
		28	NA	0.02	0.08	0.09	0.05	1.74	P < 0.05	895.61	P < 0.0001	NA	NA	0.18	0.10
	Humulone + adhumulone	16	0.00	0.04	0.12	0.16	0.03	3.93	P < 0.0001	1714.16	P < 0.0001	NA	NA	0.26	0.06
		28	0.00	0.02	0.12	0.15	0.03	1.72	P < 0.05	1250.91	P < 0.0001	NA	NA	0.17	0.09
	Colupulone	16	0.00	0.02	0.07	0.09	0.06	3.39	P < 0.0005	1105.00	P < 0.0001	NA	NA	0.21	0.06
		28	0.00	0.01	0.07	0.08	0.04	1.38	NS	950.66	P < 0.0001	NA	NA	0.15	0.10
	Lupulone + adlupulone	16	0.00	0.02	0.07	0.09	0.07	3.60	P < 0.0005	1076.23	P < 0.0001	NA	NA	0.23	0.05
		28	0.00	0.02	0.06	0.08	0.06	1.93	P < 0.05	868.60	P < 0.0001	NA	NA	0.21	0.10
	α-acid	16	0.00	2.00	5.41	7.41	0.16	3.99	P < 0.0001	503.89	P < 0.0001	NA	NA	0.27	0.06
		28	0.01	0.02	0.11	0.14	0.02	1.45	NS	1051.50	P < 0.0001	NA	NA	0.16	0.10
	β-acid	16	0.00	0.03	0.12	0.15	0.04	3.34	P < 0.001	1320.40	P < 0.0001	NA	NA	0.20	0.05
		28	0.00	0.03	0.17	0.21	0.04	1.56	NS	1319.15	P < 0.0001	NA	NA	0.15	0.10
	Cohumulone (% of α-acid)	16	0.00	0.00	0.00	0.00	0.11	3.90	P < 0.0001	2855.30	P < 0.0001	NA	NA	0.26	0.06
		28	NA	0.72	0.00	0.72	2.67	2.72	P < 0.005	3333.97	P < 0.0001	NA	NA	0.29	0.09
	α-acid:β-acid	16	NA	0.01	0.05	0.06	0.05	3.32	P < 0.001	1673.84	P < 0.0001	NA	NA	0.20	0.05
		28	0.00	0.03	0.12	0.16	0.10	2.03	P < 0.05	500.11	P < 0.0001	NA	NA	0.21	0.10
	α-acid:total resin	16	NA	0.00	0.00	0.00	0.04	3.52	P < 0.0005	11960.40	P < 0.0001	NA	NA	0.22	0.05
		28	0.00	0.00	0.00	0.00	0.05	2.05	P < 0.05	5383.75	P < 0.0001	NA	NA	0.22	0.10

'Age’ refers to the time that each trait was assessed after the trial was planted. 'Rep.iblock’ refers to the random effect of replicate.incomplete-block. 'Additive’ refers to additive genetic variance. 'Error’ refers to the random effect of residuals. 'V_P_’ refers to the phenotypic variance. '*C*V_A_’ refers to the coefficient of additive genetic variance. 't’ refers to the t-value for Additive and 'Pr > t’ refers to its significance. 'Rep’ and 'Sex’ refers to the fixed effects of replicate and plant sex on the trait, respectively; 'P > F’ refers to their significance in each case. '*h*^*2*^’ refers to the narrow-sense heritability and 'SE’ refers to standard error of *h*^*2*^.

The heritability of all traits assessed in the study ranged from 0.03 to 0.29 (mean 0.14) (Table 1). The heritability of cone chemical traits ranged from 0.15 to 0.29 (mean 0.22) and were generally higher than the heritability of growth traits, which ranged from 0.04 to 0.20 (mean 0.08) (Table 1). Cone yield displayed a very low heritability (*h*^*2*^ = 0.03) (Table 1). Estimates of heritability of cone chemical traits were generally higher in the first season of growth, along with plant growth traits related to lateral branch morphology (Table 1). The remaining plant growth traits had higher heritability estimates in the second season (Table 1).

The effect of replicate was significant for all of the cone chemical traits and also for many of the plant growth traits assessed (Table 1). The effect of sex was highly significant (P < 0.001) in all traits that were assessed in both male and female plants (all plant growth traits except height to the cones) (Tables 1 and 2). For all traits related to emergence, male and female phenotypes were similar in the first season of growth (assessed in the first month of spring), but in the second season (the last month of spring), male plants had significantly greater number of shoots, greater number of nodes on the longest shoot and a longer length of the longest shoot (Table 2). The heights of male and female plants were also significantly different throughout the growing season, with female plants being taller than male plants (Table 2). In terms of lateral morphology, female plants had significantly longer lateral lengths (in season one) and greater number of nodes on laterals (both seasons), but displayed similar internode lengths to male plants (Table 2).

**Table 2:** Differences between male and female hop plants for growth traits

**Trait**	**Age (months)**	***n *** **female plants**	**Female plants mean ± SD**	***n *** **male plants**	**Male plants mean ± SD**	**P value**
Number of shoots	11	671	5.51 ± 4.92	378	5.50 ± 4.33	NS
	24	671	6.49 ± 6.15	378	9.06 ± 6.51	P < 0.0001
Length of the longest shoot	11	606	51.15 ± 33.79	359	51.84 ± 31.32	NS
	24	496	10.79 ± 10.66	327	13.21 ± 12.05	P < 0.005
Number of nodes on the longest shoot	11	606	7.04 ± 2.44	359	7.21 ± 2.44	NS
	24	496	2.80 ± 1.76	327	3.28 ± 1.90	P < 0.0005
Height (at flower initiation)	13	670	3.74 ± 1.29	376	3.58 ± 1.13	P < 0.05
	25	669	5.15 ± 1.00	378	5.03 ± 0.87	P < 0.05
Height (mid-season)	14	624	4.71 ± 1.43	352	3.99 ± 1.17	P < 0.0001
	26	669	5.87 ± 0.46	378	5.34 ± 0.80	P < 0.0001
Height (at cone maturity)	16	666	4.77 ± 0.79	376	4.10 ± 0.90	P < 0.0001
	28	663	4.79 ± 0.76	376	4.18 ± 0.70	P < 0.0001
Lateral length	16	650	50.12 ± 26.57	362	44.80 ± 21.37	P < 0.0005
	28	649	44.65 ± 29.02	333	45.12 ± 20.90	NS
Nodes on lateral	16	650	6.73 ± 3.89	356	6.01 ± 2.41	P < 0.0005
	28	650	6.81 ± 3.41	357	6.08 ± 2.77	P < 0.0005
Internode length	16	152	17.53 ± 4.21	79	17.19 ± 4.88	NS
	28	228	28.04 ± 23.16	111	24.46 ± 18.31	NS

Each trait was assessed in two seasons of plant growth; 'Age’ refers to the age of the plants at the time that each trait was assessed after the trial was planted. 'Female plants *n*’ refers to the number of female plants assessed for each trait. 'Female plants mean ± SD’ refers to the phenotypic mean and standard deviation of all female plants for each trait. 'Male plants *n*’ refers to the number of male plants assessed for each trait. 'Male plants mean ± SD’ refers to the phenotypic mean and standard deviation of all male plants for each trait. 'P value’ refers to the significance of similarity between phenotypic variances of female and male plants.

### Genetic correlations

Trait pairwise genetic correlations were used to investigate the genetic relationships between five cone chemical traits relevant to hop breeding. α-acid and β-acid were positively genetically correlated in the first growing season, but were not correlated in the second season (Table 3). In both seasons, α-acid was positively genetically correlated with α-acid:β-acid and α-acid:total resin, while β-acid was negatively genetically correlated with these traits (Table 3). The genetic correlations between α-acid: β-acid and α-acid: total resin were strongly positive in both growing seasons (Table 3). In both seasons, cohumulone (% of α-acid) was positively genetically correlated with α-acid and negatively genetically correlated with β-acid; consistent with these findings, cohumulone (% of α-acid) was positively genetically correlated with α-acid: β-acid and α-acid: total resin (Table 3). For all of the cone chemical traits assessed, strong positive genetic correlations were identified between assessments in the two growing seasons (Table 4a).

**Table 3:** Additive genetic and phenotypic correlations between cone chemical traits and plant growth traits in hop

		**α-acid**	**β-acid**	**Cohumulone (% of α-acid)**	**α-acid:β-acid**	**α-acid:total resin**
**a.**						
**α-acid**			**0.44** ± 0.04	0.02 ± 0.06	**0.37** ± 0.05	**0.39** ± 0.05
**β-acid**		**0.48** ± 0.15		0.00 ± 0.06	**-0.65** ± 0.03	**-0.63** ± 0.03
**cohumulone (% of α-acid)**		**0.17** ± 0.18	**-0.13** ± 0.20		0.02 ± 0.06	0.02 ± 0.06
**α-acid:β-acid**		**0.52** ± 0.15	**-0.50** ± 0.15	**0.29** ± 0.20		**0.97** ± 0.00
**α-acid:total resin**		**0.47** ± 0.15	**-0.55** ± 0.14	**0.27** ± 0.19	**1.00** ± 0.01	
**b.**						
**α-acid**			**0.31** ± 0.06	0.09 ± 0.07	**0.54** ± 0.05	**0.53** ± 0.05
**β-acid**		-0.08 ± 0.49		-0.05 ± 0.07	-**0.59** ± 0.05	**-0.20** ± 0.10
**Cohumulone (% of α-acid)**		**0.17** ± 0.33	**-0.20** ± 0.36		0.10 ± 0.07	**0.12** ± 0.07
**α-acid:β-acid**		**0.78** ± 0.25	**-0.60** ± 0.27	**0.19** ± 0.30		**0.95** ± 0.01
**α-acid:total resin**		**0.78** ± 0.26	**-1.00** ± 38.48	**0.30** ± 0.30	**0.96** ± 0.03	
**c.**						
	**Number of shoots**	**Length of the longest shoot**	**Height (at flower initiation)**	**Height (at cone maturity)**	**Height to the cones**	**Lateral length**
**Number of shoots**		**0.57** ± 0.02	**0.14** ± 0.25	**0.22** ± 0.03	0.03 ± 0.04	**0.14** ± 0.04
**Length of the longest shoot**	**0.87** ± 0.12		**0.28** ± 0.03	**0.23** ± 0.03	**-**0.02 ± 0.04	**0.12** ± 0.04
**Height (at flower initiation)**	**0.14** ± 0.25	-0.01 ± 0.23		**0.42** ± 0.03	**0.13** ± 0.04	**0.18** ± 0.03
**Height (at cone maturity)**	**0.18** ± 0.21	**-0.20** ± 0.24	**0.77** ± 0.10		**0.24** ± 0.04	**0.34** ± 0.03
**Height to the cones**	**-0.22** ± 0.25	-0.01 ± 0.04	**0.62** ± 0.19	**0.66** ± 0.16		**0.21** ± 0.05
**Lateral length**	**0.22** ± 0.26	0.04 ± 0.28	**0.56** ± 0.19	**0.99** ± 0.12	**0.16** ± 0.26	

Pairwise additive genetic correlations and pairwise phenotypic correlations form the lower and upper parts of the matrix, respectively. The standard error of each correlation is given. Correlations statistically different from zero (*P* < 0.05) are shown in bold. a. refers to cone chemical traits assessed in the first year of plant growth (16 months after the trial was planted); and b. refers to cone chemical traits assessed in the second year of plant growth (28 months after the trial was planted). c. refers to plant growth traits assessed in the second year of plant growth; the traits number of shoots and length of the longest shoot were assessed in the first year of plant growth (11 months after the trial was planted); the traits height (at flower initiation), height (at cone maturity), height to cones and lateral length were assessed in the second year of plant growth (height (at flowering) at 25 months after the trial was planted and the remaining traits at 28 months after the trial was planted).

**Table 4:** Genetic and phenotypic correlations between the two growing seasons in which hop was assessed

	**Genetic correlations**	**Phenotypic correlations**
**a.**		
**α-acid**	**1.00** ± 0.24	**0.61** ± 0.07
**β-acid**	**0.99** ± 0.09	**0.85** ± 0.02
**Cohumulone (% of α-acid)**	**0.99** ± 0.10	**0.86** ± 0.02
**α-acid:β-acid**	**0.97** ± 0.12	**0.81** ± 0.03
**α-acid:total resin**	**0.95** ± 0.09	**0.86** ± 0.02
**b.**		
**Number of shoots**	**0.80** ± 0.12	**0.42** ± 0.03
**Length of the longest shoot**	**0.78** ± 0.15	**0.40** ± 0.03
**Height (at flower initiation)**	**0.94** ± 0.12	**0.45** ± 0.03
**Height (at cone maturity)**	**1.00** ± 0.01	**1.00** ± 0.01
**Height to the cones**	**1.00** ± 0.28	**1.00** ± 0.02
**Lateral length**	**1.00** ± 0.03	**0.69** ± 0.03

The genetic and phenotypic correlations were assessed between years was assessed for cone chemical traits and plant growth traits. The standard error of each correlation is given. Correlations statistically different to zero (*P* < 0.05) are shown in bold. a. refers to cone chemical traits, which were all measured at 16 months (season 1) and 28 months (season 2) after the trial was planted. b. refers to plant growth traits. The traits number of shoots and length of longest shoot were assessed 11 months (season 1) and 24 months (season 2) after the trial was planted. The trait height (at flower initiation) was assessed at 13 months (season 1) and 25 months (season 2) after the trial was planted. The traits height (at cone maturity), height to the cones and lateral length were assessed at 16 months (season 1) and 28 months (season 2) after the trial was planted.

Genetic relationships between hop cone chemistry and plant growth were also investigated in this study. Limited genetic correlation was observed between the emergence traits and the cone chemical traits. Number of shoots was negatively genetically correlated with β-acid and positively genetically correlated with cohumulone (% of α-acid) and α-acid:β-acid, but these correlations were only weakly significant (Table 5a). There was a weak positive genetic correlation between length of the longest shoot and α-acid:β-acid; and a stronger positive genetic correlation between length of the longest shoot and cohumulone (% of α-acid) (Table 5a). There was a higher degree of genetic correlation between the other plant growth traits and cone chemistry. Height, assessed at flowering and at cone maturity, was negatively genetically correlated with all chemical traits (Table 5a). Similar results were observed for the relationships between cone chemistry and the other two traits assessing plant growth at cone maturity: height to the cones and lateral length. Height to the cones was negatively correlated with all chemical traits, except cohumulone (% of α-acid) for which the correlation was not significantly different from zero (Table 5a). Lateral length was negatively genetically correlated with both α-acid and β-acid, but the negative correlation was stronger with β-acid than with α-acid (Table 5a). As a result, lateral length was positively genetically correlated with α-acid:total resin (Table 5a). Lateral length was not significantly genetically correlated with either cohumulone (% of α-acid) or α-acid:β-acid (Table 5a).

**Table 5:** Genetic and phenotypic correlations between cone chemical traits, cone yield and plant growth traits in hop

	**Number of shoots**		**Length of the longest shoot**		**Height (at flower initiation)**		**Height (at cone maturity)**		**Height to the cones**		**Lateral length**	
	**Genetic correlation**	**Phenotypic correlation**	**Genetic correlation**	**Phenotypic correlation**	**Genetic correlation**	**Phenotypic correlation**	**Genetic correlation**	**Phenotypic correlation**	**Genetic correlation**	**Phenotypic correlation**	**Genetic correlation**	**Phenotypic correlation**
**a.**												
**α-acid**	-0.05 ± 0.34	0.06 ± 0.07	0.03 ± 0.34	0.09 ± 0.07	**-0.56** ± 0.29	-0.09 ± 0.07	**-0.82** ± 0.26	-0.08 ± 0.07	**-0.59** ± 0.35	**-0.26** ± 0.08	**-0.49** ± 0.37	-0.03 ± 0.07
**β-acid**	**-0.11** ± 0.34	-0.07 ± 0.07	-0.07 ± 0.34	-0.07 ± 0.07	**-0.42** ± 0.28	**-0.20** ± 0.07	**-0.55** ± 0.25	0.04 ± 0.07	**-0.22** ± 0.35	**-0.16** ± 0.08	**-0.62** ± 0.37	**-0.14** ± 0.07
**cohumulone (% of α-acid)**	**0.16** ± 0.25	0.10 ± 0.07	**0.45** ± 0.24	**0.25** ± 0.06	**-0.21** ± 0.21	0.07 ± 0.07	**-0.39** ± 0.19	0.03 ± 0.07	-0.07 ± 0.24	-0.01 ± 0.08	-0.04 ± 0.26	0.03 ± 0.07
**α-acid:β-acid**	**0.17** ± 0.29	0.11 ± 0.07	**0.18** ± 0.29	**0.13** ± 0.07	**-0.15** ± 0.24	0.10 ± 0.07	**-0.22** ± 0.23	-0.00 ± 0.07	**-0.22** ± 0.28	-0.07 ± 0.08	0.05 ± 0.29	0.05 ± 0.07
**α-acid:total resin**	0.04 ± 0.29	0.10 ± 0.07	0.01 ± 0.29	**0.13** ± 0.07	**-0.12** ± 0.24	0.10 ± 0.07	**-0.13** ± 0.22	-0.01 ± 0.07	**-0.30** ± 0.30	-0.05 ± 0.08	**0.12** ± 0.27	0.09 ± 0.07
**b.**												
				**Green cone weight**								
			**Genetic correlation**					**Phenotypic correlation**				
**α-acid**			**-0.93** ± 0.72					**0.22** ± 0.07				
**β-acid**			**-0.63** ± 0.53					0.05 ± 0.07				
**Cohumulone (% of α-acid)**			**-0.44** ± 0.09					0.05 ± 0.07				
**α-acid:β-acid**			**0.83** ± 0.21					**0.16** ± 0.07				
**α-acid:total resin**			**0.42** ± 0.19					**0.14** ± 0.07				
**c.**												
**Number of shoots**			**0.62** ± 0.30					**0.24** ± 0.82				
**Length of the longest shoot**			**-0.27** ± 0.79					0.05 ± 0.07				
**Height (at flower initiation)**			**-0.95** ± 0.37					**0.20** ± 0.07				
**Height (at cone maturity)**			**-1.00** ± 0.19					**0.21** ± 0.07				
**Height to the cones**			**-0.94** ± 0.34					**-0.94** ± 0.34				
**Lateral length**			**-0.93** ± 0.25					**-0.93** ± 0.25				

Cone chemical traits and yield were assessed in the second year of plant growth (28 months after the trial was planted). The plant growth traits number of shoots and length of the longest shoot were assessed in the first year of plant growth (11 months after the trial was planted) while height (at flower initiation), height (at cone maturity), height to the cones and lateral length were assessed in the second year of plant growth (height (at flower initiation) at 25 months after the trial was planted and the remaining plant growth traits at 28 months after the trial was planted). The standard error of each correlation is given. Correlations statistically different from zero (*P* < 0.05) are shown in bold. a. refers to genetic and phenotypic correlations between cone chemical traits and plant growth traits. b. refers to genetic and phenotypic correlations between cone chemical traits and yield. **c.** refers to genetic and phenotypic correlations between plant growth traits and yield.

The genetic relationships between the different plant growth traits were assessed, with positive correlations found between most traits (Table 3c). Exceptions to this were negative correlations between number of shoots and height to the cones, and length of the longest shoot and height at cone maturity; and no correlation between length of the longest shoot and the traits height at flowering, height to the cones and lateral length (Table 3c). The consistency of family performance for each growth trait was also assessed across the two growing seasons in which measurements were made. For all of the plant growth traits assessed, genetic correlations between different assessments of the trait were strongly positive (Table 4b).

In addition, the genetic relationships between cone yield and both the cone chemical traits and plant growth traits were assessed. Green cone weight was found to be negatively genetically correlated with α-acid, β-acid and cohumulone (% of α-acid), but positively genetically correlated with α-acid:β-acid and α-acid:total resin (Table 5b). Green cone weight was positively genetically correlated with the emergence trait number of shoots, but was negatively genetically correlated with another emergence trait length of the longest shoot (Table 5c). Green cone weight was negatively genetically correlated with height measured at flowering, but positively genetically correlated with height measured at cone maturity (Table 5c). Green cone weight was negatively genetically correlated with both height to the cones and lateral length (Table 5c).

### Phenotypic correlations

The phenotypic relationships at the family level between cone chemistry, cone yield and plant growth traits in hop were also investigated to give an indication of the influence of factors other than additive effects (including environmental and agronomic factors, as well as non-additive genetic effects and error) on these traits. Pairwise testing of the chemical traits found positive phenotypic correlations between α-acid and β-acid in both growing seasons (Table 3), indicating an influence of factors other than additive genetic effects in at least the second season (where no genetic correlation was identified). Other combinations of traits for which factors other than additive genetic effects were found to have an influence on phenotypic correlations included cohumulone (% of α-acid) with each of the traits β-acid, α-acid:β-acid and α-acid:total resin (first season only; Table 3). Pairwise phenotypic correlations between all other cone chemical traits were similar to the genetic correlations identified earlier.

Factors other than additive genetic effects were clearly found to influence hop plant growth, evidenced by the results of pairwise tests between cone chemical traits and the plant growth traits. Either no significant phenotypic correlation was found between traits where a significant genetic correlation had been identified, or the significance of the phenotypic correlation was lower than the significance of the genetic correlation (Table 5a). The only exception to this was a significant phenotypic correlation between length of the longest shoot and α-acid:total resin, where no significant genetic correlation was identified (Table 5a). This trend was generally true for phenotypic correlations between cone yield and cone chemical traits and cone yield and plant growth traits, with exceptions being the relationships between green cone weight and each of the traits α-acid, height at flowering and lateral length; all of these traits were found to be strongly negatively genetically correlated but positively phenotypically correlated with green cone weight (Table 5).

The phenotypic relationships between the different plant growth traits were generally similar to the genotypic correlations, indicating that factors besides additive genetic effects had a relatively small effect on the correlations between these traits (Table 3c). The exceptions to this were correlations between length of the longest shoot and the traits height at flowering, height at cone maturity and lateral length, where the traits were positively phenotypically correlated with length of the longest shoot, but no genotypic correlation was identified (Table 3c). The consistency of family performance for each growth trait was also assessed across the two growing seasons in which measurements were made. For each of the plant growth traits assessed, the phenotypic correlations between the assessments of the trait were positive across the two growing seasons (Table 4b).

## Discussion

### Genetic variation

This study found heritable genetic variation between families in the key hop brewing substances α-acid and β-acid, as well as their components (cohumulone, humulone + adhumulone, colupulone and lupulone + adlupulone) and their relative proportions (cohumulone (% of α-acid) α-acid: β-acid and α-acid: total resin) (Table 1). Heritable genetic variation between families was also identified for morphological features of hop plant growth fundamental to optimal agronomic management, including emergence, height, lateral growth and distribution of cones over the hop plant (Table 1). Of those traits for which heritable variation was identified, the narrow-sense heritability estimates ranged from 0.04 to 0.29, with a mean of 0.15 (Table 1). Cone chemical traits generally had higher heritability than growth traits (Table 1). This may reflect the intense selection directed at hop cone chemical traits compared to growth traits. Selection of hop cone chemical traits could be due to both artificial selection by breeding or natural selection as a result of the rapid co-evolution of chemical profiles and herbivore tolerance traits [48,49]. In addition, growth traits are likely to be more susceptible to environmental/agronomic influences. The estimates of narrow-sense heritability for cone chemical traits were generally lower in this study compared to those calculated for similar traits in previous studies of hop [24,26,31] (Additional file 2a). The exception to this was the value of zero for heritability of α-acid:β-acid reported by Murakami [31], compared to the estimate of 0.21 calculated in this study (Additional file 2a). The variability of the estimates reported illustrates the fact that heritability is a function of the genetic material upon which the calculation is based. There are several factors pertaining to the experimental design of this study that could explain the generally lower heritability estimates observed compared with the previous studies in hop. Firstly, this study utilised 108 families for estimation of heritability. Perron et al. [41] and Lynch and Walsh [2] have found that at least 75 families are generally required for accurate estimation of genetic parameters. Previous hop studies have used far fewer than 75 families [24,26,31] (Additional file 2a), potentially inflating estimates. Secondly, the families used in this study were generated from open-pollination (and open-pollination also occurred extensively within the pedigree of their ancestors), rather than controlled crosses as in the previous studies [24,26,31]. This may have increased variability within half-sib families, decreasing heritability estimates. Thirdly, open-pollination may have reduced the accuracy of the relationship matrices, as the fathers of each family are unknown. Besides the missing parental information, the models for the calculation of heritability assume that the unknown fathers are unrelated, which is highly unlikely. These factors mean that the population size is likely to be smaller than that designated in our model, resulting in decreased heritability estimates. Inaccuracies in the relationship matrix may also have arisen due to missing information in the pedigree, where the ancestry for particular individuals (e.g. founders) is unknown. Fourthly, as suggested earlier, there is likely to be a high level of inbreeding among the parents of this study population. The models for calculation of heritability assume that the founders in our pedigree are unrelated, but this is unlikely as it is well documented that most hop cultivars descend from relatively few common ancestors that were highly prized for their brewing properties [9,50,51]; these cultivars have been found to have relatively limited genetic variability between them [50]. Inbreeding within the population would again result in a smaller population size than that designated in our model, possibly resulting in decreased heritability estimates. In addition, variability in the maturity of cones may have distorted the level of variation within families, as the levels of many chemical traits have been found to change during cone maturation [52]. However, this is also likely to have been an issue in previous hop quantitative genetics studies.

While the heritability estimates reported in this study are possibly underestimates, the findings from this study may be more broadly applicable to hop as a species, as estimates were based on a larger number and greater diversity of families than any previous study [24,26,31] (Additional file 2a). Studies which have examined the genetic diversity of hop have determined two primary genetic groupings: European and North American [53-61]. The genotypes used in previous hop studies [24,26,27,31] were largely of European genetic origin and from a relatively narrow genetic base. In this study, genotypes of both European and North American origin were included, as well as hybrids between the two groups (Additional file 3). The accuracy of estimates of genetic parameters from this study could be improved by classifying the genotypes in the pedigree into groups to reflect the European/North American population structure; however, while the families in this study were supported by extensive pedigree information (often going back as far as founders), the records were not adequate to classify every founder or genotype into a genetic group. Accurate genetic groups could be assigned in future studies with the aid of molecular data (as in Steane et al. [62]) to improve the estimation of genetic variance. In addition, similar quantitative genetics trails incorporating European and North American genetic diversity could be simultaneously conducted in European, North American and Australian environments to provide more insight into the genetic control of key hop traits.

This study is the first to report estimates of narrow-sense heritability for growth traits in hop. This assessment of the potential heritable genetic variation in growth traits provides important information for the development of new hop cultivars with improved agronomic characteristics, such as timely emergence, appropriate growth and maximal distribution of hop cones on the bine. This study also revealed an influence of sex on hop growth. Male and female hop plants have been described as being indistinguishable until they switch from the vegetative phase to the reproductive phase [5]; however this study found, for the first time, significant phenotypic differences in the growth of male and female plants as early as the emergence of shoots, in terms of the number, length of the longest shoot and number of nodes on the longest shoot (Tables 1 and 2). Male and female plants continued to display differences in morphology throughout the growing season and at maturity, for a range of plant growth traits, including height and elements of lateral morphology (Tables 1 and 2). Only a few dioecious plant species have been described as sexually dimorphic in vegetative morphology, including *Salix arctica*, *Acer negundo, Simmondsia chinensis* and *Phoradendron juniperinum*[63]*.* In these species, differences in photosynthetic rate and transpiration (both key traits underlying agronomic performance) between male and female plants were the cause of the observed differences in morphology [63]. An early study in hop investigated the physiological differences between male and female plants, finding differences in transpiration rate, but not in photosynthetic rate [64], however further work is required to confirm this. The sexual dimorphism in growth found in this study suggests that there might be differences in these key physiological traits in hop, providing an opportunity to further investigate the genetic control of photosynthetic rate and water use efficiency in hop.

No significant heritable genetic variation was identified between families for yield of hop cones (green cone weight) (Table 1). An explanation for this might be suboptimal agricultural management of the hop plants early in the cultivation process. Hop cultivars produce uniform yields, but these yields are dependent on flowering at the optimum time, which is in turn dependent on bine control and training up the trellis at the appropriate time [20]. Flowering in hop is triggered by shortening daylength [19,20]; and different cultivars vary in their photoperiod requirements, as well as the optimum number of growing days from initial bine training to flowering and from flowering to cone maturity [20]. Yield may be significantly reduced where the bine training date is not optimal for a particular genotype on a particular site. Backdating from cone maturity to determine the optimum training date for each individual in progeny trials, such as this one, is not feasible. This may have had a significant effect on yield, distorting the level of variation within families (Additional file 1d.) and resulting in no significant genetic variation between families (Table 1). Heritable genetic variation for yield has been reported in previous studies of hop (Additional file 2a) [24,26], but these trials consisted of fewer families derived from controlled crosses (reducing the genetic variability within families). As such, it is highly likely that emergence times in these trials were more uniform. Yield variance may also have been affected by inbreeding. If there was a high level of inbreeding among the parents of the study, this may reduce the variability of yield in the progeny trial [65,66].

### Genetic correlations

The brewing properties of hop cultivars are defined by the chemical composition of hop cones [9,12-14]. Of the chemical compounds that comprise hop resin, two of the most important are α-acid and β-acid. α-acids are the key source of bitterness in beer, while β-acids also contribute bitterness, but to a lesser extent [9]. The relative proportion of these compounds to each other is of high importance to the way that hops are used in brewing, with hops that have higher α-acid relative to β-acid ('high-alpha hops’) used in bitter beers, and hops that have more equivalent levels of α-acid to β-acid ('aroma hops’) traditionally used for (non-bitter) flavour and aroma [67,68]. This study examined the genetic interrelationships between α-acid and β-acid and their relative proportions (α-acid:β-acid and α-acid:total resin). α-acid and β-acid were found to be positively genetically correlated in the first season of plant growth, but no relationship between them was detected in the second season (Table 3). The lack of correlation in the second year is a positive factor for hop breeders, as it suggests that when hop plants reach maturity, the two compounds can be selected for independently, without changes to one compound influencing the other. α-acid was positively genetically correlated with α-acid:β-acid and α-acid:total resin while β-acid was negatively genetically correlated with these traits (Table 3), reflecting the trend that as levels of α-acid in hop resin increase relative to β-acid, these ratios increase, but indicating that this trend has a genetic basis. Accordingly, α-acid:β-acid was positively genetically correlated with α-acid:total resin (Table 3). The relationships between α-acid and β-acid reported in previous studies vary. Negative genetic correlations between the two compounds were reported by Henning et al. [25,26], but positive genetic correlations were reported by Henning and Townsend [24] (Additional file 2b). Murakami [31] examined the genetic relationships between α-acid, β-acid and α-acid:β-acid, but did not find a significant correlation between any of the traits.

Traditionally, hops with lower levels of cohumulone, one of the secondary metabolites that comprise α-acid, were considered more desirable for brewing purposes as it was thought that it contributed a harsh and unpleasant bitterness to the brew [69]. This idea probably stemmed from the fact that most 'noble hops’ (traditional hops from Europe, prized for their mild bitterness and pleasant aroma) have relatively low levels of cohumulone [67,70]. More recently, the role of cohumulone has been called into question, with studies showing that quality of bitterness was not adversely affected by cohumulone [71]; and new hop varieties developed with higher levels of cohumulone that are considered not to impart a harsh bitterness. This study found significant positive genetic correlations between α-acid and cohumulone (% of α-acid) and significant negative genetic correlations between β-acid and cohumulone (% of α-acid), despite there being a positive genetic correlation between α-acid and β-acid (Table 3). Significant positive genetic correlations were found between cohumulone (% of α-acid) and both the traits α-acid: β-acid and α-acid: total resin (Table 3). These findings may reflect the history of selection for lower levels of cohumulone in 'noble’-type hops, as where α-acid: β-acid and α-acid: total resin ratios are low, the proportion of cohumulone in α-acid is also low. Previous studies have not examined the relationships between cohumulone as a percentage of α-acid and other chemical traits. Strong positive genetic correlations between seasons were found for each of the chemical traits assessed (Table 4a), indicating that families are highly consistent season to season in their chemical profiles.

While the analytical bitterness potential of hops are easily analysed, the bittering, flavour and aroma properties of hops are difficult to ascertain prior to brewing [72,73]. Attempts have been made to develop methods of selection (such as using molecular markers) that can be used to evaluate hop genotypes for particular chemical profiles prior to trial-brewing [42-44,46]. Understanding genetic correlations can be used to identify potential proxy selection indicators where it is difficult or expensive to measure traits directly, or to avoid potentially unfavourable consequences that would arise from the selection of seemingly unrelated traits [74]. This study examined the genetic relationships between key cone chemical traits and plant growth traits, to determine whether morphological characteristics could be used as proxy selection indicators for particular chemical attributes. A significant negative genetic relationship was found between plant vigour (characterised by a greater number of shoots at emergence, taller plants at both flower initiation and cone maturity, as well as plants with longer laterals) and α- and β-acids, where families with increased plant vigour tended to have decreased levels of α-acid and β-acid (Table 5a). Significant positive genetic correlations were found between all of these growth traits and between measurements across seasons, indicating that vigour is maintained throughout the growing season and over years (Tables 3c and 4b). These findings indicate that plant vigour could be used as an indicator of α-acid and β-acid levels in hops, with selection of families that have low vigour likely to also have higher levels of α-acid and β-acid.

The association of increased vigour with lower levels of α- and β-acids may reflect the underlying population structure of families included in this study, as well as the influence of past selection in the Australian environment. As discussed earlier, the families included in this study consisted of genotypes from both European and North American genetic groups. It has been observed that hops of European genetic origin tend to have more vigorous, leggy growth (i.e. greater heights and longer laterals) and lower levels of α-acid and β-acid when grown in the Australian environment, while hops of North American genetic origin tend to have less vigorous, more compact growth and higher levels of α-acid and β-acid when grown in the Australian environment. The genetic relationship between vigour and α- and β-acid observed in this study may be a reflection of the binary population structure of founder genotypes of European or North American genetic groups, or it may be indicative of selection for more compact growth and higher α- and β-acid levels.

Length of the longest shoot, one of the emergence traits assessed in this study, was not found to be associated with plant vigour. No genetic relationship was identified between this trait and any of the other growth traits, except for height at cone maturity, where there was a significant negative genetic correlation (Table 3c). Length of the longest shoot was not significantly correlated with α-acid or β-acid, but did have a significant positive genetic relationship with cohumulone (% of α-acid) (Table 5a). This relationship may again be a reflection of past selection in the Australian environment. Height to the cones was negatively genetically correlated with both α-acid and β-acid (Table 5a), indicating that families that tend to have smaller distances between the ground and where the bulk of the hops begin on the bine also have greater levels of α-acid and β-acid in their cones. Selection of plants with a shorter height to the cones would result in concomitant increases in α-acid and β-acid. Families that displayed a greater height to the cones tended to also have reached a greater height at flowering and at cone maturity (Table 3c).

The direct economic benefits of yield increases ensure that it is a core aim of every hop breeding program. Previous reports of the relationships between yield and the chemical traits α-acid and β-acid vary, with both positive and negative correlations between the traits reported [24-26] (Additional file 2b). Yield was genetically correlated with a number of cone chemical and plant growth traits in this study (Table 5); however, as yield was not found to be heritable in this study (Table 1), these genetic correlations should be treated with caution. This study, like all previous quantitative genetics studies on hop, was conducted in only a single environment and many of the traits were assessed in only a few years; as such our understanding of the influence of genetic and environmental interactions on traits is relatively limited. Future quantitative genetics studies in hop should encompass a greater number of years and environmental conditions to improve our understanding of these interactions.

### Phenotypic correlations

Differences between values of additive genotypic correlation and phenotypic correlation are indicative of the influence of factors other than additive genetic effects (such as the environment, agricultural practice, non-additive genetic effects and error) on the correlations between traits. In this study environmental variation was likely to have been an influential factor on the progeny trial, evidenced by replicate having significant effects on the variance of many of the traits assessed and a large proportion of the total variation attributed to replicate.incomplete block effects, particularly for plant growth traits (Table 1). Many of the correlations between cone chemical traits examined in this study appeared consistent at the phenotypic and additive genetic level (Table 3). There was evidence of influences other than additive genetic effects causing a correlation between α-acid and β-acid in one of the growth seasons, where no additive genetic correlation between the two traits had been identified (Table 3). There was also evidence of influences other than additive genetic effects masking the additive genetic correlations between cohumulone (% of α-acid) and all other cone chemical traits, with no phenotypic correlations but significant additive genetic correlations between the traits, identified (Table 3). Similarly, many of the growth traits examined in this study were significantly genetically correlated but not phenotypically correlated. The exceptions to this were relationships between length of the longest shoot and the traits height at flowering, height at cone maturity and lateral length, where factors besides additive genetic effects produced phenotypic correlations between the traits, where no additive genetic correlation had been identified (Table 3c). The influence of factors other than additive genetic effects was prominent in the correlations between plant growth and cone chemical traits, yield and cone chemical traits, and yield and growth traits, whereby traits that were significantly genetically correlated displayed no phenotypic correlation (Table 5). As stated earlier, one of these factors other than additive genetic effects could be dominance. Henning and Townsend [24] have shown that dominance does play a role in the expression of yield, and the chemical traits α-acid, β-acid and cohumulone. The influence of dominance is not well understood and warrants further investigation in hop. The results from this study do, however, indicate potential proxies for chemical traits which, with further testing, could be used as selection indicators in breeding programs.

## Conclusions

This study presents estimates of quantitative genetic parameters for 20 hop traits related to cone chemistry, cone yield and plant growth. Calculations were based on the largest number of families and on the broadest genetic base to be assessed in this kind of study in hop. This study revealed heritable genetic variation in cone chemistry and plant growth traits in hop. In comparison to previous findings, heritability estimates were lower for cone chemical traits, but estimates were based on a greater number of families and a more diverse genetic background, improving the accuracy of findings and offering a broader perspective on the inheritance of traits of economic importance in hop. This was the first study to report narrow-sense heritability for growth traits in hop, which were found to be generally lower than that of cone chemical traits, likely reflecting a more intense selection for cone chemistry and the greater influence of environmental factors on hop growth. Cone chemical traits were significantly genetically correlated with each other and with plant vigour, whereby increased vigour was associated with lower levels of α-acid and β-acid. This trend may reflect an underlying population structure of plants with European or North American genetic origin and past selection in the Australian environment. This requires further testing in additional environments. Factors other than additive genetic effects were found to have a significant impact on the correlations between traits, often masking genetic correlations. This study was also the first to report the effect of sex on phenotype on hop plants, as early as emergence. Male and female plants displayed differences in morphology throughout the growing season and at maturity. The findings from this study will provide breeders with a greater understanding of the genetic control of hop, information which will be useful for the selective improvement of the species.

## Methods

### Field trial

The genetic control of hop cone chemistry, cone yield and agronomic characteristics were investigated using a field trial at Bushy Park, Tasmania (42°42΄33˝S 146°53΄54˝E). The trial consisted of open pollinated seedlings from 160 female parents that included commercial cultivars and breeding lines from Australia, Europe and the USA. The female parents were pollinated in January 2008 and the seedlots collected in March 2008. The seeds were subject to stratification in July 2008 and were germinated in September 2008. The trial, planted in December 2008, was established in a randomised incomplete block design [75], comprising five replicates of 16 incomplete blocks. Each incomplete block contained 10 families in two-plant contiguous plots, giving a total of 10 plants per family. Families comprised both male and female plants, with an average of 6.2 (± 1.6 SD) female plants per family. Plants were grown in rows spaced 2.8 m apart and with 0.9 m between plants within each row (a planting density of ~3940 plants per hectare). Plants were grown up a 6 m trellis, with one string per plant and three bines trained up each string. Routine agricultural practice for hop in Australia was applied to the trial, including standard fertilisation, overhead irrigation and bine training by hand.

Families in the trial displaying evidence of monoecy (assessed by field observation) or polyploidy (assessed by pedigree record) were excluded from analyses. Analyses proceeded on the basis of 1049 individuals from 108 families (Additional file 3). A pedigree of the female parents and their ancestors, to founders where possible, was constructed using records from Roborgh [76], Homer et al. [77], Zimmermann et al. [78], Haunold et al. [79], Neve and Darby [80], Kenny and Zimmermann [81], Miyata [82], Jakše et al. [50], Patzak [83], Reed [84], California Fermentation Society [85], Freshops USDA named hop variety descriptions [86], Simply Hops [85], The Germplasm Resources Information Network (GRIN) [87] and Hop Products Australia.

### Trait measurements

Twenty traits were assessed in this study, including ten plant growth traits related to agronomic suitability (three associated with emergence and seven with vegetative morphology), nine traits evaluating cone chemistry and one trait assessing cone yield (Table 6). Plant growth traits and cone chemical traits were assessed in the trial over two cultivation seasons, while cone yield was assessed in one season (Table 6). Only female plants were used in the assessments of cone chemistry and cone yield. Where necessary, power transformations were used to standardise the variance of traits (Table 6).

**Table 6:** Plant growth, yield and cone chemistry traits assessed over two seasons of the hop cultivation process

	**Trait**	**Description**	**Units**	***n *** **individuals**	***n *** **families**	**Age (months)**	**Trans-formation**	**Mean**
Plant growth	Number of shoots	Number of shoots at emergence	Count	1049	108	11	x^0.5^	5.51
				1049	108	24	none	7.42
	Length of the longest shoot	Length of longest shoot at emergence	Centimetres	965	108	11	x^0.5^	51.41
				823	108	24	x^0.5^	11.67
	Number of nodes on the longest shoot	Number of nodes on shoot of maximal length	Count	965	108	11	none	7.11
				823	108	24	x^0.5^	2.99
	Height (at flower initiation)	Height of bine from ground to top of 6 m trellis, assessed at flower initiation	Metres	1046	108	13	none	3.68
				1047	108	25	none	5.10
	Height (mid-season)	Height of bine from ground to top of 6 m trellis, assessed between flowering and cone maturity	Metres	976	108	14	none	4.45
				1047	108	26	none	5.68
	Height (at cone maturity)	Height of bine from ground to the top of 6 m trellis, assessed at cone maturity	Metres	1042	108	16	none	4.53
				1039	108	28	none	4.54
	Hateral length	Hength of lateral at shoulder height	Centimetres	1012	108	16	x^0.5^	48.22
				982	108	28	x^0.5^	44.81
	Number of nodes on lateral	Number of nodes on same lateral measured for lateral length	Count	1006	108	16	x^0.5^	6.55
				1007	108	28	x^0.5^	6.56
	Internode length	Length of 3^rd^ internode from main bine, on same lateral measured for lateral length	Centimetres	231	78	16	x^0.5^	17.41
				339	96	28	x^0.5^	26.86
	Height to the cones	Height of bine from ground up to where bulk of cones began	Metres	663	108	16	none	1.74
				588	108	28	none	1.75
Yield	Green cone weight	Fresh weight of cones per plant	Grams	204	107	28	x^0.5^	1.02
Cone chemistry	Cohumulone	Cohumulone	% dry weight	397	108	16	x^0.5^	2.71
				208	107	28	x^0.5^	2.50
	Humulone + adhumulone	Humulone + adhumulone	% dry weight	397	108	16	x^0.5^	6.41
				208	107	28	x^0.5^	5.34
	Colupulone	Colupulone	% dry weight	397	108	16	x^0.5^	2.31
				208	107	28	x^0.5^	2.51
	Lupulone + adlupulone	Lupulone + adlupulone	% dry weight	397	108	16	x^0.5^	2.16
				208	107	28	x^0.5^	2.17
	α-acid	Cohumulone + humulone + adhumulone	% dry weight	397	108	16	none	9.12
				208	107	28	x^0.5^	7.84
	β-acid	Colupulone + lupulone + adlupulone	% dry weight	397	108	16	x^0.5^	4.48
				208	107	28	x^0.5^	4.69
	Cohumulone (% of α-acid)	Cohumulone/α-acid	% dry weight	397	108	16	x^0.5^	0.30
				208	107	28	x^0.5^	0.32
	α-acid:β-acid	α-acid/β-acid	Ratio	397	108	16	x^0.5^	2.21
				208	107	28	x^0.74^	1.80
	α-acid:total resin	α-acid/(α-acid + β-acid)	Ratio	397	108	16	x^0.5^	0.67
				208	107	28	x^0.5^	0.62

*n* individuals’ refers to the number of individuals assessed for each trait. '*n* families’ refers to the number of families assessed for each trait. 'Age’ refers to the age of the plants at the time that a trait was assessed in number of months after the trial was planted. 'Transformation’ refers to the power transformation used to standardise the variance of each trait (x). 'Mean’ refers to the backtransformed mean of all assessed individuals for each trait.

Cone samples for chemical analysis were collected at several days post commercial maturity of the majority of the trial. Commercial maturity was based on the stability of the α-acid:β-acid ratio, which increases as the cone matures and plateaus at maturity. This was assessed in a subset of the trial during cone maturation. Many hop chemicals (α-acid and β-acid) are more stable after maturity is reached than before [52]. Hop samples were dried for eight to 12 hours at 55°C. For each plant, hop cone chemical extracts were prepared by grinding 10 g of dried hop cone tissue using a domestic coffee grinder. A quantity of 2 g of the ground tissue was then extracted with 20 ml toluene in a 30 ml glass vial with 3 × 6 mm stainless steel ball-bearings using a rotator at 75 rpm for 30 min. The samples were allowed to stand for 10 min; then diluted 1:20 with an 85% methanol solution. An 800 μL aliquot of the dilution was placed in a 1 ml HPLC vial using a two syringe (2500 μL and 250 μL) Hamilton 500 series microdiluter. Diluted samples were vortexed for 3 sec before placing into a Waters 717 autosampler. Hop acids were fractionated by HPLC, on a system consisting of a Waters 1515 pump and column heater (29°C), a Waters 717 autosampler and a Waters 2996 UV/UV-VIS photodiode array detector at wavelengths of 325 nm (α-acids) and 342 nm (β-acids). A Varian ChromSpher reversed-phase C18 column (100 x 4.6 mm; 3 μm particle size) was used, coupled with a Varian 10 x 2 mm ChromSep guard column. Column temperature was maintained at 28°C. The mobile phase used for separation comprised 86% methanol (containing 0.1 g/L dissolved tetra-sodium EDTA) and 14% 0.05 M sulphuric acid; the flow rate was 1.2 ml/min, for 8 min per sample. The sample volume injected was 10 μL. Quantification was performed using the Waters Empower software package and the International Calibration Extract (ICE-3 ASBC) for reference. ICE-3 was prepared by dissolving 1.8 g of ICE-3 in 100 ml methanol; ICE-3 samples were then prepared for injection as per the methods for other samples. Three standard vials were run with each batch of samples, with each standard vial sampled six times (three times at the start and three times at the end of each run). Four components (cohumulone, humulone + adhumulone, colupulone, and lupulone + adlupulone) were identified and quantified. The other five cone chemical traits (α-acid, β-acid, cohumulone (% of α-acid), α-acid:β-acid and α-acid:total resin) were derived by calculation from these four components (as described in Table 6).

### Statistical procedures

ASReml [88] was used to conduct general linear mixed model analyses of the plant growth, cone yield and cone chemical data collected from the progeny trial. Residual maximum likelihood estimates of variance and covariance were obtained for each trait. The univariate model used was defined as:

$$y=X\beta +Z_{1}c+Z_{2}a+e$$

where **y** is the vector of *n* observations for the dependent variable; **β** is the vector of fixed effects, which were sex (only for the plant growth traits) (as performed by Gilmour [89]) and replicate; **c** is the vector of random replicate.incomplete-block effects; **a** is the vector of random additive genetic effects; and **e** is the vector of random residuals. **X**, **Z**_**1**_ and **Z**_**2**_ are incidence matrices relating observations to factors in the model. The variance for each component was defined as:

$$\begin{array}{c} \text{Var}\left[c\right]=C=I\sigma_{c}^{2}\\ \text{Var}\left[a\right]=G=A\sigma_{a}^{2}\\ \text{Var}\left[e\right]=R=I\sigma_{e}^{2} \end{array}$$

where **C**, **G** and **R** represent the random effects (family and the replicate.incomplete-block term), additive and residual covariance matrices between the observations respectively; ***A*** is the numerator relationship matrix for additive genetic effects; ***I*** is an identity matrix; and **σ**^**2**^_***x***_ is the variance of *x*. The expected values and variances of the model were as follows:

$$\begin{array}{cc} E\left[\begin{array}{l} y\\ c\\ a\\ e \end{array}\right]=\left[\begin{array}{l} \text{Xb}\\ 0\\ 0\\ 0 \end{array}\right], & \text{Var}\left[\begin{array}{c} y\\ c\\ a\\ e \end{array}\right]=\left[\begin{array}{cccc} V & \text{ZC} & \text{ZG} & R\\ CZ^{\prime } & C & 0 & 0\\ GZ^{\prime } & 0 & G & 0\\ R & 0 & 0 & R \end{array}\right] \end{array}$$

The phenotypic covariance matrix was:

$$V=Z_{1}CZ_{1}^{\prime }+Z_{2}GZ_{2}^{\prime }+R$$

For each trait student’s *t* tests [90] were performed to determine whether additive genetic variance was significantly different from zero (P < 0.05). The significance of the fixed effects (replicate and sex) were also tested for each trait with F-tests (P < 0.05). The coefficient of additive genetic variance **(*C*** **V**_**A**_**)** was calculated for each trait as:

$$CV_{A}=\frac{\sqrt{a}}{\bar{x}}$$

Where $\bar{x}$ is the phenotypic mean of the trait.

To examine the relationships between hop cone chemistry, yield and plant growth, residual maximum likelihood estimates of genetic correlation and phenotypic correlation between traits were calculated. In these bivariate analyses **y**, **c**, **a**, and **e** consist of vectors containing observations for two traits such that:

$$\begin{array}{l} y=\left(y_{1}^{\prime },y_{2}^{\prime }\right),\\ c=\left(c_{1}^{\prime },c_{2}^{\prime }\right),\\ a=\left(a_{1}^{\prime },a_{2}^{\prime }\right),\\ e=\left(e_{1}^{\prime },e_{2}^{\prime }\right),\\ X=X_{1}⊕X_{2},\\ Z_{1}=Z_{1_{1}}⊕Z_{1_{2}},\\ Z_{2}=Z_{2_{1}}⊕Z_{2_{2}},\\ C=I_{c}⊗C_{ο},\\ R=I_{N}⊗R_{o}\;\text{and}\\ G=A⊗G_{o} \end{array}$$

The variance-covariance matrices for the random effects (family and the replicate.incomplete-block term), additive genetic effects and residuals were represented by **C**_**o**_, **G**_**o**_ and **R**_**o**_ respectively:

$$\begin{array}{ll} \;C_{o} & =\left[\begin{array}{cc} \sigma_{c_{1}}^{2} & \sigma_{c_{12}}\\ \sigma_{c_{12}} & \sigma_{c_{2}}^{2} \end{array}\right],\;G_{o}=\left[\begin{array}{cc} \sigma_{a_{1}}^{2} & \sigma_{a_{12}}\\ \sigma_{a_{12}} & \sigma_{e_{2}}^{2} \end{array}\right]\\ & \;\text{and}\;R_{o}=\left[\begin{array}{cc} \sigma_{e_{1}}^{2} & \sigma_{e_{12}}\\ \sigma_{e_{12}} & \sigma_{e_{2}}^{2} \end{array}\right] \end{array}$$

The genetic and phenotypic relationships between plant growth and cone chemistry were investigated using the emergence traits number of shoots and length of the longest shoot (both measured in the first season, 11 months after the trial was planted); the vegetative morphology traits height (at flower initiation), height (at cone maturity), height to the cones and lateral length (all measured in the second season, 28 months after the trial was planted, except for height (at flower initiation) which was measured 25 months after the trial was planted); and cone chemical traits α-acid, β-acid, cohumulone (% of α-acid), α-acid:β-acid and α-acid:total resin (all measured in the second season, 28 months after the trial was planted). The genetic and phenotypic relationships between yield and plant growth and yield and hop chemistry were investigated using the yield trait green cone weight (measured in the second season, 28 months after the trial was planted) and the emergence, vegetative morphology and cone chemical traits listed above. The consistency of measurements of each individual trait used in these bivariate analyses was assessed by examining the genetic and phenotypic correlations between the results obtained from seasons one and two. Relationships between the different chemical traits were evaluated by investigating the genetic and phenotypic correlations between each chemical trait and every other chemical trait, with measurements from both seasons assessed. Genetic correlations between chemical components (α-acid and β-acid) and ratios between components (α-acid:β-acid and α-acid:total resin) were examined to determine whether the genetic factors influencing the amounts of chemical components also influenced the proportions of these components relative to each other and total resin content. Relationships between the different plant growth traits were also evaluated through the examination of genetic correlations. The significance of each genetic and phenotypic correlation were tested with student’s *t* tests (P < 0.05) [90].

Narrow-sense heritability **(*h***^***2***^**)** was calculated for each trait. This was computed in ASReml as:

$$h^{2}=\frac{\sigma_{a}^{2}}{\sigma_{a}^{2}+\sigma_{e}^{2}}$$

Least squares mean for each family were computed. These were estimated for every trait from the PREDICT statement in ASReml. Where necessary the values were backtransformed; and for each trait the upper 95% and lower 95% limits were calculated.

## Competing interests

The authors declare that they have no competing interests.

## Authors’ contributions

EM provided the phenotypic data for the growth traits, performed data analyses, interpreted the data and wrote the manuscript. SW was involved in the conception of the study, provided the phenotypic data of the cone chemical traits and the yield trait, assisted with data analyses and interpretations and revised the manuscript. RV assisted with data interpretations and revised the manuscript. AK was involved in the conception of the study. All authors read and approved the final manuscript.

## Supplementary Material

Additional file 1 **Least squares means for each family included in the hop quantitative genetic analysis.** Least squares means (lower 95% limit, upper 95% limit) for each family included in the quantitative genetic analysis, for traits relating to plant growth, yield and cone chemistry in hop. '*’ indicates traits for which the means have been backtransformed. **a.** refers to the traits number of shoots, length of the longest shoot and number of nodes on the longest shoot, relating to the emergence stage of plant growth. **b.** refers to the trait height, assessed at three different time points of the growing season (flower initiation, mid-season and cone maturity). **c.** refers to the traits lateral length, number of nodes on lateral and internode length, relating to the cone maturity stage of plant growth. **d.** refers to the trait height to the cones, relating to the cone maturity stage of plant growth; and green cone weight, relating to cone yield. **e.** refers to the cone chemical traits cohumulone, humulone + adhumulone, colupulone and lupulone + adlupulone. **f.** refers to the cone chemical traits α-acid and β-acid. **g.** refers to the cone chemical traits cohumulone (% of α-acid), α-acid:β-acid and α-acid:total resin. All traits were assessed in two seasons of plant growth, except for green cone weight, which was assessed in only the second season. Click here for file

Additional file 2 **Comparisons between quantitative genetic parameters calculated for hop in this study and previous studies.** Estimates of quantitative genetic parameters of hop cone chemical traits and yield calculated in this study are compared to the results of previous studies. **a.** refers to the number of families and estimates of narrow-sense heritability calculated for cone chemical traits and yield. The values reported from this study are averages of data from two seasons for cone chemical traits and one season for yield. **b.** refers to additive genetic correlations between the traits α-acid, β-acid and cone yield from previous studies of hop, compared to the values determined in this study. Correlations statistically different to zero (*P* < 0.05) are shown in bold. Click here for file

Additional file 3 **The female parents of a progeny trial used to investigate quantitative genetic variation in hop.** 'Hop accession’ refers to the cultivar name or accession number of the hop accessions used as a female parent. 'Origin’ refers to the country where the hop accessions were produced. Click here for file

## References

[B1] Falconer DS Mackay TFC Introduction to quantitative genetics 1996 Harlow, UK: Longman

[B2] Lynch M Walsh B Genetics and analysis of quantitative traits 1998 Sunderland: Sinauer Associates Inc.

[B3] Dudley JW Moll RH Interpretation and use of estimates of heritability and genetic variances in plant breeding Crop Sci 1969 9 257 262 10.2135/cropsci1969.0011183X000900030001x

[B4] Parker JS Clark MS Dosage sex-chromosome systems in plants Plant Sci 1991 80 79 92 10.1016/0168-9452(91)90274-C

[B5] Shephard HL Parker JS Darby P Ainsworth CC Sexual development and sex chromosomes in hop New Phytol 2000 148 397 411 10.1046/j.1469-8137.2000.00771.x 33863027

[B6] De Keukeleire D Fundamentals of beer and hop chemistry Quim Nova 2000 23 108 112 10.1590/S0100-40422000000100019

[B7] Oliveira MM Pais MS Glandular trichomes of *Humulus lupulus* var. Brewer’s gold (hops): ultrastructural aspects of peltate trichomes J Submicrosc Cytol Pathol 1990 22 241 248

[B8] Menary RD Doe PE Some morphological and chemical changes in hops during maturation J Sci Food Agric 1983 34 921 929 10.1002/jsfa.2740340905

[B9] Neve RA Hops 1991 London, UK: Chapman and Hall

[B10] Benitez JL Forster A De Keukeleire D Moir M Sharpe FR Verhagen LC Westwood KT Hops and hop products 1997 Nurenberg, Germany: Hans Carl-Verlag 975

[B11] Van Cleemput M Cattoor K De Bosscher K Haegeman G De Keukeleire D Heyerick A Hop (*Humulus lupulus*)-derived bitter acids as multipotent bioactive compounds J Nat Prod 2009 72 1220 1230 10.1021/np800740m 19476340

[B12] Shimwell JL On the relation between staining properties of bacteria and their reaction towards hops antiseptic J Inst Brew 1937 43 111 118 10.1002/j.2050-0416.1937.tb05727.x

[B13] Hough JS Howard GA Slater CA Bacteriostatic activities of hop resin materials J Inst Brew 1957 63 331 333 10.1002/j.2050-0416.1957.tb06267.x

[B14] Michener HD Snell N Jansen EF Antifungal activity of hop resin constituents and a new method for isolation of lupulon Arch Biochem Biophys 1948 19 199 208 18891772

[B15] Eri S Khoo BK Lech J Hartman TG Direct thermal desorption-gas chromatography and gas chromatography-mass spectrometry profiling of hop (*Humulus lupulus* L.) essential oils in support of varietal characterization J Agric Food Chem 2000 48 1140 1149 10.1021/jf9911850 10775363

[B16] Roberts MT Dufour JP Lewis AC Application of comprehensive multidimensional gas chromatography combined with time-of-flight mass spectrometry (GC x GC-TOFMS) for high resolution analysis of hop essential oil J Sep Sci 2004 27 473 478 10.1002/jssc.200301669 15335083

[B17] Čeh B Kač M Košir IJ Abram V Relationships between xanthohumol and polyphenol content in hop leaves and hop cones with regard to water supply and cultivar Int J Mol Sci 2007 8 989 1000 10.3390/i8090989

[B18] Sharpe FR Laws RJ The essential oil of hops a review J Inst Brew 1981 87 96 107 10.1002/j.2050-0416.1981.tb03996.x

[B19] Pavolovic V Cerenak A Pavolovic M Kosir IJ Rozman C Bohanec M Ceh B Naglic B Modelling of quality prediction for hops (*Humulus lupulus* L.) in relation to meteorological variables BALWOIS conference proceedings; Ohrid, Republic of Macedonia 2010 1 10

[B20] Thomas GG Schwabe WW Factors controlling flowering in the hop (*Humulus lupulus* L.) Ann Bot 1969 33 781 793

[B21] Delyser DY Kasper WJ Hopped beer: the case for cultivation Econ Bot 1994 48 166 170 10.1007/BF02908210

[B22] Small E A numerical and nomenclatural analysis of morpho-geographic taxa of *Humulus* Syst Bot 1978 3 37 76 10.2307/2418532

[B23] Moir M Hops - a millennium review J Am Soc Brew Chem 2000 58 131 146

[B24] Henning JA Townsend MS Field-based estimates of heritability and genetic correlations in hop Crop Sci 2005 45 1469 1475 10.2135/cropsci2004.0360

[B25] Henning J Haunold A Nickerson G Genetic parameter estimates for five traits in male hop accesssions: a preliminary study J Am Soc Brew Chem 1997 55 157 160

[B26] Henning J Haunold A Nickerson G Gampert U Estimates of heritability and genetic correlation for five traits in female hop accessions J Am Soc Brew Chem 1997 55 161 165

[B27] Beatson RA Alspach PA The use of empirical breeding values to improve genetic progress in hops Acta Hortic (ISHS) 2008 848 93 100

[B28] Keller KR Likens ST Estimates of heritability in hop, *Humulus lupulus* L Agron J 1955 47 518 512 10.2134/agronj1955.00021962004700110007x

[B29] Roberts DD Kronstad WE Haunold A Genetic variability and association of maturity, yield, and quality characteristics of female hops Crop Sci 1980 20 523 527 10.2135/cropsci1980.0011183X002000040026x

[B30] Kralj D Haunold A The breeding potential of native hops (*Humulus lupulus* L.) from Yugoslavia Monatsschrift Brauwiss 1987 40 287 293

[B31] Murakami A Inheritance of major chemical components in hops J Inst Brew 1999 105 107 111 10.1002/j.2050-0416.1999.tb00013.x

[B32] Hautke P Petřiček D Inheritance of biochemical characteristics in hops - report of a brief study Wallerstein Comm 1967 30 135 138

[B33] Farrar RF Neve RA Weston EW Inheritance of resin characters in hops Wye College Department of Hop Research Annual Report 1955 Kent, England, Wye College 52 59

[B34] Nesvadba V Černy J Krofta K Transfer fo the hop (*Humulus lupulus* L.) alpha-bitter acid content to progenies of F_1_ and I_1_ generations in selected parental components Plant Soil Environ 2003 49 269 276

[B35] Brooks SN Effectiveness of selection within Fuggle hops (*Humulus lupulus* L.) Crop Sci 1962 2 5 10 10.2135/cropsci1962.0011183X000200010002x

[B36] Versluys JP Choice of male and female parents of seedling populations of hops in relation to the alpha-acid content and the time fo ripening in the female progeny J Inst Brew 1969 75 25 26

[B37] Likens ST Nickerson GB Haunold A Zimmermann CE Relationship between alpha acids, beta acids and lupulin content of hops Crop Sci 1978 17 380 386

[B38] Brooks SN Associaton of quality characters in flowers of male hops Crop Sci 1962 2 192 196 10.2135/cropsci1962.0011183X000200030004x

[B39] Brooks SN Likens ST Variability of morphological and chemical quality characters in flowers of male hops Crop Sci 1962 2 189 196 10.2135/cropsci1962.0011183X000200030003x

[B40] Hill WG Understanding and using quantitative genetic variation Phil Trans R Soc B 2010 365 73 85 10.1098/rstb.2009.0203 20008387 PMC2842708

[B41] Perron M DeBlois J Desponts M Use of resampling to assess optimal subgroup composition for estimating genetic parameters from progeny trials Tree Genet Genomes 2013 9 129 143 10.1007/s11295-012-0540-5

[B42] McAdam E Freeman J Whittock S Buck E Jakse J Cerenak A Javornik B Kilian A Wang C-H Andersen D Vaillancourt R Carling J Beatson R Graham L Graham D Darby P Koutoulis A Quantitative trait loci in hop (*Humulus lupulus* L.) reveal complex genetic architecture underlying variation in sex, yield and cone chemistry BMC Genomics 2013 14 360 10.1186/1471-2164-14-360 23718194 PMC3680207

[B43] Koie K Inaba A Okada I Kaneko T Ito K Construction of the genetic linkage map and QTL analysis on hop (*Humulus lupulus* L.) Acta Hortic (ISHS) 2005 668 59 66

[B44] Cerenak A Satovic Z Jakse J Luthar Z Carovic-Stanko K Javornik B Identification of QTLs for alpha acid content and yield in hop (*Humulus lupulus* L.) Euphytica 2009 170 141 154 10.1007/s10681-009-9920-9

[B45] Henning JA Townsend MS Gent DH Bassil N Matthews P Buck E Beatson R QTL mapping of powdery mildew susceptibility in hop (*Humulus lupulus* L.) Euphytica 2011 180 411 420 10.1007/s10681-011-0403-4

[B46] Patzak J Henychova A Krofta K Nesvadba V Study of molecular markers for xanthohumol and DMX contents in hop (*Humulus lupulus* L.) by QTLs mapping analysis Brew Sci 2012 65 96 102

[B47] Seefelder S Lutz A Seigner E Development of molecular markers for powdery mildew resistance to support breeding for high quality hops Monatsschrift Brauwiss 2006 59 100 104

[B48] O’Reilly-Wapstra JM McArthur C Potts BM Linking plant genotype, plant defensive chemistry and mammal browsing in a Eucalyptus species Funct Ecol 2004 18 677 684 10.1111/j.0269-8463.2004.00887.x

[B49] Moore BD Foley WJ Tree use by koalas in a chemically complex landscape Nature 2005 435 488 490 10.1038/nature03551 15917807

[B50] Jakše J Kindlhofer K Javornik B Assessment of genetic variation and differentiation of hop genotypes by microsatellite and AFLP markers Genome 2001 44 773 782 10.1139/gen-44-5-773 11681600

[B51] Murakami A Darby P Javornik B Pais MSS Seigner E Lutz A Svoboda P Molecular phylogeny of wild hops, *Humulus lupulus* L Heredity 2006 97 66 74 10.1038/sj.hdy.6800839 16685279

[B52] Murphey JM Probasco G The development of brewing quality characteristics in hops during maturation Tech Q Master Brew Assoc Am 1996 33 149 159

[B53] Bassil NV Gilmore B Oliphant JM Hummer KE Henning JA Genic SSRs for European and North American hop (*Humulus lupulus* L.) Genet Resour Crop Evol 2008 55 959 969 10.1007/s10722-007-9303-9

[B54] Henning JA Steiner JJ Hummer KE Genetic diversity among world hop accessions grown in the USA Crop Sci 2004 44 411 417 10.2135/cropsci2004.0411

[B55] Jakše J Satovic Z Javornik B Microsatellite variability among wild and cultivated hops (*Humulus lupulus* L.) Genome 2004 47 889 899 10.1139/g04-054 15499403

[B56] Murakami A Darby P Javornik B Pais MSS Seigner E Lutz A Svoboda P Microsatellite DNA analysis of wild hops, *Humulus lupulus* L Genet Resour Crop Evol 2006 53 1553 1562 10.1007/s10722-005-7765-1

[B57] Patzak J Nesvadba V Krofta K Henychová A Inalovic M Richards K Evaluation of genetic variability of wild hops (*Humulus lupulus* L.) in Canada and Caucasus region by chemical and molecular methods Genome 2010 53 545 557 10.1139/G10-024 20616876

[B58] Peredo EL Revilla MÁ Reed BM Javornik B Cires E Prieto JAF Arroyo-García R The influence of European and American wild germplasm in hop (*Humulus lupulus* L.) cultivars Genet Resour Crop Evol 2010 57 575 586 10.1007/s10722-009-9495-2

[B59] Stajner N Satovic Z Cerenak A Javornik B Genetic structure and differentiation in hop (*Humulus lupulus* L.) as inferred from microsatellites Euphytica 2008 161 301 311 10.1007/s10681-007-9429-z

[B60] Šuštar-Vozlič J Javornik B Genetic relationships in cultivars of hop, *Humulus lupulus* L., determined by RAPD analysis Plant Breed 1999 118 175 181 10.1046/j.1439-0523.1999.118002175.x

[B61] Howard EL Whittock SP Jakše J Carling J Matthews PD Probasco G Henning JA Darby P Cerenak A Javornik B Kilian A Koutoulis A High-throughput genotyping of hop (*Humulus lupulus* L.) utilising diversity arrays technology (DArT) Theor Appl Genet 2011 122 1265 1280 10.1007/s00122-011-1529-4 21243330

[B62] Steane DA Conod N Jones RC Vaillancourt RE Potts BM A comparative analysis of population structure of a forest tree, *Eucalyptus globulus* (Myrtaceae), using microsatellite markers and quantitative traits Tree Genet Genomes 2006 2 30 38 10.1007/s11295-005-0028-7

[B63] Dawson T Geber M Sexual dimorphism in physiology and morphology Gender and sexual dimorphism in flowering plants 1999 New York: Springer-Verlag Berlin Heidelberg 175 210

[B64] Dzhaparidze L Sex in plants, part II. Biochemical and physiological sex differences in dioecious plants: problem of influencing sex formation 1969 Jerusalem: IPST Press

[B65] Charlesworth D Willis JH The genetics of inbreeding depression Nat Rev Genet 2009 10 783 796 10.1038/nrg2664 19834483

[B66] Duvick DN Biotechnology in the 1930s: the development of hybrid maize Nat Rev Genet 2001 2 69 74 10.1038/35047587 11253074

[B67] Patzak J Nesvadba V Henychová A Krofta K Assessment of the genetic diversity of wild hops (*Humulus lupulus* L.) in Europe using chemical and molecular analyses Biochem Syst Ecol 2010 38 136 145 10.1016/j.bse.2009.12.023

[B68] Krofta K Comparison of quality parameters of Czech and foreign hop varieties Plant Soil Environ 2003 49 261 268

[B69] Rigby FL A theory on the hop flavour of beer Proc Am Soc Brew Chem 1972 46 46 50

[B70] Meilgaard M Hop analysis, cohumulone factor and the bitterness of beer: review and critical evaluation J Inst Brew 1960 66 35 50 10.1002/j.2050-0416.1960.tb01696.x

[B71] Wackerbauer K Balzer U Hop bitter compounds in beer, part II: the influence of cohumulone on beer quality BRAUWELT Int 1993 11 116 118

[B72] Kollmannsberger H Biendl M Nitz S Occurence of glycosidically bound flavour compounds in hops, hop products and beer Monatsschrift Brauwiss 2006 5 83 89

[B73] Takoi K Koie K Itoga Y Katayama Y Shimase M Nakayama Y Watari J Biotransformation of hop-derived monoterpene alcohols by lager yeast and their contribution to the flavor of hopped beer J Agric Food Chem 2010 58 5050 5058 10.1021/jf1000524 20364865

[B74] Lande R Arnold SJ The measurement of selection on correlated characters Evolution 1983 37 1210 1226 10.2307/2408842 28556011

[B75] Patterson HD Williams ER A new class of resolvable incomplete block designs Biometrika 1976 63 83 92 10.1093/biomet/63.1.83

[B76] Roborgh RHJ Classification and botanical description of imported varieties of hops in Nelson, New Zealand New Zeal J Bot 1964 2 10 18 10.1080/0028825X.1964.10428714

[B77] Homer CE Brooks SN Haunold A Likens ST Registration of hop germplasm (Reg. No. GP 2 to 4) Crop Sci 1974 14 341 341

[B78] Zimmermann CE Likens ST Haunold A Horner CE Roberts DD Registration of Comet hop (Reg. No. 3) Crop Sci 1975 15 98 98

[B79] Haunold A Likens ST Nickerson GB Horner CE Hampton RO Registration of USDA 63015 M male hop germplasm (Reg. No. GP 14) Crop Sci 1983 23 600 601 10.2135/cropsci1983.0011183X002300030047x

[B80] Neve RA Darby P Yeoman and Zenith: two new varieties Wye College Department of Hop Research Annual Report 1984 Kent, England, Wye College 39 44

[B81] Kenny ST Zimmermann CE Registration of 'Chinook’ hop Crop Sci 1986 26 196 197

[B82] Miyata J Qualities and ancestry of prominent hop varieties Brewer Distiller Int 1994 24 27

[B83] Patzak J Characterization of Czech hop (*Humulus lupulus* L.) genotypes by molecular methods Rost Výroba 2002 48 343 350

[B84] Reed BM *In vitro* storage of hop (*Humulus* L.) germplasm Acta Hortic (ISHS) 2005 668 249 256

[B85] Simply Hops 2012 http://www.simplyhops.co.uk

[B86] Oregon State University High Alpha Acid Breeding Program Freshops USDA named hop variety descriptions 2012 http://www.freshops.com/hops/usda-named-hop-variety-descriptions

[B87] USDA ARS Germplasm Resources Information Network (GRIN) 2012 Beltsville, Maryland: National Germplasm Resources Laboratory http://www.ars-grin.gov/cgi-bin/npgs/html/index.pl

[B88] Gilmour AR Gogel BJ Cullis BR Thompson R ASReml user guide release 3 2009 Hemel Hempstead, HP1 1ES, UK: VSN International LtdVSN International Ltd

[B89] Gilmour AR ASREML for testing fixed effects and estimating multiple trait variance components Proc Adv Anim Breed Gen 1997 12 386 390

[B90] Gosset WS The probable error or a mean Biometrika 1908 VI 1 25 'Student’

