# Supplementary material for: Quantitative genetic parameters for yield, plant growth and cone chemical traits in hop (Humulus lupulus L.)
Source: BMC Genet. 2014 Feb 13;15:22. doi: 10.1186/1471-2156-15-22 (PMC3927220; doi:10.1186/1471-2156-15-22)
Supplement: Additional file 1 — Least squares means for each family included in the hop quantitative genetic analysis. Least squares means (lower 95% limit, upper 95% limit) for each family included in the quantitative genetic analysis, for traits relating to plant growth, yield and cone chemistry in hop. '*’ indicates traits for which the means have been backtransformed. a. refers to the traits number of shoots, length of the longest shoot and number of nodes on the longest shoot, relating to the emergence stage of plant growth. b. refers to the trait height, assessed at three different time points of the growing season (flower initiation, mid-season and cone maturity). c. refers to the traits lateral length, number of nodes on lateral and internode length, relating to the cone maturity stage of plant growth. d. refers to the trait height to the cones, relating to the cone maturity stage of plant growth; and green cone weight, relating to cone yield. e. refers to the cone chemical traits cohumulone, humulone + adhumulone, colupulone and lupulone + adlupulone. f. refers to the cone chemical traits α-acid and β-acid. g. refers to the cone chemical traits cohumulone (% of α-acid), α-acid:β-acid and α-acid:total resin. All traits were assessed in two seasons of plant growth, except for green cone weight, which was assessed in only the second season. [file 1471-2156-15-22-S1.pdf]

Additional file 1 Least squares means for each family included in the hop quantitative genetic analysis.

Least squares means (lower 95% limit, upper 95% limit) for each family included in the quantitative genetic analysis, for traits relating to plant growth, yield and cone chemistry in hop. '*' indicates traits for which the means have been backtransformed**.** **a.** refers to the traits number of shoots, length of the longest shoot and number of nodes on the longest shoot, relating to the emergence stage of plant growth. **b.** refers to the trait height, assessed at three different time points of the growing season (flower initiation, mid-season and cone maturity). **c.** refers to the traits lateral length, number of nodes on lateral and internode length, relating to the cone maturity stage of plant growth. **d.** refers to the trait height to the cones, relating to the cone maturity stage of plant growth; and green cone weight, relating to cone yield. **e.** refers to the cone chemical traits cohumulone, humulone + adhumulone, colupulone and lupulone + adlupulone. **f.** refers to the cone chemical traits α-acid and β-acid. **g.** refers to the cone chemical traits cohumulone (% of α-acid), α-acid:β-acid and α-acid:total resin. All traits were assessed in two seasons of plant growth, except for green cone weight, which was assessed in only the second season.

a.

**Family**	**number of shoots**		**number of shoots**		**length of the longest shoot**		**length of the longest shoot**		**number of nodes on the longest shoot**		**number of nodes on the longest shoot**	
	11 months *		24 months		11 months *		24 months *		11 months		24 months *	
2008003	3.71	(2.98 , 4.51)	7.46	(6.04 , 8.89)	42.60	(36.53 , 49.13)	8.10	(5.97 , 10.56)	6.80	(6.30 , 7.30)	2.69	(2.28 , 3.13)
2008190	4.44	(3.64 , 5.31)	8.17	(6.75 , 9.59)	44.93	(38.69 , 51.65)	8.94	(6.77 , 11.41)	7.26	(6.75 , 7.76)	2.68	(2.29 , 3.11)
2008209	4.46	(3.65 , 5.36)	6.75	(5.29 , 8.22)	47.64	(41.10 , 54.67)	10.18	(7.56 , 13.18)	6.93	(6.42 , 7.44)	2.80	(2.36 , 3.28)
2008151	4.12	(3.35 , 4.96)	5.67	(4.25 , 7.09)	32.71	(27.51 , 38.36)	8.40	(6.15 , 11.01)	6.01	(5.51 , 6.50)	2.77	(2.34 , 3.23)
2008039	4.88	(4.04 , 5.80)	8.92	(7.50 , 10.34)	47.77	(41.43 , 54.56)	9.04	(6.86 , 11.52)	7.62	(7.13 , 8.11)	2.72	(2.32 , 3.15)
2008152	3.76	(3.03 , 4.57)	5.72	(4.30 , 7.15)	48.41	(41.91 , 55.37)	8.51	(6.23 , 11.14)	7.28	(6.78 , 7.79)	2.82	(2.39 , 3.29)
2008001	4.87	(4.00 , 5.82)	8.19	(6.68 , 9.70)	49.10	(42.45 , 56.23)	7.54	(5.56 , 9.81)	7.93	(7.42 , 8.45)	2.62	(2.24 , 3.05)
2008073	3.36	(2.67 , 4.13)	5.68	(4.26 , 7.10)	40.51	(34.39 , 47.14)	5.38	(3.61 , 7.51)	6.63	(6.10 , 7.15)	2.34	(1.95 , 2.76)
2008168	4.55	(3.74 , 5.43)	10.96	(9.54 , 12.38)	48.27	(41.90 , 55.09)	18.45	(15.38 , 21.80)	7.24	(6.75 , 7.73)	3.40	(2.96 , 3.86)
2008161	4.32	(3.54 , 5.19)	7.86	(6.44 , 9.29)	45.66	(39.26 , 52.55)	7.39	(5.44 , 9.65)	6.69	(6.18 , 7.21)	2.92	(2.51 , 3.36)
2008290	3.57	(2.83 , 4.39)	5.52	(4.01 , 7.03)	41.10	(34.81 , 47.92)	6.48	(4.33 , 9.05)	7.10	(6.57 , 7.64)	2.40	(1.98 , 2.87)
2008208	3.72	(2.99 , 4.52)	6.35	(4.93 , 7.78)	38.16	(32.43 , 44.37)	10.07	(7.47 , 13.05)	6.66	(6.16 , 7.16)	2.74	(2.30 , 3.22)
2008251	4.30	(3.52 , 5.17)	9.02	(7.60 , 10.44)	40.93	(34.99 , 47.35)	7.25	(5.38 , 9.41)	7.16	(6.66 , 7.66)	2.65	(2.27 , 3.06)
2008052	5.83	(4.90 , 6.85)	10.93	(9.46 , 12.39)	54.08	(47.21 , 61.42)	13.46	(10.86 , 16.34)	7.34	(6.84 , 7.84)	3.08	(2.67 , 3.52)
2008037	4.34	(3.54 , 5.22)	4.28	(2.82 , 5.74)	37.99	(32.18 , 44.29)	5.59	(3.70 , 7.86)	6.95	(6.44 , 7.46)	2.10	(1.72 , 2.52)
2008154	4.42	(3.62 , 5.29)	9.19	(7.76 , 10.61)	45.27	(39.10 , 51.89)	10.47	(8.19 , 13.03)	7.13	(6.64 , 7.63)	2.93	(2.53 , 3.37)
2008136	5.90	(4.98 , 6.90)	10.80	(9.37 , 12.22)	53.42	(46.70 , 60.60)	14.87	(12.21 , 17.79)	7.79	(7.30 , 8.28)	3.61	(3.17 , 4.08)
2008096	4.52	(3.72 , 5.40)	9.69	(8.26 , 11.11)	44.13	(38.05 , 50.66)	15.55	(12.54 , 18.89)	7.40	(6.91 , 7.90)	3.31	(2.86 , 3.80)
2008071	3.87	(3.12 , 4.69)	6.18	(4.75 , 7.60)	38.52	(32.65 , 44.87)	5.66	(3.91 , 7.74)	6.58	(6.07 , 7.09)	2.05	(1.70 , 2.44)
2008124	5.20	(4.34 , 6.15)	10.72	(9.30 , 12.14)	51.69	(45.09 , 58.74)	13.43	(10.91 , 16.21)	7.79	(7.29 , 8.28)	2.90	(2.51 , 3.32)
2008145	5.67	(4.77 , 6.66)	7.86	(6.44 , 9.29)	45.99	(39.76 , 52.66)	8.54	(6.49 , 10.87)	7.35	(6.86 , 7.84)	2.98	(2.58 , 3.42)
2008084	5.14	(4.28 , 6.08)	7.05	(5.62 , 8.47)	45.31	(39.14 , 51.92)	6.13	(4.15 , 8.50)	7.09	(6.60 , 7.59)	2.14	(1.75 , 2.56)
2008083	5.14	(4.28 , 6.07)	8.63	(7.21 , 10.05)	48.29	(41.92 , 55.12)	11.26	(8.81 , 14.01)	7.66	(7.17 , 8.15)	3.14	(2.71 , 3.59)

Family	number of shoots<br>11 months *		number of shoots<br>24 months		length of the longest shoot<br>11 months *		length of the longest shoot<br>24 months *		number of nodes on the longest shoot<br>11 months		number of nodes on the longest shoot<br>24 months *	
2008056	4.17	(3.40 , 5.02)	6.11	(4.69 , 7.53)	41.81	(35.80 , 48.28)	8.12	(5.80 , 10.82)	6.55	(6.05 , 7.05)	2.97	(2.51 , 3.46)
2008261	3.80	(3.07 , 4.62)	5.62	(4.20 , 7.04)	43.02	(36.71 , 49.84)	6.08	(4.26 , 8.23)	7.19	(6.66 , 7.71)	2.21	(1.84 , 2.60)
2008157	4.91	(4.07 , 5.82)	8.78	(7.36 , 10.20)	49.14	(42.72 , 56.02)	6.25	(4.57 , 8.18)	7.10	(6.61 , 7.59)	2.64	(2.27 , 3.04)
2008006	4.92	(4.08 , 5.83)	10.98	(9.56 , 12.40)	53.38	(46.67 , 60.54)	15.78	(13.04 , 18.79)	7.58	(7.08 , 8.07)	3.78	(3.33 , 4.25)
2008240	5.37	(4.49 , 6.33)	8.13	(6.71 , 9.55)	50.69	(44.16 , 57.68)	10.33	(8.07 , 12.87)	7.29	(6.80 , 7.78)	2.75	(2.36 , 3.17)
2008103	4.57	(3.76 , 5.46)	8.56	(7.14 , 9.98)	45.20	(38.94 , 51.94)	12.20	(9.55 , 15.18)	7.24	(6.73 , 7.74)	3.27	(2.82 , 3.75)
2008148	4.99	(4.14 , 5.91)	8.13	(6.70 , 9.55)	47.20	(40.89 , 53.96)	7.79	(5.84 , 10.02)	7.32	(6.83 , 7.81)	2.49	(2.12 , 2.89)
2008236	4.83	(4.00 , 5.74)	7.35	(5.93 , 8.78)	51.08	(44.51 , 58.09)	8.14	(6.00 , 10.60)	7.39	(6.89 , 7.88)	2.55	(2.16 , 2.98)
2008259	5.84	(4.92 , 6.84)	11.30	(9.88 , 12.72)	56.34	(49.33 , 63.82)	15.04	(12.28 , 18.08)	7.70	(7.20 , 8.20)	3.45	(3.02 , 3.92)
2008191	4.79	(3.94 , 5.72)	7.95	(6.48 , 9.41)	44.62	(38.39 , 51.32)	6.48	(4.58 , 8.70)	6.78	(6.27 , 7.28)	2.28	(1.90 , 2.68)
2008242	5.10	(4.24 , 6.04)	7.27	(5.85 , 8.70)	49.69	(43.10 , 56.74)	8.28	(6.26 , 10.57)	7.42	(6.91 , 7.92)	2.41	(2.05 , 2.81)
2008248	4.29	(3.50 , 5.17)	7.84	(6.37 , 9.30)	45.79	(39.38 , 52.69)	9.20	(7.00 , 11.70)	6.84	(6.32 , 7.35)	2.95	(2.54 , 3.40)
2008010	4.16	(3.39 , 5.01)	11.34	(9.91 , 12.76)	46.16	(39.82 , 52.96)	14.77	(12.12 , 17.68)	7.16	(6.66 , 7.66)	3.39	(2.97 , 3.85)
2008263	4.55	(3.74 , 5.44)	8.57	(7.15 , 9.99)	40.04	(34.16 , 46.39)	10.80	(8.40 , 13.49)	6.75	(6.25 , 7.26)	3.03	(2.61 , 3.48)
2008153	4.78	(3.95 , 5.69)	7.47	(6.05 , 8.90)	48.11	(41.52 , 55.18)	9.39	(7.23 , 11.83)	7.22	(6.70 , 7.73)	2.82	(2.42 , 3.24)
2008119	4.67	(3.85 , 5.57)	6.55	(5.12 , 7.97)	42.45	(36.39 , 48.99)	10.54	(7.88 , 13.59)	7.19	(6.69 , 7.70)	2.75	(2.31 , 3.22)
2008206	3.94	(3.19 , 4.77)	8.04	(6.62 , 9.47)	41.64	(35.63 , 48.11)	7.79	(5.84 , 10.03)	6.96	(6.46 , 7.47)	2.58	(2.20 , 2.98)
2008139	3.83	(3.09 , 4.64)	5.22	(3.80 , 6.65)	43.25	(36.90 , 50.11)	5.68	(3.86 , 7.85)	6.78	(6.25 , 7.31)	2.27	(1.89 , 2.69)
2008106	4.85	(4.02 , 5.76)	11.24	(9.82 , 12.66)	52.61	(45.83 , 59.84)	17.39	(14.41 , 20.64)	7.79	(7.29 , 8.30)	3.85	(3.39 , 4.35)
2008122	4.44	(3.63 , 5.33)	8.34	(6.88 , 9.80)	50.86	(44.20 , 57.97)	12.51	(9.92 , 15.40)	7.24	(6.74 , 7.74)	3.30	(2.86 , 3.77)
2008155	5.01	(4.16 , 5.93)	7.46	(6.04 , 8.88)	46.80	(40.53 , 53.53)	9.16	(6.89 , 11.75)	7.17	(6.68 , 7.66)	2.69	(2.28 , 3.12)
2008088	4.62	(3.81 , 5.51)	10.42	(9.00 , 11.84)	45.09	(38.94 , 51.70)	10.03	(7.80 , 12.53)	7.48	(6.98 , 7.97)	3.08	(2.67 , 3.52)
2008111	5.24	(4.37 , 6.19)	7.65	(6.23 , 9.07)	41.61	(35.72 , 47.96)	9.73	(7.45 , 12.31)	7.03	(6.54 , 7.53)	2.83	(2.42 , 3.27)
2008202	4.49	(3.68 , 5.39)	5.88	(4.42 , 7.34)	50.82	(44.16 , 57.94)	7.75	(5.60 , 10.26)	7.74	(7.24 , 8.24)	2.63	(2.22 , 3.08)
2008013	4.22	(3.45 , 5.08)	6.44	(5.02 , 7.86)	42.30	(36.36 , 48.70)	8.60	(6.32 , 11.23)	6.50	(6.01 , 6.99)	2.55	(2.14 , 2.99)
2008279	4.88	(4.04 , 5.79)	8.09	(6.67 , 9.51)	47.95	(41.39 , 55.00)	9.27	(7.06 , 11.78)	7.48	(6.97 , 7.99)	2.76	(2.36 , 3.19)
2008007	4.01	(3.25 , 4.84)	6.76	(5.34 , 8.18)	45.43	(39.15 , 52.17)	8.96	(6.72 , 11.53)	7.08	(6.58 , 7.58)	2.61	(2.21 , 3.04)
2008090	6.13	(5.19 , 7.15)	10.14	(8.72 , 11.56)	49.15	(42.71 , 56.04)	13.97	(11.32 , 16.91)	7.93	(7.44 , 8.42)	3.29	(2.86 , 3.74)
2008055	4.33	(3.54 , 5.19)	7.63	(6.21 , 9.05)	46.15	(39.92 , 52.84)	7.90	(5.87 , 10.24)	6.77	(6.28 , 7.27)	2.38	(2.01 , 2.79)
2008271	3.74	(3.02 , 4.55)	6.58	(5.16 , 8.00)	47.14	(40.63 , 54.14)	9.61	(7.28 , 12.26)	7.26	(6.75 , 7.78)	2.96	(2.54 , 3.42)
2008035	5.00	(4.15 , 5.92)	9.04	(7.62 , 10.46)	47.53	(41.11 , 54.42)	9.06	(6.95 , 11.45)	7.56	(7.06 , 8.06)	2.97	(2.56 , 3.40)
2008212	3.79	(3.06 , 4.61)	8.19	(6.77 , 9.61)	38.59	(32.82 , 44.83)	8.78	(6.64 , 11.22)	6.76	(6.26 , 7.27)	2.57	(2.19 , 2.99)
2008149	5.90	(4.98 , 6.90)	7.34	(5.92 , 8.76)	48.86	(42.45 , 55.72)	8.92	(6.68 , 11.48)	7.82	(7.32 , 8.31)	2.78	(2.37 , 3.22)
2008170	5.50	(4.61 , 6.47)	8.08	(6.66 , 9.50)	49.04	(42.62 , 55.92)	9.30	(7.23 , 11.64)	7.47	(6.98 , 7.97)	2.62	(2.25 , 3.02)
2008247	3.88	(3.14 , 4.70)	6.32	(4.90 , 7.74)	45.65	(39.35 , 52.41)	7.14	(5.22 , 9.36)	7.07	(6.56 , 7.57)	2.38	(2.01 , 2.78)
2008110	4.12	(3.34 , 4.97)	6.60	(5.13 , 8.06)	42.46	(36.30 , 49.11)	6.87	(4.99 , 9.04)	6.83	(6.32 , 7.34)	2.03	(1.69 , 2.41)

Family	number of shoots<br>11 months *		number of shoots<br>24 months		length of the longest shoot<br>11 months *		length of the longest shoot<br>24 months *		number of nodes on the longest shoot<br>11 months		number of nodes on the longest shoot<br>24 months *	
2008043	3.34	(2.65 , 4.10)	6.51	(5.09 , 7.94)	41.99	(35.85 , 48.62)	6.72	(4.79 , 8.97)	6.76	(6.25 , 7.28)	2.43	(2.04 , 2.85)
2008188	4.11	(3.35 , 4.95)	6.76	(5.34 , 8.18)	48.08	(41.62 , 55.01)	15.98	(12.80 , 19.52)	7.01	(6.51 , 7.51)	2.94	(2.50 , 3.41)
2008080	4.75	(3.89 , 5.69)	6.80	(5.29 , 8.31)	43.43	(37.08 , 50.27)	6.81	(4.88 , 9.07)	6.75	(6.23 , 7.27)	2.50	(2.11 , 2.93)
2008239	3.46	(2.77 , 4.24)	6.76	(5.34 , 8.18)	48.38	(41.79 , 55.46)	6.31	(4.57 , 8.32)	7.11	(6.59 , 7.62)	2.36	(2.00 , 2.74)
2008075	4.62	(3.81 , 5.51)	9.55	(8.12 , 10.97)	46.55	(40.29 , 53.27)	13.03	(10.55 , 15.78)	7.43	(6.94 , 7.92)	2.66	(2.28 , 3.06)
2008114	3.93	(3.15 , 4.79)	6.42	(4.91 , 7.93)	35.58	(29.95 , 41.69)	7.09	(5.03 , 9.50)	6.29	(5.77 , 6.80)	2.31	(1.93 , 2.74)
2008166	5.26	(4.39 , 6.21)	10.07	(8.65 , 11.49)	41.17	(35.31 , 47.49)	10.74	(8.35 , 13.43)	6.80	(6.31 , 7.29)	3.21	(2.77 , 3.67)
2008252	5.02	(4.17 , 5.95)	8.62	(7.19 , 10.04)	49.60	(43.14 , 56.52)	10.00	(7.77 , 12.51)	7.55	(7.06 , 8.04)	2.62	(2.24 , 3.03)
2008150	4.40	(3.61 , 5.27)	5.55	(4.12 , 6.97)	39.32	(33.49 , 45.61)	6.30	(4.44 , 8.48)	6.90	(6.40 , 7.41)	2.51	(2.12 , 2.94)
2008009	4.27	(3.49 , 5.13)	7.22	(5.80 , 8.65)	43.89	(37.72 , 50.53)	8.99	(6.74 , 11.56)	6.98	(6.47 , 7.48)	2.51	(2.11 , 2.93)
2008081	3.61	(2.87 , 4.43)	8.61	(7.10 , 10.12)	44.69	(38.24 , 51.65)	10.59	(8.13 , 13.38)	6.98	(6.46 , 7.51)	3.10	(2.66 , 3.57)
2008041	4.79	(3.96 , 5.70)	8.78	(7.36 , 10.21)	46.32	(40.07 , 53.02)	9.09	(6.90 , 11.58)	7.67	(7.18 , 8.16)	2.87	(2.46 , 3.31)
2008061	5.40	(4.50 , 6.38)	8.98	(7.52 , 10.44)	47.55	(41.12 , 54.45)	9.51	(7.27 , 12.05)	7.13	(6.63 , 7.63)	3.01	(2.59 , 3.46)
2008087	3.70	(2.98 , 4.50)	8.11	(6.69 , 9.53)	44.84	(38.61 , 51.54)	9.21	(7.01 , 11.71)	7.18	(6.68 , 7.69)	3.01	(2.59 , 3.46)
2008147	4.53	(3.72 , 5.41)	9.22	(7.79 , 10.64)	44.49	(38.28 , 51.18)	12.65	(10.20 , 15.36)	7.28	(6.78 , 7.78)	3.09	(2.69 , 3.53)
2008258	4.50	(3.70 , 5.38)	9.32	(7.90 , 10.74)	49.60	(42.92 , 56.76)	9.10	(6.91 , 11.59)	7.53	(7.02 , 8.04)	2.81	(2.41 , 3.25)
2008130	4.20	(3.43 , 5.05)	6.45	(5.03 , 7.87)	41.98	(35.84 , 48.61)	9.17	(6.81 , 11.88)	6.98	(6.46 , 7.49)	2.85	(2.42 , 3.31)
2008100	4.17	(3.38 , 5.03)	6.64	(5.18 , 8.10)	40.29	(34.40 , 46.66)	12.89	(10.26 , 15.82)	6.54	(6.04 , 7.05)	2.91	(2.50 , 3.36)
2008132	5.03	(4.18 , 5.96)	10.03	(8.61 , 11.45)	48.35	(41.98 , 55.17)	13.24	(10.66 , 16.09)	7.62	(7.13 , 8.11)	3.14	(2.72 , 3.58)
2008256	5.73	(4.82 , 6.72)	11.04	(9.62 , 12.46)	48.50	(42.12 , 55.33)	17.33	(14.46 , 20.47)	7.66	(7.17 , 8.15)	3.21	(2.80 , 3.65)
2008093	4.12	(3.36 , 4.97)	6.04	(4.62 , 7.46)	40.20	(34.31 , 46.56)	5.21	(3.53 , 7.20)	6.59	(6.09 , 7.09)	2.01	(1.66 , 2.40)
2008108	5.75	(4.84 , 6.74)	11.37	(9.95 , 12.79)	57.26	(50.30 , 64.67)	13.63	(11.09 , 16.43)	7.77	(7.28 , 8.26)	3.30	(2.89 , 3.75)
2008179	4.11	(3.34 , 4.95)	6.67	(5.25 , 8.09)	42.08	(36.04 , 48.58)	5.68	(3.98 , 7.68)	7.18	(6.68 , 7.68)	2.02	(1.68 , 2.39)
2008192	4.37	(3.57 , 5.25)	6.20	(4.74 , 7.67)	40.00	(34.13 , 46.35)	11.66	(8.73 , 15.02)	6.59	(6.09 , 7.10)	3.18	(2.70 , 3.71)
2008089	3.69	(2.97 , 4.50)	6.91	(5.49 , 8.34)	37.48	(31.80 , 43.62)	9.00	(6.74 , 11.58)	6.43	(5.93 , 6.94)	2.57	(2.17 , 3.00)
2008064	4.40	(3.61 , 5.27)	8.29	(6.87 , 9.71)	45.41	(39.24 , 52.03)	12.03	(9.58 , 14.76)	7.04	(6.55 , 7.53)	2.87	(2.47 , 3.29)
2008269	4.82	(3.97 , 5.74)	7.86	(6.40 , 9.33)	46.21	(39.89 , 53.01)	6.68	(4.89 , 8.75)	7.48	(6.97 , 7.98)	2.59	(2.22 , 3.00)
2008059	4.12	(3.36 , 4.97)	5.25	(3.83 , 6.67)	39.97	(34.19 , 46.19)	6.14	(4.31 , 8.31)	6.73	(6.24 , 7.22)	2.21	(1.85 , 2.62)
2008047	4.98	(4.14 , 5.91)	5.98	(4.56 , 7.40)	42.84	(36.76 , 49.39)	3.88	(2.55 , 5.49)	6.98	(6.47 , 7.48)	1.86	(1.54 , 2.21)
2008194	4.07	(3.31 , 4.91)	7.54	(6.12 , 8.97)	44.31	(38.22 , 50.86)	7.94	(5.91 , 10.27)	7.09	(6.60 , 7.59)	2.49	(2.11 , 2.90)
2008232	3.87	(3.13 , 4.69)	7.20	(5.78 , 8.62)	39.21	(33.40 , 45.50)	8.82	(6.68 , 11.27)	6.41	(5.90 , 6.91)	2.41	(2.03 , 2.81)
2008173	4.14	(3.37 , 4.98)	8.57	(7.15 , 10.00)	38.79	(33.10 , 44.94)	7.67	(5.68 , 9.96)	6.36	(5.87 , 6.85)	2.59	(2.20 , 3.01)
2008120	3.78	(3.03 , 4.60)	6.54	(5.08 , 8.01)	43.80	(37.41 , 50.69)	7.62	(5.70 , 9.83)	6.98	(6.46 , 7.51)	2.50	(2.13 , 2.90)
2008262	4.53	(3.71 , 5.43)	6.88	(5.42 , 8.35)	39.70	(33.85 , 46.01)	7.02	(5.11 , 9.23)	6.70	(6.20 , 7.20)	2.36	(1.98 , 2.76)
2008187	4.56	(3.75 , 5.45)	8.55	(7.13 , 9.97)	45.77	(39.56 , 52.42)	10.89	(8.63 , 13.41)	6.79	(6.30 , 7.29)	2.95	(2.56 , 3.37)
2008051	3.87	(3.12 , 4.71)	7.16	(5.69 , 8.62)	42.20	(35.94 , 48.95)	12.41	(9.64 , 15.53)	6.82	(6.30 , 7.35)	3.16	(2.71 , 3.65)

Family	number of shoots 11 months *		number of shoots 24 months		length of the longest shoot 11 months *		length of the longest shoot 24 months *		number of nodes on the longest shoot 11 months		number of nodes on the longest shoot 24 months *	
2008198	3.94	(3.15 , 4.82)	7.05	(5.48 , 8.61)	45.98	(39.18 , 53.32)	9.41	(6.91 , 12.29)	7.32	(6.77 , 7.87)	2.76	(2.32 , 3.24)
2008054	4.84	(4.01 , 5.75)	7.12	(5.70 , 8.54)	47.29	(40.77 , 54.31)	7.25	(5.31 , 9.48)	7.58	(7.07 , 8.10)	2.57	(2.19 , 2.99)
2008244	4.96	(4.11 , 5.88)	5.50	(4.07 , 6.92)	43.53	(37.40 , 50.14)	7.96	(5.77 , 10.49)	7.11	(6.60 , 7.61)	2.36	(1.97 , 2.78)
2008177	3.40	(2.71 , 4.17)	5.09	(3.67 , 6.51)	42.07	(35.94 , 48.69)	5.78	(3.77 , 8.22)	6.71	(6.19 , 7.22)	2.27	(1.86 , 2.72)
2008117	4.58	(3.77 , 5.47)	6.71	(5.28 , 8.13)	48.15	(41.77 , 54.98)	10.53	(7.86 , 13.58)	6.87	(6.38 , 7.36)	2.98	(2.52 , 3.48)
2008016	5.09	(4.23 , 6.02)	6.54	(5.12 , 7.96)	45.67	(39.38 , 52.44)	13.02	(10.03 , 16.39)	7.00	(6.50 , 7.50)	3.12	(2.65 , 3.63)
2008267	4.20	(3.42 , 5.07)	7.86	(6.40 , 9.33)	47.33	(40.81 , 54.34)	8.79	(6.72 , 11.15)	7.19	(6.67 , 7.70)	2.65	(2.27 , 3.06)
2008040	4.00	(3.25 , 4.83)	7.69	(6.27 , 9.11)	47.76	(41.42 , 54.55)	10.43	(8.16 , 12.98)	6.98	(6.49 , 7.48)	2.84	(2.44 , 3.26)
2008218	4.12	(3.35 , 4.96)	6.94	(5.51 , 8.36)	45.24	(38.85 , 52.11)	7.43	(5.12 , 10.17)	7.26	(6.74 , 7.77)	2.44	(2.01 , 2.90)
2008070	4.49	(3.69 , 5.36)	5.70	(4.28 , 7.12)	42.74	(36.76 , 49.17)	10.24	(7.73 , 13.10)	6.81	(6.32 , 7.30)	2.54	(2.13 , 2.98)
2008102	3.02	(2.36 , 3.76)	5.83	(4.36 , 7.30)	39.53	(33.24 , 46.36)	5.37	(3.59 , 7.50)	6.70	(6.15 , 7.25)	2.11	(1.74 , 2.51)
2008078	6.22	(5.27 , 7.24)	10.85	(9.43 , 12.27)	45.74	(39.55 , 52.39)	10.67	(8.44 , 13.16)	7.32	(6.82 , 7.81)	2.79	(2.41 , 3.20)
2008074	4.06	(3.29 , 4.92)	8.54	(7.07 , 10.01)	39.36	(33.32 , 45.91)	10.93	(8.43 , 13.76)	6.42	(5.89 , 6.94)	2.89	(2.46 , 3.34)

b.

Family	height (at flower initiation) 13 months		height (at flower initiation) 25 months		height (mid-season) 14 months		height (mid-season) 26 months		height (at cone maturity) 16 months		height (at cone maturity) 28 months	
2008003	3.53	(3.30 , 3.77)	4.87	(4.66 , 5.09)	4.21	(3.97 , 4.44)	5.36	(5.23 , 5.49)	4.32	(4.14 , 4.50)	4.31	(4.13 , 4.50)
2008190	3.78	(3.55 , 4.02)	5.00	(4.79 , 5.21)	4.42	(4.18 , 4.66)	5.67	(5.54 , 5.80)	4.55	(4.36 , 4.73)	4.56	(4.37 , 4.74)
2008209	3.63	(3.40 , 3.87)	4.81	(4.59 , 5.02)	4.35	(4.09 , 4.61)	5.48	(5.34 , 5.61)	4.42	(4.24 , 4.61)	4.43	(4.24 , 4.62)
2008151	3.45	(3.21 , 3.68)	5.03	(4.81 , 5.24)	4.25	(4.01 , 4.49)	5.75	(5.62 , 5.88)	4.38	(4.20 , 4.57)	4.55	(4.36 , 4.74)
2008039	3.90	(3.66 , 4.13)	5.10	(4.89 , 5.32)	4.55	(4.31 , 4.78)	5.58	(5.45 , 5.71)	4.62	(4.44 , 4.81)	4.64	(4.45 , 4.82)
2008152	3.69	(3.45 , 3.92)	5.13	(4.92 , 5.35)	4.45	(4.20 , 4.70)	5.67	(5.54 , 5.80)	4.65	(4.46 , 4.83)	4.66	(4.48 , 4.85)
2008001	3.80	(3.56 , 4.05)	5.16	(4.94 , 5.39)	4.57	(4.32 , 4.81)	5.69	(5.55 , 5.83)	4.56	(4.36 , 4.75)	4.56	(4.37 , 4.76)
2008073	3.64	(3.40 , 3.87)	4.80	(4.59 , 5.02)	4.37	(4.13 , 4.61)	5.68	(5.55 , 5.81)	4.47	(4.29 , 4.65)	4.47	(4.29 , 4.66)
2008168	3.58	(3.35 , 3.82)	4.97	(4.75 , 5.18)	4.34	(4.10 , 4.58)	5.49	(5.36 , 5.62)	4.34	(4.16 , 4.53)	4.34	(4.15 , 4.53)
2008161	3.77	(3.54 , 4.01)	5.40	(5.19 , 5.61)	4.42	(4.18 , 4.65)	5.70	(5.57 , 5.84)	4.51	(4.32 , 4.69)	4.51	(4.33 , 4.70)
2008290	3.64	(3.39 , 3.88)	4.82	(4.59 , 5.05)	4.26	(4.02 , 4.50)	5.47	(5.33 , 5.61)	4.36	(4.17 , 4.56)	4.36	(4.17 , 4.56)
2008208	3.62	(3.38 , 3.86)	5.02	(4.80 , 5.23)	4.29	(4.05 , 4.53)	5.70	(5.57 , 5.83)	4.32	(4.14 , 4.50)	4.31	(4.13 , 4.50)
2008251	3.53	(3.30 , 3.77)	4.97	(4.76 , 5.19)	4.16	(3.92 , 4.40)	5.55	(5.42 , 5.68)	4.26	(4.08 , 4.44)	4.25	(4.07 , 4.43)
2008052	4.04	(3.80 , 4.28)	5.27	(5.05 , 5.48)	4.54	(4.30 , 4.78)	5.77	(5.64 , 5.91)	4.56	(4.37 , 4.75)	4.57	(4.38 , 4.76)

Family	height (at flower initiation) 13 months		height (at flower initiation) 25 months		height (mid-season) 14 months		height (mid-season) 26 months		height (at cone maturity) 16 months		height (at cone maturity) 28 months	
2008037	3.80	(3.56 , 4.04)	4.84	(4.62 , 5.05)	4.48	(4.24 , 4.73)	5.76	(5.62 , 5.89)	4.69	(4.50 , 4.88)	4.71	(4.52 , 4.90)
2008154	3.55	(3.31 , 3.78)	4.88	(4.66 , 5.09)	4.10	(3.86 , 4.34)	5.50	(5.37 , 5.63)	4.13	(3.95 , 4.31)	4.11	(3.93 , 4.30)
2008136	3.52	(3.29 , 3.76)	4.90	(4.69 , 5.11)	4.17	(3.93 , 4.41)	5.44	(5.31 , 5.57)	4.35	(4.16 , 4.53)	4.34	(4.15 , 4.52)
2008096	3.65	(3.41 , 3.88)	4.94	(4.73 , 5.15)	4.43	(4.19 , 4.67)	5.45	(5.32 , 5.58)	4.27	(4.09 , 4.45)	4.26	(4.08 , 4.45)
2008071	3.84	(3.61 , 4.08)	4.76	(4.54 , 4.97)	4.56	(4.32 , 4.81)	5.69	(5.56 , 5.83)	4.59	(4.41 , 4.77)	4.60	(4.42 , 4.78)
2008124	3.76	(3.53 , 4.00)	5.40	(5.19 , 5.61)	4.46	(4.22 , 4.70)	5.76	(5.63 , 5.89)	4.55	(4.37 , 4.74)	4.56	(4.38 , 4.75)
2008145	3.63	(3.39 , 3.86)	5.13	(4.92 , 5.34)	4.26	(4.02 , 4.51)	5.59	(5.46 , 5.72)	4.59	(4.41 , 4.77)	4.60	(4.42 , 4.79)
2008084	3.82	(3.59 , 4.06)	4.88	(4.67 , 5.09)	4.57	(4.34 , 4.81)	5.66	(5.52 , 5.79)	4.77	(4.58 , 4.95)	4.79	(4.60 , 4.97)
2008083	3.62	(3.39 , 3.86)	5.32	(5.11 , 5.54)	4.45	(4.21 , 4.68)	5.67	(5.54 , 5.80)	4.38	(4.20 , 4.57)	4.38	(4.20 , 4.56)
2008056	3.73	(3.49 , 3.96)	5.24	(5.03 , 5.46)	4.46	(4.23 , 4.70)	5.85	(5.72 , 5.98)	4.68	(4.50 , 4.87)	4.70	(4.52 , 4.89)
2008261	3.84	(3.61 , 4.08)	5.09	(4.88 , 5.31)	4.56	(4.32 , 4.79)	5.65	(5.52 , 5.78)	4.73	(4.55 , 4.92)	4.76	(4.57 , 4.94)
2008157	4.07	(3.84 , 4.31)	5.54	(5.32 , 5.75)	4.54	(4.30 , 4.78)	5.71	(5.58 , 5.84)	4.61	(4.42 , 4.79)	4.62	(4.44 , 4.80)
2008006	3.60	(3.37 , 3.84)	4.92	(4.71 , 5.14)	4.30	(4.06 , 4.55)	5.62	(5.49 , 5.75)	4.14	(3.96 , 4.33)	4.13	(3.94 , 4.31)
2008240	3.84	(3.60 , 4.07)	5.28	(5.07 , 5.50)	4.36	(4.13 , 4.60)	5.62	(5.49 , 5.75)	4.47	(4.29 , 4.65)	4.47	(4.29 , 4.66)
2008103	3.16	(2.92 , 3.39)	4.86	(4.65 , 5.07)	3.88	(3.65 , 4.12)	5.42	(5.29 , 5.55)	3.92	(3.73 , 4.10)	3.88	(3.70 , 4.06)
2008148	3.56	(3.33 , 3.80)	5.05	(4.83 , 5.26)	4.23	(3.98 , 4.48)	5.69	(5.56 , 5.83)	4.38	(4.20 , 4.57)	4.38	(4.19 , 4.56)
2008236	4.00	(3.77 , 4.24)	5.47	(5.26 , 5.68)	4.65	(4.41 , 4.89)	5.65	(5.52 , 5.79)	4.73	(4.54 , 4.91)	4.74	(4.56 , 4.93)
2008259	3.68	(3.45 , 3.92)	5.02	(4.81 , 5.23)	4.43	(4.20 , 4.67)	5.49	(5.36 , 5.62)	4.36	(4.18 , 4.54)	4.36	(4.17 , 4.54)
2008191	3.57	(3.33 , 3.81)	5.13	(4.91 , 5.35)	4.33	(4.09 , 4.57)	5.69	(5.56 , 5.83)	4.43	(4.24 , 4.62)	4.42	(4.24 , 4.61)
2008242	3.79	(3.55 , 4.03)	5.41	(5.19 , 5.62)	4.43	(4.18 , 4.69)	5.65	(5.51 , 5.78)	4.46	(4.28 , 4.65)	4.47	(4.28 , 4.65)
2008248	3.67	(3.43 , 3.91)	5.19	(4.97 , 5.41)	4.44	(4.20 , 4.69)	5.75	(5.62 , 5.89)	4.61	(4.42 , 4.80)	4.62	(4.43 , 4.81)
2008010	3.35	(3.12 , 3.59)	5.00	(4.79 , 5.21)	4.19	(3.96 , 4.43)	5.65	(5.52 , 5.78)	4.44	(4.26 , 4.62)	4.43	(4.25 , 4.62)
2008263	3.49	(3.25 , 3.72)	4.65	(4.43 , 4.86)	4.32	(4.09 , 4.56)	5.50	(5.37 , 5.63)	4.13	(3.94 , 4.31)	4.10	(3.92 , 4.29)
2008153	3.72	(3.48 , 3.95)	5.20	(4.99 , 5.41)	4.50	(4.26 , 4.74)	5.65	(5.52 , 5.79)	4.48	(4.30 , 4.67)	4.49	(4.30 , 4.67)
2008119	3.59	(3.35 , 3.82)	4.90	(4.69 , 5.12)	4.35	(4.11 , 4.60)	5.55	(5.41 , 5.68)	4.52	(4.34 , 4.70)	4.73	(4.54 , 4.92)
2008206	3.54	(3.30 , 3.77)	5.39	(5.18 , 5.60)	4.34	(4.10 , 4.57)	5.70	(5.57 , 5.83)	4.26	(4.08 , 4.44)	4.24	(4.06 , 4.43)
2008139	3.55	(3.31 , 3.78)	5.24	(5.03 , 5.45)	4.45	(4.21 , 4.70)	5.66	(5.53 , 5.79)	4.44	(4.26 , 4.62)	4.43	(4.25 , 4.62)
2008106	3.52	(3.28 , 3.75)	5.12	(4.90 , 5.33)	4.45	(4.21 , 4.68)	5.53	(5.40 , 5.66)	4.44	(4.25 , 4.62)	4.44	(4.25 , 4.62)
2008122	3.45	(3.21 , 3.69)	5.18	(4.97 , 5.40)	4.20	(3.96 , 4.44)	5.44	(5.31 , 5.58)	4.22	(4.03 , 4.41)	4.21	(4.02 , 4.40)
2008155	3.74	(3.50 , 3.97)	5.13	(4.92 , 5.34)	4.33	(4.10 , 4.57)	5.61	(5.48 , 5.75)	4.43	(4.25 , 4.62)	4.43	(4.25 , 4.62)
2008088	3.47	(3.24 , 3.71)	4.92	(4.71 , 5.13)	4.22	(3.98 , 4.45)	5.49	(5.36 , 5.63)	4.26	(4.08 , 4.45)	4.25	(4.07 , 4.44)
2008111	3.38	(3.15 , 3.62)	4.61	(4.40 , 4.83)	4.28	(4.04 , 4.53)	5.08	(4.95 , 5.21)	4.19	(4.00 , 4.37)	4.17	(3.99 , 4.36)
2008202	3.88	(3.64 , 4.12)	5.14	(4.92 , 5.36)	4.55	(4.31 , 4.79)	5.53	(5.40 , 5.67)	4.53	(4.35 , 4.72)	4.54	(4.35 , 4.73)
2008013	3.76	(3.53 , 4.00)	5.37	(5.16 , 5.58)	4.53	(4.29 , 4.76)	5.66	(5.53 , 5.79)	4.79	(4.61 , 4.97)	4.82	(4.64 , 5.00)
2008279	3.88	(3.64 , 4.11)	5.23	(5.02 , 5.45)	4.59	(4.36 , 4.83)	5.68	(5.55 , 5.81)	4.68	(4.50 , 4.86)	4.70	(4.52 , 4.89)
2008007	3.75	(3.51 , 3.99)	4.85	(4.64 , 5.06)	4.19	(3.96 , 4.43)	5.48	(5.35 , 5.62)	4.26	(4.07 , 4.44)	4.25	(4.06 , 4.44)

Family	height (at flower initiation) 13 months		height (at flower initiation) 25 months		height (mid-season) 14 months		height (mid-season) 26 months		height (at cone maturity) 16 months		height (at cone maturity) 28 months	
2008090	3.36	(3.13 , 3.60)	4.90	(4.69 , 5.11)	4.12	(3.88 , 4.36)	5.56	(5.43 , 5.69)	4.10	(3.92 , 4.28)	4.08	(3.89 , 4.26)
2008055	3.85	(3.62 , 4.09)	5.38	(5.17 , 5.60)	4.57	(4.34 , 4.81)	5.72	(5.59 , 5.86)	4.62	(4.44 , 4.81)	4.63	(4.45 , 4.82)
2008271	3.60	(3.36 , 3.83)	5.14	(4.93 , 5.35)	4.37	(4.13 , 4.61)	5.53	(5.40 , 5.66)	4.46	(4.27 , 4.64)	4.46	(4.27 , 4.64)
2008035	3.65	(3.41 , 3.88)	5.30	(5.08 , 5.51)	4.35	(4.11 , 4.58)	5.73	(5.60 , 5.86)	4.44	(4.25 , 4.62)	4.60	(4.41 , 4.79)
2008212	3.62	(3.39 , 3.86)	5.03	(4.82 , 5.25)	4.25	(4.01 , 4.48)	5.60	(5.47 , 5.73)	4.41	(4.22 , 4.59)	4.40	(4.22 , 4.59)
2008149	3.67	(3.44 , 3.91)	5.12	(4.90 , 5.33)	4.33	(4.09 , 4.57)	5.65	(5.52 , 5.78)	4.54	(4.35 , 4.72)	4.54	(4.36 , 4.73)
2008170	3.87	(3.63 , 4.10)	5.42	(5.21 , 5.63)	4.54	(4.30 , 4.77)	5.73	(5.60 , 5.86)	4.65	(4.47 , 4.84)	4.67	(4.48 , 4.85)
2008247	3.64	(3.40 , 3.87)	4.86	(4.65 , 5.08)	4.25	(4.01 , 4.49)	5.62	(5.49 , 5.75)	4.25	(4.07 , 4.43)	4.23	(4.05 , 4.41)
2008110	3.64	(3.40 , 3.88)	5.00	(4.78 , 5.22)	4.33	(4.09 , 4.57)	5.54	(5.41 , 5.68)	4.35	(4.15 , 4.54)	4.35	(4.15 , 4.54)
2008043	3.65	(3.42 , 3.89)	5.14	(4.93 , 5.36)	4.47	(4.24 , 4.71)	5.70	(5.57 , 5.83)	4.55	(4.36 , 4.73)	4.55	(4.37 , 4.74)
2008188	3.76	(3.53 , 4.00)	5.40	(5.19 , 5.61)	4.39	(4.16 , 4.63)	5.60	(5.47 , 5.74)	4.55	(4.36 , 4.73)	4.56	(4.37 , 4.74)
2008080	3.77	(3.52 , 4.01)	5.01	(4.78 , 5.23)	4.47	(4.22 , 4.71)	5.69	(5.56 , 5.83)	4.49	(4.29 , 4.69)	4.50	(4.30 , 4.70)
2008239	3.50	(3.27 , 3.74)	5.19	(4.97 , 5.40)	4.16	(3.92 , 4.40)	5.77	(5.64 , 5.90)	4.40	(4.22 , 4.59)	4.40	(4.22 , 4.59)
2008075	3.59	(3.36 , 3.83)	5.27	(5.06 , 5.48)	4.29	(4.05 , 4.52)	5.55	(5.42 , 5.68)	4.45	(4.26 , 4.63)	4.45	(4.26 , 4.63)
2008114	3.67	(3.42 , 3.91)	5.54	(5.31 , 5.76)	4.43	(4.19 , 4.67)	5.61	(5.47 , 5.75)	4.62	(4.43 , 4.82)	4.64	(4.44 , 4.83)
2008166	3.69	(3.45 , 3.92)	5.09	(4.88 , 5.30)	4.44	(4.20 , 4.68)	5.73	(5.60 , 5.86)	4.65	(4.47 , 4.83)	4.67	(4.48 , 4.85)
2008252	3.80	(3.56 , 4.03)	5.36	(5.15 , 5.57)	4.35	(4.11 , 4.59)	5.65	(5.52 , 5.78)	4.54	(4.35 , 4.73)	4.55	(4.36 , 4.74)
2008150	3.77	(3.53 , 4.00)	5.25	(5.04 , 5.46)	4.35	(4.11 , 4.59)	5.76	(5.63 , 5.89)	4.40	(4.22 , 4.58)	4.40	(4.22 , 4.58)
2008009	3.76	(3.53 , 4.00)	5.09	(4.88 , 5.31)	4.32	(4.09 , 4.56)	5.52	(5.39 , 5.66)	4.41	(4.23 , 4.60)	4.41	(4.23 , 4.60)
2008081	3.37	(3.13 , 3.62)	4.73	(4.51 , 4.96)	4.27	(4.01 , 4.53)	5.47	(5.34 , 5.61)	4.18	(3.98 , 4.37)	4.16	(3.96 , 4.35)
2008041	3.64	(3.40 , 3.88)	4.93	(4.72 , 5.14)	4.31	(4.07 , 4.55)	5.57	(5.44 , 5.70)	4.49	(4.31 , 4.67)	4.49	(4.31 , 4.68)
2008061	4.14	(3.90 , 4.38)	5.43	(5.21 , 5.65)	4.64	(4.40 , 4.88)	5.75	(5.62 , 5.89)	4.90	(4.71 , 5.09)	4.93	(4.74 , 5.12)
2008087	3.35	(3.12 , 3.59)	4.66	(4.45 , 4.88)	4.07	(3.84 , 4.31)	5.33	(5.20 , 5.46)	3.85	(3.66 , 4.03)	3.81	(3.63 , 4.00)
2008147	3.58	(3.35 , 3.82)	4.95	(4.74 , 5.17)	4.49	(4.25 , 4.72)	5.59	(5.46 , 5.73)	4.42	(4.24 , 4.60)	4.42	(4.24 , 4.60)
2008258	3.55	(3.31 , 3.78)	4.81	(4.60 , 5.02)	4.25	(4.01 , 4.49)	5.60	(5.47 , 5.73)	4.46	(4.27 , 4.64)	4.46	(4.27 , 4.64)
2008130	3.79	(3.55 , 4.03)	5.07	(4.86 , 5.28)	4.41	(4.18 , 4.65)	5.54	(5.41 , 5.67)	4.38	(4.20 , 4.56)	4.38	(4.19 , 4.56)
2008100	3.46	(3.22 , 3.70)	4.87	(4.65 , 5.09)	4.24	(3.99 , 4.49)	5.27	(5.14 , 5.41)	4.25	(4.06 , 4.44)	4.24	(4.05 , 4.43)
2008132	3.53	(3.30 , 3.77)	4.76	(4.55 , 4.97)	4.32	(4.09 , 4.56)	5.43	(5.30 , 5.56)	4.39	(4.21 , 4.57)	4.39	(4.21 , 4.58)
2008256	3.45	(3.21 , 3.68)	4.69	(4.47 , 4.90)	4.26	(4.02 , 4.49)	5.27	(5.14 , 5.40)	4.24	(4.06 , 4.43)	4.23	(4.05 , 4.42)
2008093	3.60	(3.36 , 3.83)	5.42	(5.20 , 5.63)	4.31	(4.07 , 4.55)	5.68	(5.54 , 5.81)	4.40	(4.22 , 4.58)	4.40	(4.22 , 4.58)
2008108	4.04	(3.81 , 4.28)	5.17	(4.96 , 5.38)	4.60	(4.36 , 4.83)	5.70	(5.57 , 5.83)	4.74	(4.56 , 4.93)	4.77	(4.59 , 4.95)
2008179	3.83	(3.59 , 4.06)	5.27	(5.06 , 5.49)	4.48	(4.25 , 4.72)	5.73	(5.59 , 5.86)	4.57	(4.39 , 4.76)	4.58	(4.40 , 4.77)
2008192	3.77	(3.53 , 4.01)	5.27	(5.05 , 5.49)	4.41	(4.17 , 4.65)	5.60	(5.46 , 5.73)	4.45	(4.27 , 4.64)	4.45	(4.26 , 4.64)
2008089	3.34	(3.10 , 3.57)	4.79	(4.57 , 5.00)	4.06	(3.83 , 4.30)	5.54	(5.41 , 5.67)	4.13	(3.95 , 4.31)	4.12	(3.93 , 4.30)
2008064	3.82	(3.58 , 4.05)	5.43	(5.21 , 5.64)	4.39	(4.15 , 4.62)	5.55	(5.42 , 5.68)	4.56	(4.37 , 4.74)	4.57	(4.38 , 4.75)
2008269	3.78	(3.54 , 4.02)	5.18	(4.96 , 5.39)	4.46	(4.22 , 4.70)	5.72	(5.58 , 5.85)	4.60	(4.41 , 4.79)	4.62	(4.43 , 4.81)

Family	height (at flower initiation) 13 months		height (at flower initiation) 25 months		height (mid-season) 14 months		height (mid-season) 26 months		height (at cone maturity) 16 months		height (at cone maturity) 28 months	
2008059	3.76	(3.52 , 3.99)	5.01	(4.79 , 5.22)	4.48	(4.25 , 4.72)	5.62	(5.49 , 5.76)	4.62	(4.44 , 4.80)	4.63	(4.45 , 4.82)
2008047	4.04	(3.80 , 4.27)	5.32	(5.10 , 5.53)	4.61	(4.36 , 4.85)	5.80	(5.67 , 5.94)	4.68	(4.50 , 4.87)	4.70	(4.52 , 4.89)
2008194	3.61	(3.38 , 3.85)	5.02	(4.81 , 5.23)	4.18	(3.93 , 4.42)	5.62	(5.49 , 5.75)	4.40	(4.22 , 4.58)	4.40	(4.22 , 4.59)
2008232	3.67	(3.44 , 3.91)	5.14	(4.93 , 5.36)	4.43	(4.19 , 4.68)	5.68	(5.54 , 5.81)	4.44	(4.26 , 4.62)	4.44	(4.26 , 4.62)
2008173	3.93	(3.69 , 4.16)	5.45	(5.24 , 5.66)	4.39	(4.15 , 4.64)	5.66	(5.53 , 5.79)	4.62	(4.44 , 4.80)	4.64	(4.45 , 4.82)
2008120	3.53	(3.29 , 3.77)	5.24	(5.02 , 5.46)	4.15	(3.91 , 4.39)	5.59	(5.46 , 5.73)	4.32	(4.13 , 4.51)	4.32	(4.13 , 4.51)
2008262	3.57	(3.33 , 3.81)	4.71	(4.50 , 4.93)	4.31	(4.07 , 4.55)	5.53	(5.40 , 5.67)	4.53	(4.34 , 4.72)	4.54	(4.35 , 4.73)
2008187	3.67	(3.44 , 3.91)	4.98	(4.77 , 5.20)	4.29	(4.04 , 4.53)	5.50	(5.37 , 5.63)	4.16	(3.98 , 4.34)	4.14	(3.95 , 4.32)
2008051	3.36	(3.13 , 3.60)	4.85	(4.64 , 5.07)	4.07	(3.83 , 4.31)	5.62	(5.49 , 5.76)	4.22	(4.03 , 4.41)	4.21	(4.02 , 4.40)
2008198	3.41	(3.16 , 3.66)	4.61	(4.38 , 4.85)	4.00	(3.76 , 4.25)	5.20	(5.06 , 5.34)	3.98	(3.78 , 4.18)	3.94	(3.74 , 4.14)
2008054	3.74	(3.51 , 3.98)	5.06	(4.84 , 5.28)	4.19	(3.95 , 4.43)	5.61	(5.48 , 5.74)	4.18	(3.99 , 4.36)	4.16	(3.98 , 4.34)
2008244	4.01	(3.77 , 4.24)	5.45	(5.24 , 5.66)	4.55	(4.31 , 4.79)	5.78	(5.65 , 5.91)	4.63	(4.44 , 4.81)	4.64	(4.46 , 4.82)
2008177	3.94	(3.70 , 4.18)	5.37	(5.16 , 5.58)	4.50	(4.24 , 4.75)	5.80	(5.67 , 5.94)	4.58	(4.40 , 4.76)	4.59	(4.41 , 4.77)
2008117	3.73	(3.49 , 3.97)	5.13	(4.92 , 5.34)	4.26	(4.02 , 4.51)	5.52	(5.38 , 5.65)	4.48	(4.30 , 4.67)	4.49	(4.30 , 4.67)
2008016	3.94	(3.71 , 4.18)	5.39	(5.18 , 5.60)	4.64	(4.40 , 4.89)	5.65	(5.52 , 5.78)	4.67	(4.49 , 4.86)	4.69	(4.51 , 4.87)
2008267	3.80	(3.56 , 4.04)	5.49	(5.27 , 5.70)	4.57	(4.32 , 4.81)	5.70	(5.56 , 5.83)	4.59	(4.40 , 4.78)	4.60	(4.41 , 4.79)
2008040	3.41	(3.18 , 3.65)	4.93	(4.72 , 5.15)	4.07	(3.83 , 4.31)	5.48	(5.35 , 5.61)	4.27	(4.08 , 4.46)	4.25	(4.07 , 4.44)
2008218	3.63	(3.40 , 3.87)	5.16	(4.95 , 5.38)	4.37	(4.14 , 4.61)	5.73	(5.60 , 5.86)	4.37	(4.19 , 4.55)	4.36	(4.18 , 4.55)
2008070	3.70	(3.46 , 3.93)	5.04	(4.83 , 5.26)	4.39	(4.15 , 4.64)	5.72	(5.59 , 5.85)	4.54	(4.35 , 4.72)	4.55	(4.36 , 4.73)
2008102	3.36	(3.12 , 3.60)	4.75	(4.53 , 4.98)	4.13	(3.88 , 4.38)	5.51	(5.37 , 5.65)	4.05	(3.86 , 4.24)	4.03	(3.84 , 4.21)
2008078	3.52	(3.28 , 3.75)	4.69	(4.47 , 4.90)	4.29	(4.05 , 4.52)	5.56	(5.43 , 5.69)	4.32	(4.14 , 4.51)	4.32	(4.14 , 4.50)
2008074	3.52	(3.28 , 3.76)	5.25	(5.03 , 5.47)	4.20	(3.95 , 4.45)	5.72	(5.58 , 5.85)	4.45	(4.27 , 4.64)	4.46	(4.27 , 4.64)

c.

Family	lateral length 16 months *		lateral length 28 months *		number of nodes on lateral 16 months *		number of nodes on lateral 28 months *		internode length 16 months *		internode length 28 months *	
2008003	43.18	(38.64 , 47.98)	38.57	(33.70 , 43.77)	6.43	(5.91 , 6.98)	6.42	(5.90 , 6.96)	15.41	(14.08 , 16.79)	22.56	(20.40 , 24.83)
2008190	42.65	(38.14 , 47.42)	35.04	(30.41 , 40.00)	5.55	(5.07 , 6.06)	5.57	(5.09 , 6.07)	17.60	(16.19 , 19.07)	22.56	(20.40 , 24.83)
2008209	41.69	(36.97 , 46.68)	35.65	(30.85 , 40.79)	5.94	(5.41 , 6.49)	5.94	(5.42 , 6.49)	15.67	(14.24 , 17.17)	22.56	(20.40 , 24.83)
2008151	47.61	(42.83 , 52.64)	39.59	(34.59 , 44.91)	5.89	(5.39 , 6.42)	5.90	(5.41 , 6.42)	18.41	(16.74 , 20.16)	22.56	(20.40 , 24.83)
2008039	45.53	(40.86 , 50.45)	47.60	(42.17 , 53.36)	6.39	(5.86 , 6.94)	6.39	(5.86 , 6.93)	17.30	(15.90 , 18.77)	22.56	(20.40 , 24.83)

Family	lateral length 16 months *		lateral length 28 months *		number of nodes on lateral 16 months *		number of nodes on lateral 28 months *		internode length 16 months *		internode length 28 months *	
2008152	47.20	(42.43 , 52.21)	42.21	(37.11 , 47.64)	5.98	(5.48 , 6.51)	5.99	(5.49 , 6.51)	17.88	(16.45 , 19.37)	22.56	(20.40 , 24.83)
2008001	39.52	(35.02 , 44.29)	33.27	(28.65 , 38.23)	5.41	(4.91 , 5.93)	5.43	(4.94 , 5.94)	18.48	(16.94 , 20.10)	22.56	(20.40 , 24.83)
2008073	46.44	(41.73 , 51.41)	40.86	(35.85 , 46.20)	6.73	(6.20 , 7.29)	6.72	(6.19 , 7.27)	19.24	(17.53 , 21.03)	22.56	(20.40 , 24.83)
2008168	42.44	(37.94 , 47.20)	37.71	(32.76 , 43.01)	6.16	(5.65 , 6.70)	6.17	(5.66 , 6.69)	17.96	(16.69 , 19.29)	22.56	(20.40 , 24.83)
2008161	44.27	(39.67 , 49.13)	38.26	(33.42 , 43.42)	6.06	(5.55 , 6.59)	6.07	(5.56 , 6.59)	17.95	(16.51 , 19.45)	22.56	(20.40 , 24.83)
2008290	41.10	(36.51 , 45.96)	34.88	(30.14 , 39.98)	5.83	(5.31 , 6.37)	5.84	(5.33 , 6.37)	16.69	(15.10 , 18.36)	22.56	(20.40 , 24.83)
2008208	44.52	(39.91 , 49.40)	37.67	(32.87 , 42.80)	5.82	(5.32 , 6.34)	5.83	(5.34 , 6.34)	16.47	(15.10 , 17.90)	22.56	(20.40 , 24.83)
2008251	39.91	(35.47 , 44.62)	32.89	(28.35 , 37.75)	5.76	(5.26 , 6.29)	5.78	(5.28 , 6.30)	17.61	(15.83 , 19.49)	22.56	(20.40 , 24.83)
2008052	45.01	(40.29 , 50.00)	37.74	(32.78 , 43.05)	6.06	(5.55 , 6.60)	6.07	(5.56 , 6.60)	20.78	(19.24 , 22.37)	22.56	(20.40 , 24.83)
2008037	45.77	(41.00 , 50.79)	39.61	(34.60 , 44.96)	6.13	(5.61 , 6.67)	6.13	(5.62 , 6.67)	18.45	(16.78 , 20.21)	22.56	(20.40 , 24.83)
2008154	44.73	(40.10 , 49.61)	39.11	(34.21 , 44.34)	5.96	(5.46 , 6.49)	5.97	(5.47 , 6.49)	15.81	(14.27 , 17.44)	22.56	(20.40 , 24.83)
2008136	45.17	(40.43 , 50.17)	36.52	(31.66 , 41.73)	5.83	(5.32 , 6.36)	5.84	(5.34 , 6.36)	13.89	(12.70 , 15.12)	22.56	(20.40 , 24.83)
2008096	42.06	(37.58 , 46.80)	37.32	(32.55 , 42.41)	5.88	(5.38 , 6.40)	5.89	(5.39 , 6.40)	14.93	(13.63 , 16.29)	22.56	(20.40 , 24.83)
2008071	45.61	(40.92 , 50.55)	37.05	(32.21 , 42.23)	5.77	(5.28 , 6.29)	5.78	(5.29 , 6.30)	19.49	(17.76 , 21.29)	22.56	(20.40 , 24.83)
2008124	50.90	(45.96 , 56.09)	43.91	(38.70 , 49.44)	6.32	(5.80 , 6.86)	6.32	(5.80 , 6.85)	19.71	(17.98 , 21.53)	22.56	(20.40 , 24.83)
2008145	44.37	(39.75 , 49.24)	38.45	(33.59 , 43.65)	5.97	(5.46 , 6.49)	5.97	(5.47 , 6.50)	18.63	(17.16 , 20.16)	22.56	(20.40 , 24.83)
2008084	43.41	(38.85 , 48.21)	37.45	(32.49 , 42.77)	6.15	(5.63 , 6.68)	6.15	(5.64 , 6.68)	16.01	(14.65 , 17.43)	22.56	(20.40 , 24.83)
2008083	44.62	(40.00 , 49.49)	38.13	(33.31 , 43.28)	6.33	(5.81 , 6.87)	6.33	(5.81 , 6.86)	16.20	(14.84 , 17.61)	22.56	(20.40 , 24.83)
2008056	48.87	(44.04 , 53.96)	40.93	(35.93 , 46.26)	7.11	(6.56 , 7.68)	7.09	(6.54 , 7.65)	19.30	(17.82 , 20.84)	22.56	(20.40 , 24.83)
2008261	47.59	(42.81 , 52.61)	40.62	(35.63 , 45.93)	6.04	(5.53 , 6.57)	6.04	(5.54 , 6.56)	17.60	(16.34 , 18.91)	22.56	(20.40 , 24.83)
2008157	49.31	(44.45 , 54.43)	43.31	(38.17 , 48.77)	6.12	(5.61 , 6.65)	6.12	(5.62 , 6.65)	16.96	(15.35 , 18.64)	22.56	(20.40 , 24.83)
2008006	37.71	(33.31 , 42.37)	31.99	(27.46 , 36.87)	5.53	(5.03 , 6.05)	5.55	(5.05 , 6.06)	17.21	(15.59 , 18.90)	22.56	(20.40 , 24.83)
2008240	47.24	(42.48 , 52.25)	41.20	(36.17 , 46.56)	6.92	(6.37 , 7.48)	6.90	(6.36 , 7.46)	14.92	(13.42 , 16.50)	22.56	(20.40 , 24.83)
2008103	36.81	(32.46 , 41.42)	32.13	(27.56 , 37.04)	5.61	(5.11 , 6.14)	5.63	(5.13 , 6.15)	15.37	(14.05 , 16.75)	22.56	(20.40 , 24.83)
2008148	41.28	(36.83 , 45.98)	36.04	(31.34 , 41.06)	5.80	(5.30 , 6.32)	5.81	(5.32 , 6.33)	15.37	(13.84 , 16.98)	22.56	(20.40 , 24.83)
2008236	49.00	(44.15 , 54.10)	40.65	(35.64 , 45.99)	6.59	(6.06 , 7.14)	6.58	(6.06 , 7.13)	17.49	(16.07 , 18.96)	22.56	(20.40 , 24.83)
2008259	43.98	(39.32 , 48.90)	39.81	(34.80 , 45.16)	5.89	(5.38 , 6.42)	5.90	(5.40 , 6.42)	16.73	(15.50 , 18.01)	22.56	(20.40 , 24.83)
2008191	45.48	(40.72 , 50.50)	41.44	(36.32 , 46.91)	5.97	(5.46 , 6.51)	5.98	(5.47 , 6.51)	16.63	(15.39 , 17.92)	22.56	(20.40 , 24.83)
2008242	43.02	(38.39 , 47.92)	35.83	(31.08 , 40.92)	6.20	(5.68 , 6.75)	6.20	(5.69 , 6.74)	16.16	(14.59 , 17.81)	22.56	(20.40 , 24.83)
2008248	44.44	(39.74 , 49.39)	38.16	(33.25 , 43.42)	6.18	(5.66 , 6.73)	6.18	(5.67 , 6.72)	16.83	(15.45 , 18.27)	22.56	(20.40 , 24.83)
2008010	45.81	(41.13 , 50.75)	39.65	(34.72 , 44.90)	6.39	(5.87 , 6.94)	6.39	(5.87 , 6.93)	16.48	(14.90 , 18.15)	22.56	(20.40 , 24.83)
2008263	45.54	(40.87 , 50.46)	40.06	(35.10 , 45.35)	6.41	(5.89 , 6.96)	6.41	(5.89 , 6.95)	18.09	(16.66 , 19.59)	22.56	(20.40 , 24.83)
2008153	43.95	(39.36 , 48.80)	37.29	(32.51 , 42.41)	6.12	(5.61 , 6.65)	6.12	(5.61 , 6.65)	16.41	(15.04 , 17.85)	22.56	(20.40 , 24.83)
2008119	44.96	(40.32 , 49.86)	38.80	(33.85 , 44.09)	6.01	(5.50 , 6.54)	6.01	(5.51 , 6.54)	16.10	(14.74 , 17.52)	22.56	(20.40 , 24.83)
2008206	41.40	(36.95 , 46.11)	37.55	(32.75 , 42.68)	6.06	(5.55 , 6.59)	6.06	(5.56 , 6.59)	17.09	(15.48 , 18.79)	22.56	(20.40 , 24.83)
2008139	43.74	(39.07 , 48.66)	37.24	(32.40 , 42.42)	6.02	(5.51 , 6.56)	6.03	(5.52 , 6.56)	17.90	(16.25 , 19.63)	22.56	(20.40 , 24.83)

Family	lateral length 16 months *		lateral length 28 months *		number of nodes on lateral 16 months *		number of nodes on lateral 28 months *		internode length 16 months *		internode length 28 months *	
2008106	43.96	(39.37 , 48.80)	37.68	(32.89 , 42.81)	5.98	(5.48 , 6.51)	5.99	(5.49 , 6.51)	18.06	(16.63 , 19.55)	22.56	(20.40 , 24.83)
2008122	40.53	(35.97 , 45.36)	34.81	(30.07 , 39.90)	6.13	(5.60 , 6.68)	6.13	(5.61 , 6.68)	15.52	(13.99 , 17.13)	22.56	(20.40 , 24.83)
2008155	44.54	(39.92 , 49.41)	39.05	(34.16 , 44.26)	6.19	(5.67 , 6.72)	6.19	(5.68 , 6.72)	16.49	(15.12 , 17.92)	22.56	(20.40 , 24.83)
2008088	41.00	(36.49 , 45.77)	35.14	(30.38 , 40.25)	5.74	(5.23 , 6.26)	5.75	(5.25 , 6.27)	15.41	(13.74 , 17.18)	22.56	(20.40 , 24.83)
2008111	41.25	(36.82 , 45.94)	36.27	(31.42 , 41.47)	5.83	(5.34 , 6.35)	5.84	(5.35 , 6.36)	15.98	(14.64 , 17.38)	22.56	(20.40 , 24.83)
2008202	46.69	(41.88 , 51.77)	40.23	(35.19 , 45.60)	6.50	(5.96 , 7.05)	6.49	(5.96 , 7.04)	16.67	(15.09 , 18.34)	22.56	(20.40 , 24.83)
2008013	49.25	(44.39 , 54.36)	43.68	(38.51 , 49.18)	6.34	(5.82 , 6.88)	6.33	(5.82 , 6.87)	18.37	(17.08 , 19.71)	22.56	(20.40 , 24.83)
2008279	44.75	(40.13 , 49.63)	39.27	(34.37 , 44.50)	5.98	(5.47 , 6.50)	5.98	(5.48 , 6.50)	19.10	(17.40 , 20.88)	22.56	(20.40 , 24.83)
2008007	52.02	(46.83 , 57.48)	44.75	(39.34 , 50.50)	6.48	(5.94 , 7.04)	6.47	(5.93 , 7.03)	17.00	(15.40 , 18.68)	22.56	(20.40 , 24.83)
2008090	42.79	(38.18 , 47.67)	36.77	(31.96 , 41.92)	6.27	(5.75 , 6.82)	6.27	(5.75 , 6.82)	16.66	(15.08 , 18.33)	22.56	(20.40 , 24.83)
2008055	47.09	(42.33 , 52.10)	38.91	(33.96 , 44.19)	6.57	(6.04 , 7.13)	6.56	(6.04 , 7.11)	16.66	(15.07 , 18.32)	22.56	(20.40 , 24.83)
2008271	45.48	(40.81 , 50.41)	38.13	(33.23 , 43.36)	6.27	(5.75 , 6.81)	6.27	(5.76 , 6.80)	17.90	(16.25 , 19.64)	22.56	(20.40 , 24.83)
2008035	44.68	(39.97 , 49.64)	40.60	(35.54 , 46.00)	5.89	(5.38 , 6.42)	5.89	(5.39 , 6.42)	16.90	(15.51 , 18.35)	22.56	(20.40 , 24.83)
2008212	45.09	(40.45 , 49.99)	38.92	(34.04 , 44.13)	6.35	(5.83 , 6.89)	6.35	(5.83 , 6.88)	17.56	(15.93 , 19.28)	22.56	(20.40 , 24.83)
2008149	42.83	(38.31 , 47.61)	36.11	(31.41 , 41.13)	6.06	(5.55 , 6.59)	6.06	(5.56 , 6.58)	17.12	(15.15 , 19.21)	22.56	(20.40 , 24.83)
2008170	48.87	(44.03 , 53.97)	41.92	(36.85 , 47.32)	6.27	(5.75 , 6.81)	6.27	(5.75 , 6.80)	17.12	(15.15 , 19.21)	22.56	(20.40 , 24.83)
2008247	39.06	(34.74 , 43.63)	32.53	(28.02 , 37.38)	5.63	(5.14 , 6.14)	5.64	(5.16 , 6.15)	17.12	(15.15 , 19.21)	22.56	(20.40 , 24.83)
2008110	37.49	(33.11 , 42.15)	32.22	(27.66 , 37.12)	5.57	(5.06 , 6.10)	5.67	(5.16 , 6.19)	15.16	(13.64 , 16.75)	22.56	(20.40 , 24.83)
2008043	45.44	(40.68 , 50.46)	39.85	(34.84 , 45.20)	6.17	(5.65 , 6.72)	6.17	(5.66 , 6.71)	17.12	(15.15 , 19.21)	22.56	(20.40 , 24.83)
2008188	43.19	(38.64 , 47.98)	36.28	(31.52 , 41.38)	6.55	(6.02 , 7.10)	6.54	(6.02 , 7.09)	17.37	(15.75 , 19.08)	22.56	(20.40 , 24.83)
2008080	45.10	(40.29 , 50.18)	38.14	(33.19 , 43.44)	6.12	(5.59 , 6.67)	6.12	(5.60 , 6.66)	17.42	(15.65 , 19.29)	22.56	(20.40 , 24.83)
2008239	43.64	(38.90 , 48.65)	38.58	(33.59 , 43.91)	6.21	(5.67 , 6.76)	6.21	(5.68 , 6.75)	17.12	(15.15 , 19.21)	22.56	(20.40 , 24.83)
2008075	45.03	(40.38 , 49.93)	39.53	(34.60 , 44.78)	6.20	(5.69 , 6.74)	6.20	(5.69 , 6.74)	17.12	(15.15 , 19.21)	22.56	(20.40 , 24.83)
2008114	41.47	(36.85 , 46.36)	35.90	(31.08 , 41.06)	5.95	(5.43 , 6.49)	5.95	(5.44 , 6.49)	17.12	(15.15 , 19.21)	22.56	(20.40 , 24.83)
2008166	45.54	(40.87 , 50.46)	38.58	(33.71 , 43.78)	6.37	(5.85 , 6.92)	6.37	(5.86 , 6.91)	17.12	(15.15 , 19.21)	22.56	(20.40 , 24.83)
2008252	45.87	(41.18 , 50.82)	39.46	(34.54 , 44.70)	6.19	(5.67 , 6.72)	6.19	(5.68 , 6.72)	17.12	(15.15 , 19.21)	22.56	(20.40 , 24.83)
2008150	45.34	(40.68 , 50.26)	39.25	(34.35 , 44.48)	6.18	(5.66 , 6.71)	6.18	(5.67 , 6.71)	17.12	(15.15 , 19.21)	22.56	(20.40 , 24.83)
2008009	41.42	(36.96 , 46.12)	35.53	(30.88 , 40.51)	5.95	(5.45 , 6.48)	5.96	(5.46 , 6.48)	17.12	(15.15 , 19.21)	22.56	(20.40 , 24.83)
2008081	44.80	(40.00 , 49.87)	39.04	(33.93 , 44.51)	6.15	(5.62 , 6.70)	6.15	(5.63 , 6.69)	17.09	(15.48 , 18.79)	22.56	(20.40 , 24.83)
2008041	42.81	(38.29 , 47.60)	36.95	(32.17 , 42.05)	6.00	(5.49 , 6.53)	6.01	(5.51 , 6.53)	17.32	(15.70 , 19.02)	22.56	(20.40 , 24.83)
2008061	50.19	(45.20 , 55.45)	43.53	(38.29 , 49.11)	6.68	(6.14 , 7.24)	6.66	(6.13 , 7.22)	17.69	(16.26 , 19.18)	22.56	(20.40 , 24.83)
2008087	36.18	(31.80 , 40.84)	31.63	(26.96 , 36.68)	5.29	(4.80 , 5.81)	5.31	(4.82 , 5.83)	15.44	(13.91 , 17.05)	22.56	(20.40 , 24.83)
2008147	48.46	(43.54 , 53.64)	40.04	(34.94 , 45.50)	6.49	(5.95 , 7.04)	6.48	(5.95 , 7.03)	17.12	(15.15 , 19.21)	22.56	(20.40 , 24.83)
2008258	43.12	(38.58 , 47.92)	33.62	(29.10 , 38.47)	6.10	(5.59 , 6.64)	6.11	(5.60 , 6.63)	17.12	(15.15 , 19.21)	22.56	(20.40 , 24.83)
2008130	40.86	(36.44 , 45.53)	33.78	(29.23 , 38.65)	5.80	(5.30 , 6.32)	5.81	(5.32 , 6.33)	17.12	(15.15 , 19.21)	22.56	(20.40 , 24.83)
2008100	38.27	(33.92 , 42.87)	33.85	(29.25 , 38.79)	5.48	(4.99 , 6.00)	5.50	(5.01 , 6.01)	17.64	(16.01 , 19.35)	22.56	(20.40 , 24.83)

Family	**lateral length** 16 months *		**lateral length** 28 months *		**number of nodes on lateral** 16 months *		**number of nodes on lateral** 28 months *		**internode length** 16 months *		**internode length** 28 months *	
2008132	44.66	(40.04 , 49.53)	36.65	(31.78 , 41.86)	6.35	(5.83 , 6.89)	6.35	(5.83 , 6.88)	17.12	(15.15 , 19.21)	22.56	(20.40 , 24.83)
2008256	37.40	(33.19 , 41.87)	33.95	(29.34 , 38.89)	5.39	(4.91 , 5.89)	5.40	(4.93 , 5.90)	17.86	(16.22 , 19.59)	22.56	(20.40 , 24.83)
2008093	39.23	(34.91 , 43.81)	33.21	(28.72 , 38.03)	5.93	(5.43 , 6.46)	5.94	(5.44 , 6.46)	17.12	(15.15 , 19.21)	22.56	(20.40 , 24.83)
2008108	51.32	(46.36 , 56.54)	40.98	(35.96 , 46.33)	6.41	(5.89 , 6.95)	6.40	(5.89 , 6.94)	15.88	(14.33 , 17.51)	22.56	(20.40 , 24.83)
2008179	46.36	(41.64 , 51.33)	40.81	(35.81 , 46.14)	6.87	(6.33 , 7.43)	6.85	(6.32 , 7.41)	18.54	(16.86 , 20.29)	22.56	(20.40 , 24.83)
2008192	43.89	(39.23 , 48.82)	38.56	(33.65 , 43.81)	5.84	(5.33 , 6.37)	5.85	(5.35 , 6.37)	17.29	(15.52 , 19.16)	22.56	(20.40 , 24.83)
2008089	38.04	(33.71 , 42.64)	32.29	(27.80 , 37.12)	5.78	(5.27 , 6.30)	5.79	(5.29 , 6.31)	17.11	(15.71 , 18.57)	22.56	(20.40 , 24.83)
2008064	44.82	(40.11 , 49.79)	38.75	(33.82 , 44.02)	6.45	(5.92 , 7.00)	6.44	(5.92 , 6.99)	17.12	(15.15 , 19.21)	22.56	(20.40 , 24.83)
2008269	44.80	(40.09 , 49.77)	39.54	(34.38 , 45.05)	6.23	(5.70 , 6.79)	6.23	(5.71 , 6.78)	17.12	(15.15 , 19.21)	22.56	(20.40 , 24.83)
2008059	49.83	(44.94 , 54.97)	43.34	(38.17 , 48.84)	6.05	(5.54 , 6.58)	6.05	(5.55 , 6.57)	16.63	(15.04 , 18.30)	22.56	(20.40 , 24.83)
2008047	44.58	(39.88 , 49.54)	36.46	(31.52 , 41.76)	5.78	(5.27 , 6.30)	5.79	(5.29 , 6.31)	17.12	(15.15 , 19.21)	22.56	(20.40 , 24.83)
2008194	41.09	(36.66 , 45.77)	34.55	(29.89 , 39.56)	5.90	(5.39 , 6.42)	5.90	(5.41 , 6.42)	17.12	(15.15 , 19.21)	22.56	(20.40 , 24.83)
2008232	39.94	(35.58 , 44.56)	34.31	(29.60 , 39.37)	6.50	(5.97 , 7.05)	6.50	(5.97 , 7.04)	17.09	(15.47 , 18.78)	22.56	(20.40 , 24.83)
2008173	45.47	(40.80 , 50.39)	39.90	(34.95 , 45.17)	6.21	(5.69 , 6.76)	6.21	(5.69 , 6.75)	17.12	(15.15 , 19.21)	22.56	(20.40 , 24.83)
2008120	38.12	(33.70 , 42.82)	31.61	(27.10 , 36.46)	5.46	(4.96 , 5.98)	5.48	(4.98 , 5.99)	16.60	(15.13 , 18.14)	22.56	(20.40 , 24.83)
2008262	49.57	(44.61 , 54.79)	45.03	(39.69 , 50.72)	6.47	(5.94 , 7.03)	6.46	(5.94 , 7.01)	18.49	(17.04 , 20.00)	22.56	(20.40 , 24.83)
2008187	39.11	(34.71 , 43.77)	35.79	(31.05 , 40.87)	5.72	(5.22 , 6.24)	5.73	(5.23 , 6.25)	17.12	(15.15 , 19.21)	22.56	(20.40 , 24.83)
2008051	41.25	(36.73 , 46.02)	36.02	(31.26 , 41.10)	6.02	(5.49 , 6.56)	6.02	(5.50 , 6.56)	17.12	(15.15 , 19.21)	22.56	(20.40 , 24.83)
2008198	38.81	(34.17 , 43.75)	32.91	(28.16 , 38.03)	5.57	(5.05 , 6.12)	5.59	(5.07 , 6.12)	16.79	(15.05 , 18.63)	22.56	(20.40 , 24.83)
2008054	39.64	(35.29 , 44.25)	35.98	(31.29 , 40.99)	5.70	(5.21 , 6.22)	5.71	(5.23 , 6.22)	17.12	(15.15 , 19.21)	22.56	(20.40 , 24.83)
2008244	43.15	(38.61 , 47.94)	37.33	(32.55 , 42.45)	6.56	(6.03 , 7.11)	6.56	(6.03 , 7.10)	17.12	(15.15 , 19.21)	22.56	(20.40 , 24.83)
2008177	48.94	(44.01 , 54.13)	41.28	(36.12 , 46.80)	6.37	(5.84 , 6.92)	6.37	(5.85 , 6.91)	17.12	(15.15 , 19.21)	22.56	(20.40 , 24.83)
2008117	42.08	(37.59 , 46.83)	33.10	(28.59 , 37.94)	5.88	(5.38 , 6.40)	5.88	(5.39 , 6.40)	17.98	(16.32 , 19.71)	22.56	(20.40 , 24.83)
2008016	47.87	(42.99 , 53.01)	44.12	(38.84 , 49.74)	6.42	(5.89 , 6.98)	6.42	(5.89 , 6.97)	17.12	(15.15 , 19.21)	22.56	(20.40 , 24.83)
2008267	45.94	(41.17 , 50.98)	41.40	(36.29 , 46.84)	6.23	(5.71 , 6.78)	6.23	(5.71 , 6.77)	19.65	(17.92 , 21.45)	22.56	(20.40 , 24.83)
2008040	44.73	(39.93 , 49.81)	38.96	(33.94 , 44.34)	5.83	(5.31 , 6.36)	5.84	(5.33 , 6.37)	17.14	(15.53 , 18.83)	22.56	(20.40 , 24.83)
2008218	44.14	(39.54 , 48.99)	33.74	(29.20 , 38.61)	6.23	(5.71 , 6.77)	6.23	(5.72 , 6.76)	17.51	(15.88 , 19.22)	22.56	(20.40 , 24.83)
2008070	43.65	(39.09 , 48.47)	38.39	(33.49 , 43.64)	6.37	(5.85 , 6.91)	6.36	(5.85 , 6.90)	17.12	(15.15 , 19.21)	22.56	(20.40 , 24.83)
2008102	38.90	(34.35 , 43.74)	33.03	(28.35 , 38.08)	6.16	(5.62 , 6.72)	6.16	(5.63 , 6.72)	17.12	(15.15 , 19.21)	22.56	(20.40 , 24.83)
2008078	42.14	(37.66 , 46.87)	36.55	(31.83 , 41.59)	6.03	(5.52 , 6.55)	6.03	(5.53 , 6.55)	16.00	(14.80 , 17.24)	22.56	(20.40 , 24.83)
2008074	43.86	(39.19 , 48.79)	35.62	(30.88 , 40.70)	6.06	(5.54 , 6.61)	6.07	(5.55 , 6.61)	17.12	(15.15 , 19.21)	22.56	(20.40 , 24.83)

d.

Family	height to the cones 16 months		height to the cones 28 months		green cone weight 28 months *	
2008003	1.68	(1.54 , 1.83)	1.68	(1.52 , 1.84)	0.94	(0.82 , 1.08)
2008190	1.80	(1.66 , 1.94)	1.82	(1.67 , 1.97)	0.91	(0.78 , 1.04)
2008209	1.85	(1.70 , 1.99)	1.87	(1.71 , 2.04)	0.90	(0.78 , 1.03)
2008151	1.76	(1.61 , 1.91)	1.71	(1.54 , 1.88)	0.89	(0.77 , 1.03)
2008039	1.88	(1.73 , 2.02)	1.91	(1.75 , 2.06)	0.93	(0.80 , 1.07)
2008152	1.86	(1.72 , 1.99)	1.89	(1.75 , 2.04)	0.86	(0.74 , 0.99)
2008001	1.86	(1.71 , 2.00)	1.89	(1.72 , 2.05)	0.93	(0.81 , 1.07)
2008073	1.90	(1.75 , 2.05)	1.95	(1.79 , 2.12)	0.92	(0.79 , 1.06)
2008168	1.62	(1.47 , 1.76)	1.59	(1.43 , 1.74)	0.93	(0.80 , 1.06)
2008161	1.78	(1.64 , 1.92)	1.77	(1.61 , 1.94)	0.93	(0.81 , 1.06)
2008290	1.72	(1.58 , 1.87)	1.73	(1.57 , 1.89)	0.85	(0.73 , 0.98)
2008208	1.61	(1.47 , 1.75)	1.59	(1.44 , 1.74)	0.95	(0.82 , 1.09)
2008251	1.84	(1.69 , 1.98)	1.86	(1.71 , 2.02)	0.88	(0.76 , 1.01)
2008052	1.83	(1.68 , 1.98)	1.86	(1.69 , 2.02)	0.87	(0.74 , 1.00)
2008037	1.77	(1.61 , 1.92)	1.79	(1.62 , 1.96)	0.92	(0.80 , 1.06)
2008154	1.61	(1.47 , 1.75)	1.63	(1.47 , 1.79)	0.94	(0.82 , 1.08)
2008136	1.63	(1.49 , 1.77)	1.63	(1.47 , 1.79)	0.84	(0.72 , 0.97)
2008096	1.76	(1.62 , 1.91)	1.78	(1.62 , 1.93)	0.94	(0.81 , 1.08)
2008071	1.85	(1.72 , 1.99)	1.88	(1.74 , 2.03)	0.93	(0.81 , 1.07)
2008124	1.83	(1.69 , 1.98)	1.86	(1.70 , 2.03)	0.91	(0.78 , 1.04)
2008145	1.60	(1.47 , 1.74)	1.57	(1.43 , 1.72)	0.93	(0.81 , 1.07)
2008084	1.85	(1.71 , 2.00)	1.91	(1.74 , 2.08)	0.91	(0.78 , 1.05)
2008083	1.79	(1.64 , 1.93)	1.96	(1.79 , 2.13)	0.88	(0.75 , 1.01)
2008056	1.84	(1.68 , 2.00)	1.89	(1.71 , 2.06)	0.92	(0.79 , 1.06)
2008261	1.80	(1.65 , 1.95)	1.83	(1.67 , 1.99)	0.91	(0.78 , 1.04)
2008157	1.75	(1.60 , 1.89)	1.76	(1.60 , 1.92)	0.91	(0.78 , 1.04)
2008006	1.79	(1.64 , 1.93)	1.81	(1.63 , 1.98)	0.85	(0.73 , 0.98)
2008240	1.62	(1.47 , 1.76)	1.60	(1.44 , 1.75)	0.87	(0.74 , 1.00)
2008103	1.67	(1.53 , 1.80)	1.66	(1.50 , 1.81)	0.94	(0.81 , 1.07)
2008148	1.79	(1.65 , 1.92)	1.82	(1.68 , 1.96)	0.93	(0.81 , 1.06)
2008236	1.96	(1.82 , 2.10)	1.88	(1.72 , 2.04)	0.88	(0.76 , 1.01)
2008259	1.65	(1.49 , 1.81)	1.63	(1.45 , 1.80)	0.94	(0.81 , 1.08)
2008191	1.91	(1.77 , 2.05)	1.98	(1.83 , 2.13)	0.85	(0.73 , 0.98)
2008242	1.81	(1.68 , 1.94)	1.85	(1.70 , 1.99)	0.92	(0.80 , 1.05)

Family	height to the cones 16 months		height to the cones 28 months		green cone weight 28 months *	
2008248	1.87	(1.72 , 2.01)	1.90	(1.75 , 2.06)	0.88	(0.76 , 1.01)
2008010	1.63	(1.49 , 1.77)	1.62	(1.47 , 1.77)	0.86	(0.74 , 0.99)
2008263	1.61	(1.47 , 1.75)	1.58	(1.43 , 1.73)	0.91	(0.79 , 1.05)
2008153	1.48	(1.34 , 1.61)	1.44	(1.30 , 1.58)	0.92	(0.80 , 1.05)
2008119	1.68	(1.54 , 1.81)	1.59	(1.44 , 1.74)	0.91	(0.79 , 1.05)
2008206	1.69	(1.56 , 1.82)	1.66	(1.51 , 1.81)	0.90	(0.77 , 1.03)
2008139	1.85	(1.72 , 1.98)	1.87	(1.73 , 2.01)	0.88	(0.75 , 1.01)
2008106	1.65	(1.51 , 1.80)	1.64	(1.49 , 1.80)	0.88	(0.76 , 1.01)
2008122	1.80	(1.64 , 1.95)	1.81	(1.64 , 1.99)	0.89	(0.76 , 1.02)
2008155	1.70	(1.56 , 1.85)	1.69	(1.54 , 1.85)	0.87	(0.75 , 1.00)
2008088	1.52	(1.38 , 1.66)	1.53	(1.37 , 1.69)	0.91	(0.78 , 1.04)
2008111	1.74	(1.57 , 1.90)	1.74	(1.55 , 1.92)	0.87	(0.75 , 1.00)
2008202	1.69	(1.54 , 1.83)	1.69	(1.54 , 1.85)	0.89	(0.77 , 1.03)
2008013	1.85	(1.70 , 2.00)	1.87	(1.71 , 2.03)	0.87	(0.75 , 1.00)
2008279	1.76	(1.60 , 1.92)	1.77	(1.59 , 1.94)	0.91	(0.79 , 1.05)
2008007	1.61	(1.46 , 1.75)	1.59	(1.43 , 1.74)	0.89	(0.77 , 1.02)
2008090	1.69	(1.55 , 1.83)	1.76	(1.60 , 1.92)	0.94	(0.81 , 1.07)
2008055	1.65	(1.51 , 1.78)	1.83	(1.67 , 2.00)	0.92	(0.80 , 1.06)
2008271	1.77	(1.63 , 1.91)	1.77	(1.62 , 1.93)	0.92	(0.80 , 1.06)
2008035	1.88	(1.74 , 2.03)	1.92	(1.76 , 2.08)	0.94	(0.81 , 1.07)
2008212	1.74	(1.59 , 1.88)	1.73	(1.57 , 1.89)	0.94	(0.81 , 1.07)
2008149	1.70	(1.56 , 1.85)	1.70	(1.54 , 1.86)	0.88	(0.76 , 1.01)
2008170	1.75	(1.60 , 1.89)	1.77	(1.62 , 1.93)	0.98	(0.85 , 1.12)
2008247	1.66	(1.51 , 1.80)	1.65	(1.49 , 1.81)	1.00	(0.87 , 1.14)
2008110	1.82	(1.66 , 1.97)	1.84	(1.67 , 2.01)	0.90	(0.78 , 1.04)
2008043	1.77	(1.64 , 1.90)	1.79	(1.65 , 1.93)	0.91	(0.79 , 1.05)
2008188	1.77	(1.62 , 1.92)	1.79	(1.62 , 1.95)	0.89	(0.76 , 1.02)
2008080	1.76	(1.60 , 1.92)	1.77	(1.59 , 1.94)	0.91	(0.79 , 1.05)
2008239	1.80	(1.66 , 1.94)	1.82	(1.67 , 1.97)	0.89	(0.77 , 1.03)
2008075	1.63	(1.49 , 1.76)	1.61	(1.45 , 1.76)	0.84	(0.72 , 0.97)
2008114	1.81	(1.67 , 1.96)	1.83	(1.67 , 1.98)	0.88	(0.75 , 1.01)
2008166	1.74	(1.59 , 1.88)	1.76	(1.59 , 1.92)	0.91	(0.78 , 1.04)
2008252	1.85	(1.72 , 1.99)	1.88	(1.74 , 2.03)	0.89	(0.77 , 1.02)
2008150	1.57	(1.43 , 1.71)	1.54	(1.39 , 1.69)	0.93	(0.80 , 1.06)
2008009	1.80	(1.67 , 1.94)	1.82	(1.67 , 1.96)	0.88	(0.76 , 1.01)
2008081	1.80	(1.64 , 1.96)	1.78	(1.58 , 1.97)	0.87	(0.74 , 1.00)

Family	height to the cones 16 months		height to the cones 28 months		green cone weight 28 months *	
2008041	1.67	(1.53 , 1.81)	1.67	(1.53 , 1.82)	0.89	(0.77 , 1.03)
2008061	1.61	(1.46 , 1.76)	1.58	(1.42 , 1.74)	0.95	(0.82 , 1.09)
2008087	1.61	(1.45 , 1.77)	1.58	(1.40 , 1.76)	0.89	(0.76 , 1.02)
2008147	1.63	(1.49 , 1.77)	1.66	(1.51 , 1.81)	0.92	(0.79 , 1.05)
2008258	1.58	(1.44 , 1.72)	1.63	(1.46 , 1.81)	0.89	(0.77 , 1.03)
2008130	1.87	(1.73 , 2.01)	1.93	(1.76 , 2.09)	0.90	(0.78 , 1.03)
2008100	1.68	(1.53 , 1.83)	1.68	(1.51 , 1.84)	0.92	(0.79 , 1.06)
2008132	1.66	(1.50 , 1.82)	1.65	(1.47 , 1.82)	0.90	(0.78 , 1.04)
2008256	1.79	(1.63 , 1.94)	1.78	(1.61 , 1.96)	0.93	(0.80 , 1.07)
2008093	1.86	(1.72 , 2.00)	1.89	(1.74 , 2.05)	0.88	(0.76 , 1.01)
2008108	1.73	(1.58 , 1.87)	1.70	(1.53 , 1.86)	0.91	(0.79 , 1.05)
2008179	1.97	(1.83 , 2.10)	2.00	(1.84 , 2.15)	0.88	(0.75 , 1.01)
2008192	1.61	(1.46 , 1.75)	1.58	(1.43 , 1.74)	0.90	(0.77 , 1.03)
2008089	1.57	(1.43 , 1.71)	1.53	(1.38 , 1.68)	0.88	(0.76 , 1.01)
2008064	1.69	(1.54 , 1.83)	1.68	(1.52 , 1.84)	0.89	(0.77 , 1.03)
2008269	1.73	(1.56 , 1.89)	1.74	(1.56 , 1.92)	0.90	(0.77 , 1.04)
2008059	1.76	(1.61 , 1.90)	1.76	(1.60 , 1.91)	0.86	(0.74 , 0.99)
2008047	1.78	(1.63 , 1.94)	1.79	(1.62 , 1.96)	0.89	(0.77 , 1.02)
2008194	1.77	(1.63 , 1.92)	1.78	(1.61 , 1.94)	0.88	(0.76 , 1.02)
2008232	1.76	(1.62 , 1.91)	1.78	(1.62 , 1.93)	0.89	(0.77 , 1.02)
2008173	2.09	(1.96 , 2.23)	2.25	(2.09 , 2.40)	0.88	(0.76 , 1.01)
2008120	1.80	(1.65 , 1.94)	1.81	(1.65 , 1.96)	0.89	(0.77 , 1.02)
2008262	1.66	(1.50 , 1.81)	1.65	(1.48 , 1.82)	0.89	(0.76 , 1.02)
2008187	1.72	(1.57 , 1.86)	1.65	(1.49 , 1.81)	0.90	(0.78 , 1.04)
2008051	1.67	(1.52 , 1.82)	1.66	(1.50 , 1.82)	0.90	(0.77 , 1.04)
2008198	1.58	(1.44 , 1.73)	1.55	(1.40 , 1.71)	0.97	(0.84 , 1.11)
2008054	1.78	(1.64 , 1.91)	1.75	(1.60 , 1.91)	0.89	(0.77 , 1.02)
2008244	1.71	(1.56 , 1.85)	1.71	(1.55 , 1.86)	0.88	(0.75 , 1.01)
2008177	1.86	(1.71 , 2.01)	1.80	(1.64 , 1.97)	0.86	(0.74 , 0.99)
2008117	1.76	(1.63 , 1.89)	1.94	(1.78 , 2.10)	0.90	(0.78 , 1.04)
2008016	1.86	(1.72 , 2.00)	1.89	(1.74 , 2.04)	0.88	(0.76 , 1.02)
2008267	1.72	(1.58 , 1.87)	1.72	(1.56 , 1.87)	0.90	(0.78 , 1.04)
2008040	1.82	(1.67 , 1.96)	1.85	(1.68 , 2.01)	0.86	(0.73 , 0.99)
2008218	1.58	(1.44 , 1.72)	1.72	(1.55 , 1.89)	0.93	(0.80 , 1.06)
2008070	1.68	(1.52 , 1.83)	1.69	(1.52 , 1.87)	0.91	(0.79 , 1.05)
2008102	1.79	(1.66 , 1.92)	1.74	(1.59 , 1.89)	0.88	(0.76 , 1.01)

Family	height to the cones 16 months		height to the cones 28 months		green cone weight 28 months *	
2008078	1.77	(1.62 , 1.93)	1.78	(1.62 , 1.95)	0.96	(0.83 , 1.09)
2008074	1.66	(1.52 , 1.80)	1.55	(1.38 , 1.73)	0.96	(0.84 , 1.10)

e.

Family	cohumulone 16 months *		cohumulone 28 months *		humulone + adhumulone 16 months *		humulone + adhumulone 28 months *		colupulone 16 months *		colupulone 28 months *		lupulone + adlupulone 16 months *		lupulone + adlupulone 28 months *	
2008003	2.95	(2.57 , 3.36)	2.56	(2.22 , 2.91)	6.59	(5.86 , 7.35)	5.22	(4.62 , 5.85)	2.35	(2.04 , 2.69)	2.58	(2.27 , 2.90)	2.03	(1.75 , 2.33)	2.12	(1.83 , 2.44)
2008190	2.03	(1.74 , 2.34)	2.08	(1.78 , 2.40)	5.30	(4.70 , 5.94)	4.80	(4.23 , 5.41)	1.94	(1.68 , 2.22)	2.29	(2.00 , 2.59)	1.87	(1.61 , 2.14)	2.12	(1.83 , 2.44)
2008209	3.09	(2.66 , 3.55)	2.56	(2.22 , 2.92)	7.24	(6.40 , 8.12)	5.39	(4.78 , 6.03)	2.16	(1.84 , 2.51)	2.49	(2.19 , 2.81)	2.04	(1.73 , 2.38)	2.11	(1.81 , 2.42)
2008151	2.91	(2.50 , 3.36)	2.41	(2.09 , 2.76)	7.06	(6.23 , 7.93)	5.65	(5.03 , 6.31)	2.40	(2.06 , 2.77)	2.51	(2.21 , 2.83)	2.24	(1.92 , 2.59)	2.38	(2.07 , 2.71)
2008039	2.79	(2.38 , 3.22)	2.45	(2.09 , 2.83)	5.59	(4.86 , 6.37)	5.36	(4.71 , 6.06)	1.95	(1.64 , 2.28)	2.42	(2.11 , 2.76)	1.60	(1.33 , 1.90)	2.07	(1.76 , 2.42)
2008152	2.76	(2.45 , 3.10)	2.09	(1.79 , 2.42)	7.30	(6.64 , 7.99)	4.73	(4.17 , 5.33)	2.56	(2.27 , 2.86)	2.47	(2.17 , 2.78)	2.28	(2.01 , 2.56)	2.11	(1.82 , 2.43)
2008001	2.73	(2.40 , 3.09)	2.54	(2.21 , 2.90)	6.26	(5.60 , 6.95)	5.73	(5.11 , 6.39)	2.13	(1.85 , 2.42)	2.39	(2.10 , 2.70)	1.88	(1.63 , 2.16)	2.07	(1.78 , 2.38)
2008073	2.03	(1.72 , 2.37)	2.29	(1.94 , 2.66)	4.86	(4.24 , 5.52)	4.86	(4.24 , 5.52)	1.97	(1.69 , 2.28)	2.36	(2.05 , 2.69)	1.79	(1.52 , 2.07)	1.91	(1.61 , 2.24)
2008168	2.58	(2.19 , 3.00)	2.11	(1.81 , 2.44)	6.84	(6.03 , 7.71)	4.72	(4.16 , 5.32)	2.59	(2.24 , 2.97)	2.47	(2.17 , 2.78)	2.59	(2.24 , 2.96)	2.15	(1.85 , 2.46)
2008161	2.63	(2.28 , 3.02)	2.23	(1.93 , 2.54)	7.03	(6.28 , 7.82)	5.24	(4.69 , 5.83)	2.11	(1.82 , 2.43)	2.51	(2.23 , 2.81)	2.09	(1.80 , 2.40)	2.34	(2.06 , 2.65)
2008290	2.59	(2.26 , 2.94)	2.38	(2.06 , 2.73)	7.01	(6.32 , 7.74)	5.46	(4.85 , 6.10)	2.00	(1.73 , 2.29)	2.27	(1.99 , 2.58)	2.01	(1.75 , 2.30)	2.01	(1.72 , 2.31)
2008208	2.63	(2.23 , 3.05)	2.22	(1.91 , 2.56)	6.79	(5.98 , 7.66)	5.27	(4.67 , 5.90)	2.10	(1.78 , 2.44)	2.37	(2.08 , 2.68)	2.06	(1.74 , 2.39)	2.20	(1.90 , 2.52)
2008251	2.46	(2.08 , 2.87)	2.17	(1.86 , 2.51)	6.31	(5.53 , 7.13)	5.05	(4.46 , 5.67)	2.31	(1.98 , 2.67)	2.36	(2.07 , 2.67)	2.34	(2.01 , 2.70)	2.21	(1.91 , 2.53)
2008052	2.19	(1.91 , 2.49)	2.32	(2.00 , 2.66)	5.43	(4.86 , 6.03)	4.81	(4.24 , 5.42)	1.80	(1.56 , 2.06)	2.16	(1.89 , 2.46)	1.70	(1.47 , 1.95)	1.74	(1.47 , 2.02)
2008037	1.87	(1.57 , 2.20)	2.12	(1.82 , 2.45)	5.36	(4.70 , 6.05)	5.15	(4.56 , 5.78)	1.76	(1.50 , 2.05)	2.16	(1.88 , 2.46)	1.82	(1.55 , 2.11)	1.98	(1.70 , 2.29)
2008154	3.53	(3.11 , 3.97)	3.13	(2.75 , 3.52)	6.60	(5.87 , 7.37)	5.51	(4.90 , 6.16)	2.60	(2.27 , 2.95)	2.71	(2.40 , 3.05)	1.99	(1.71 , 2.29)	1.99	(1.70 , 2.29)
2008136	2.84	(2.54 , 3.16)	2.47	(2.16 , 2.80)	7.27	(6.65 , 7.92)	5.39	(4.83 , 5.99)	2.31	(2.05 , 2.58)	2.45	(2.17 , 2.75)	2.36	(2.10 , 2.63)	2.15	(1.87 , 2.44)
2008096	2.27	(2.00 , 2.56)	2.31	(1.99 , 2.65)	5.11	(4.59 , 5.66)	4.89	(4.32 , 5.51)	1.97	(1.74 , 2.23)	2.27	(1.99 , 2.58)	1.84	(1.61 , 2.08)	1.90	(1.63 , 2.20)
2008071	2.80	(2.48 , 3.14)	2.27	(1.98 , 2.59)	6.78	(6.14 , 7.45)	5.01	(4.47 , 5.58)	2.13	(1.87 , 2.41)	2.23	(1.97 , 2.52)	2.14	(1.88 , 2.41)	1.98	(1.72 , 2.26)
2008124	2.64	(2.25 , 3.07)	2.35	(2.00 , 2.73)	6.52	(5.73 , 7.36)	5.01	(4.38 , 5.68)	2.27	(1.94 , 2.62)	2.33	(2.02 , 2.66)	2.21	(1.89 , 2.56)	1.95	(1.64 , 2.29)
2008145	3.17	(2.87 , 3.49)	2.57	(2.26 , 2.91)	7.43	(6.83 , 8.05)	5.68	(5.10 , 6.29)	2.56	(2.30 , 2.83)	2.50	(2.22 , 2.80)	2.39	(2.14 , 2.64)	2.17	(1.90 , 2.47)
2008084	2.20	(1.92 , 2.50)	2.47	(2.11 , 2.85)	5.93	(5.33 , 6.56)	5.39	(4.74 , 6.09)	2.17	(1.91 , 2.45)	2.59	(2.26 , 2.93)	2.21	(1.95 , 2.49)	2.32	(1.98 , 2.68)
2008083	2.72	(2.36 , 3.11)	2.61	(2.27 , 2.98)	7.09	(6.34 , 7.89)	5.86	(5.23 , 6.52)	2.25	(1.95 , 2.57)	2.34	(2.05 , 2.65)	2.30	(2.00 , 2.63)	2.03	(1.74 , 2.33)
2008056	2.55	(2.19 , 2.93)	2.30	(1.95 , 2.67)	6.02	(5.32 , 6.75)	5.06	(4.43 , 5.74)	2.49	(2.17 , 2.83)	2.54	(2.22 , 2.89)	2.29	(1.99 , 2.62)	2.33	(1.99 , 2.70)
2008261	2.93	(2.58 , 3.31)	2.49	(2.16 , 2.84)	6.62	(5.94 , 7.33)	5.29	(4.69 , 5.92)	2.32	(2.03 , 2.63)	2.40	(2.11 , 2.71)	2.10	(1.83 , 2.39)	2.03	(1.74 , 2.34)

Family	cohumulone 16 months *		cohumulone 28 months *		humulone + adhumulone 16 months *		humulone + adhumulone 28 months *		colupulone 16 months *		colupulone 28 months *		lupulone + adlupulone 16 months *		lupulone + adlupulone 28 months *	
2008157	2.61	(2.22 , 3.03)	2.21	(1.89 , 2.54)	6.24	(5.46 , 7.06)	4.73	(4.17 , 5.33)	2.16	(1.84 , 2.51)	2.27	(1.99 , 2.58)	2.07	(1.75 , 2.40)	1.90	(1.62 , 2.20)
2008006	2.29	(1.96 , 2.65)	2.74	(2.39 , 3.11)	6.06	(5.36 , 6.79)	5.63	(5.01 , 6.28)	2.08	(1.79 , 2.40)	2.47	(2.18 , 2.79)	2.38	(2.07 , 2.71)	2.08	(1.79 , 2.40)
2008240	2.65	(2.34 , 2.97)	2.72	(2.38 , 3.09)	5.88	(5.28 , 6.50)	5.83	(5.20 , 6.50)	2.57	(2.28 , 2.87)	2.46	(2.16 , 2.77)	2.08	(1.83 , 2.35)	2.13	(1.83 , 2.44)
2008103	2.29	(1.93 , 2.69)	2.61	(2.29 , 2.95)	6.35	(5.57 , 7.18)	5.69	(5.11 , 6.30)	2.01	(1.70 , 2.35)	2.57	(2.29 , 2.88)	2.14	(1.82 , 2.48)	2.35	(2.07 , 2.66)
2008148	2.65	(2.34 , 2.98)	2.54	(2.22 , 2.87)	6.18	(5.57 , 6.82)	5.18	(4.63 , 5.77)	2.28	(2.01 , 2.57)	2.49	(2.21 , 2.79)	2.08	(1.82 , 2.35)	2.05	(1.78 , 2.33)
2008236	2.90	(2.60 , 3.22)	2.38	(2.05 , 2.72)	5.04	(4.52 , 5.58)	4.49	(3.94 , 5.08)	2.20	(1.95 , 2.46)	2.36	(2.07 , 2.67)	1.51	(1.31 , 1.73)	1.72	(1.46 , 2.00)
2008259	2.48	(2.10 , 2.89)	2.39	(2.04 , 2.77)	6.02	(5.26 , 6.83)	5.22	(4.58 , 5.90)	2.11	(1.80 , 2.46)	2.34	(2.03 , 2.67)	1.95	(1.65 , 2.28)	1.99	(1.68 , 2.33)
2008191	3.05	(2.70 , 3.43)	2.58	(2.27 , 2.92)	4.85	(4.27 , 5.46)	4.37	(3.86 , 4.91)	2.36	(2.07 , 2.67)	2.51	(2.23 , 2.81)	1.59	(1.36 , 1.85)	1.70	(1.46 , 1.96)
2008242	2.35	(2.04 , 2.68)	1.99	(1.71 , 2.28)	5.13	(4.54 , 5.75)	4.55	(4.03 , 5.10)	2.61	(2.30 , 2.93)	2.34	(2.07 , 2.63)	2.24	(1.96 , 2.53)	2.11	(1.84 , 2.40)
2008248	1.63	(1.37 , 1.91)	2.04	(1.74 , 2.36)	4.58	(4.02 , 5.17)	4.94	(4.37 , 5.56)	1.76	(1.51 , 2.03)	2.18	(1.90 , 2.48)	1.90	(1.64 , 2.17)	2.08	(1.79 , 2.39)
2008010	2.58	(2.25 , 2.92)	2.54	(2.21 , 2.90)	6.32	(5.66 , 7.01)	5.13	(4.54 , 5.75)	1.91	(1.65 , 2.19)	2.37	(2.08 , 2.68)	1.90	(1.64 , 2.17)	1.99	(1.71 , 2.29)
2008263	2.58	(2.25 , 2.92)	2.54	(2.21 , 2.90)	6.62	(5.95 , 7.33)	5.36	(4.76 , 6.00)	2.26	(1.98 , 2.57)	2.50	(2.21 , 2.82)	2.23	(1.95 , 2.53)	2.18	(1.88 , 2.50)
2008153	2.70	(2.40 , 3.01)	2.39	(2.09 , 2.71)	6.76	(6.16 , 7.38)	5.26	(4.71 , 5.85)	2.08	(1.84 , 2.34)	2.37	(2.09 , 2.66)	2.04	(1.80 , 2.29)	2.08	(1.81 , 2.37)
2008119	2.44	(2.10 , 2.81)	2.31	(1.99 , 2.65)	5.77	(5.10 , 6.49)	4.94	(4.36 , 5.55)	1.96	(1.68 , 2.26)	2.28	(2.00 , 2.59)	1.85	(1.58 , 2.14)	1.92	(1.64 , 2.22)
2008206	3.02	(2.66 , 3.39)	2.63	(2.31 , 2.97)	5.87	(5.23 , 6.54)	5.31	(4.75 , 5.90)	2.31	(2.02 , 2.62)	2.55	(2.26 , 2.85)	1.85	(1.60 , 2.12)	2.05	(1.79 , 2.34)
2008139	1.82	(1.57 , 2.10)	2.02	(1.73 , 2.35)	4.70	(4.17 , 5.25)	4.55	(4.00 , 5.14)	2.39	(2.11 , 2.68)	2.31	(2.03 , 2.62)	2.41	(2.14 , 2.70)	2.11	(1.82 , 2.42)
2008106	2.42	(2.11 , 2.76)	2.33	(2.01 , 2.68)	5.77	(5.15 , 6.44)	4.98	(4.40 , 5.59)	1.90	(1.64 , 2.17)	2.51	(2.21 , 2.82)	1.84	(1.59 , 2.11)	2.27	(1.97 , 2.60)
2008122	2.38	(2.00 , 2.78)	2.55	(2.21 , 2.91)	5.40	(4.68 , 6.17)	5.49	(4.88 , 6.13)	2.35	(2.01 , 2.71)	2.61	(2.30 , 2.93)	2.15	(1.84 , 2.50)	2.26	(1.95 , 2.58)
2008155	2.95	(2.60 , 3.32)	2.60	(2.26 , 2.96)	5.18	(4.59 , 5.81)	5.37	(4.77 , 6.01)	2.31	(2.03 , 2.62)	2.61	(2.30 , 2.93)	1.60	(1.37 , 1.85)	2.16	(1.86 , 2.47)
2008088	2.70	(2.36 , 3.05)	2.81	(2.46 , 3.19)	6.66	(5.99 , 7.37)	5.94	(5.30 , 6.61)	2.31	(2.02 , 2.62)	2.65	(2.34 , 2.97)	2.17	(1.90 , 2.46)	2.38	(2.07 , 2.71)
2008111	2.31	(1.94 , 2.71)	2.34	(1.99 , 2.72)	6.18	(5.41 , 7.01)	5.27	(4.63 , 5.96)	2.18	(1.86 , 2.53)	2.52	(2.20 , 2.87)	2.18	(1.86 , 2.53)	2.28	(1.95 , 2.64)
2008202	3.18	(2.86 , 3.52)	2.64	(2.30 , 3.01)	6.28	(5.70 , 6.89)	5.17	(4.57 , 5.79)	2.19	(1.94 , 2.46)	2.51	(2.21 , 2.83)	1.81	(1.59 , 2.05)	1.92	(1.64 , 2.22)
2008013	2.03	(1.75 , 2.34)	2.13	(1.83 , 2.46)	5.93	(5.30 , 6.60)	4.98	(4.40 , 5.59)	1.96	(1.69 , 2.24)	2.24	(1.96 , 2.54)	2.14	(1.87 , 2.43)	2.06	(1.77 , 2.37)
2008279	3.38	(2.93 , 3.86)	2.43	(2.07 , 2.81)	7.00	(6.18 , 7.87)	4.92	(4.29 , 5.58)	2.57	(2.22 , 2.95)	2.37	(2.06 , 2.71)	2.15	(1.83 , 2.49)	1.89	(1.59 , 2.22)
2008007	2.97	(2.59 , 3.37)	2.62	(2.28 , 2.99)	6.57	(5.84 , 7.34)	5.43	(4.83 , 6.07)	2.10	(1.81 , 2.42)	2.28	(1.99 , 2.58)	1.88	(1.61 , 2.18)	1.90	(1.62 , 2.20)
2008090	3.15	(2.82 , 3.51)	2.75	(2.42 , 3.09)	6.73	(6.10 , 7.40)	5.74	(5.15 , 6.35)	2.18	(1.91 , 2.46)	2.39	(2.12 , 2.69)	1.99	(1.75 , 2.26)	2.04	(1.78 , 2.33)
2008055	2.40	(2.11 , 2.71)	2.22	(1.91 , 2.56)	6.27	(5.66 , 6.92)	4.95	(4.37 , 5.56)	2.44	(2.16 , 2.74)	2.48	(2.19 , 2.80)	2.54	(2.26 , 2.84)	2.22	(1.92 , 2.55)
2008271	2.67	(2.36 , 3.00)	2.49	(2.16 , 2.85)	6.33	(5.71 , 6.97)	5.16	(4.57 , 5.79)	2.04	(1.79 , 2.31)	2.28	(1.99 , 2.58)	1.80	(1.57 , 2.05)	1.85	(1.58 , 2.15)
2008035	2.32	(2.01 , 2.65)	2.38	(2.05 , 2.73)	6.01	(5.37 , 6.69)	5.33	(4.73 , 5.97)	2.05	(1.78 , 2.35)	2.41	(2.12 , 2.73)	2.09	(1.82 , 2.38)	2.13	(1.84 , 2.45)
2008212	2.38	(2.04 , 2.75)	2.56	(2.20 , 2.96)	5.75	(5.07 , 6.47)	5.40	(4.74 , 6.09)	2.63	(2.30 , 2.98)	2.63	(2.30 , 2.99)	2.43	(2.12 , 2.77)	2.25	(1.92 , 2.61)
2008149	3.93	(3.53 , 4.36)	2.41	(2.08 , 2.76)	6.87	(6.18 , 7.59)	4.98	(4.40 , 5.60)	2.86	(2.54 , 3.20)	2.44	(2.14 , 2.75)	2.19	(1.92 , 2.49)	2.04	(1.75 , 2.35)
2008170	2.30	(2.01 , 2.61)	2.45	(2.12 , 2.80)	6.31	(5.69 , 6.95)	5.38	(4.78 , 6.02)	1.94	(1.69 , 2.21)	2.19	(1.91 , 2.49)	2.03	(1.78 , 2.29)	1.86	(1.59 , 2.16)
2008247	2.31	(1.95 , 2.71)	2.73	(2.38 , 3.10)	6.11	(5.35 , 6.93)	5.46	(4.85 , 6.10)	1.93	(1.63 , 2.26)	2.40	(2.10 , 2.71)	1.89	(1.59 , 2.21)	1.90	(1.62 , 2.20)
2008110	2.84	(2.43 , 3.28)	2.41	(2.05 , 2.79)	6.17	(5.40 , 6.99)	5.03	(4.40 , 5.70)	2.30	(1.96 , 2.65)	2.51	(2.19 , 2.86)	2.00	(1.70 , 2.34)	2.09	(1.77 , 2.43)
2008043	1.85	(1.62 , 2.09)	2.16	(1.87 , 2.46)	5.03	(4.54 , 5.54)	4.61	(4.09 , 5.16)	2.01	(1.78 , 2.26)	2.14	(1.88 , 2.42)	2.01	(1.79 , 2.25)	1.78	(1.53 , 2.05)

Family	**cohumulone** 16 months *		**cohumulone** 28 months *		**humulone + adhumulone** 16 months *		**humulone + adhumulone** 28 months *		**colupulone** 16 months *		**colupulone** 28 months *		**lupulone + adlupulone** 16 months *		**lupulone + adlupulone** 28 months *	
2008188	2.57	(2.19 , 3.00)	2.43	(2.07 , 2.81)	5.93	(5.18 , 6.74)	4.86	(4.24 , 5.52)	2.54	(2.19 , 2.92)	2.60	(2.28 , 2.95)	2.18	(1.86 , 2.53)	2.07	(1.75 , 2.41)
2008080	2.21	(1.85 , 2.60)	2.28	(1.93 , 2.65)	5.94	(5.19 , 6.74)	5.01	(4.38 , 5.68)	2.18	(1.86 , 2.53)	2.29	(1.98 , 2.62)	2.34	(2.01 , 2.70)	1.97	(1.66 , 2.31)
2008239	2.50	(2.12 , 2.91)	2.49	(2.16 , 2.85)	5.52	(4.79 , 6.30)	5.11	(4.53 , 5.74)	2.50	(2.15 , 2.87)	2.78	(2.46 , 3.11)	2.11	(1.80 , 2.45)	2.29	(1.98 , 2.62)
2008075	2.77	(2.43 , 3.13)	2.27	(1.97 , 2.58)	7.58	(6.86 , 8.34)	5.12	(4.57 , 5.70)	2.34	(2.05 , 2.65)	2.45	(2.17 , 2.74)	2.49	(2.20 , 2.81)	2.28	(2.00 , 2.58)
2008114	2.12	(1.79 , 2.46)	2.01	(1.72 , 2.34)	6.40	(5.69 , 7.16)	4.82	(4.25 , 5.42)	2.04	(1.75 , 2.35)	2.42	(2.12 , 2.73)	2.36	(2.05 , 2.69)	2.46	(2.15 , 2.80)
2008166	3.09	(2.70 , 3.50)	2.27	(1.95 , 2.61)	5.35	(4.70 , 6.04)	4.67	(4.11 , 5.27)	2.18	(1.88 , 2.50)	2.18	(1.90 , 2.48)	1.60	(1.35 , 1.87)	1.70	(1.44 , 1.98)
2008252	2.51	(2.16 , 2.88)	2.17	(1.86 , 2.50)	6.37	(5.65 , 7.12)	5.22	(4.62 , 5.85)	2.09	(1.80 , 2.40)	2.31	(2.02 , 2.61)	2.07	(1.78 , 2.38)	2.20	(1.90 , 2.52)
2008150	2.97	(2.68 , 3.28)	2.45	(2.09 , 2.83)	6.54	(5.98 , 7.12)	5.08	(4.45 , 5.76)	2.01	(1.78 , 2.25)	2.40	(2.09 , 2.74)	1.69	(1.48 , 1.91)	2.00	(1.69 , 2.34)
2008009	2.61	(2.28 , 2.96)	2.39	(2.08 , 2.71)	7.51	(6.79 , 8.26)	5.81	(5.22 , 6.43)	1.83	(1.57 , 2.10)	2.11	(1.85 , 2.39)	1.97	(1.71 , 2.25)	1.90	(1.65 , 2.18)
2008081	3.21	(2.77 , 3.68)	2.72	(2.34 , 3.12)	8.22	(7.33 , 9.16)	6.19	(5.49 , 6.93)	2.58	(2.22 , 2.96)	2.59	(2.26 , 2.93)	2.54	(2.19 , 2.91)	2.43	(2.08 , 2.80)
2008041	3.54	(3.19 , 3.92)	2.41	(2.11 , 2.74)	6.61	(5.98 , 7.27)	5.66	(5.08 , 6.27)	2.45	(2.17 , 2.74)	2.29	(2.02 , 2.58)	1.73	(1.50 , 1.97)	1.98	(1.72 , 2.26)
2008061	2.22	(1.94 , 2.53)	2.36	(2.01 , 2.74)	5.81	(5.22 , 6.43)	5.20	(4.56 , 5.89)	2.01	(1.76 , 2.28)	2.37	(2.06 , 2.71)	2.00	(1.75 , 2.27)	2.01	(1.70 , 2.35)
2008087	2.73	(2.33 , 3.16)	2.68	(2.31 , 3.08)	6.41	(5.62 , 7.24)	5.27	(4.62 , 5.95)	2.15	(1.83 , 2.50)	2.64	(2.31 , 2.99)	2.02	(1.71 , 2.35)	2.22	(1.89 , 2.57)
2008147	3.31	(3.00 , 3.64)	2.74	(2.41 , 3.09)	7.30	(6.71 , 7.92)	5.95	(5.36 , 6.58)	2.18	(1.94 , 2.43)	2.61	(2.32 , 2.92)	1.94	(1.72 , 2.17)	2.25	(1.97 , 2.55)
2008258	2.82	(2.51 , 3.13)	2.43	(2.10 , 2.78)	7.89	(7.25 , 8.57)	5.49	(4.88 , 6.14)	3.12	(2.82 , 3.44)	3.17	(2.84 , 3.53)	3.35	(3.05 , 3.67)	3.09	(2.73 , 3.47)
2008130	2.62	(2.29 , 2.97)	2.74	(2.39 , 3.11)	6.45	(5.79 , 7.15)	5.71	(5.08 , 6.36)	1.97	(1.71 , 2.25)	2.38	(2.08 , 2.69)	1.92	(1.67 , 2.20)	1.98	(1.70 , 2.29)
2008100	2.61	(2.28 , 2.96)	2.53	(2.17 , 2.92)	7.12	(6.42 , 7.86)	5.58	(4.91 , 6.28)	2.14	(1.87 , 2.44)	2.71	(2.38 , 3.07)	2.28	(2.00 , 2.58)	2.51	(2.16 , 2.88)
2008132	2.99	(2.60 , 3.40)	2.27	(1.92 , 2.64)	7.07	(6.32 , 7.87)	4.93	(4.31 , 5.59)	2.47	(2.15 , 2.81)	2.45	(2.13 , 2.79)	2.28	(1.98 , 2.60)	2.12	(1.80 , 2.47)
2008256	2.89	(2.51 , 3.30)	2.14	(1.81 , 2.50)	6.66	(5.93 , 7.43)	4.64	(4.04 , 5.29)	3.31	(2.94 , 3.70)	3.01	(2.66 , 3.39)	2.91	(2.56 , 3.27)	2.77	(2.40 , 3.16)
2008093	2.24	(1.97 , 2.53)	2.17	(1.88 , 2.48)	5.45	(4.92 , 6.02)	4.70	(4.18 , 5.26)	1.75	(1.52 , 1.99)	2.18	(1.92 , 2.46)	1.65	(1.44 , 1.88)	1.90	(1.65 , 2.18)
2008108	2.26	(1.99 , 2.55)	2.14	(1.83 , 2.47)	5.54	(5.00 , 6.11)	4.71	(4.15 , 5.31)	2.28	(2.02 , 2.55)	2.36	(2.07 , 2.67)	2.31	(2.06 , 2.58)	2.17	(1.87 , 2.48)
2008179	2.13	(1.85 , 2.42)	2.29	(1.97 , 2.63)	6.57	(5.94 , 7.23)	5.52	(4.91 , 6.17)	2.23	(1.96 , 2.51)	2.47	(2.17 , 2.78)	2.52	(2.24 , 2.82)	2.36	(2.05 , 2.70)
2008192	3.13	(2.74 , 3.55)	2.20	(1.87 , 2.57)	5.82	(5.14 , 6.54)	4.77	(4.16 , 5.42)	3.38	(3.00 , 3.77)	2.48	(2.16 , 2.82)	2.43	(2.12 , 2.76)	2.09	(1.77 , 2.43)
2008089	2.90	(2.53 , 3.30)	2.46	(2.13 , 2.81)	6.97	(6.22 , 7.76)	5.27	(4.67 , 5.90)	2.25	(1.94 , 2.57)	2.35	(2.06 , 2.66)	2.17	(1.88 , 2.49)	2.04	(1.75 , 2.35)
2008064	2.66	(2.27 , 3.09)	2.35	(2.00 , 2.72)	6.52	(5.73 , 7.37)	5.28	(4.64 , 5.97)	2.02	(1.71 , 2.36)	2.34	(2.03 , 2.67)	1.89	(1.60 , 2.22)	2.08	(1.76 , 2.42)
2008269	2.81	(2.40 , 3.25)	2.40	(2.01 , 2.82)	5.30	(4.59 , 6.07)	5.18	(4.48 , 5.93)	2.42	(2.08 , 2.79)	2.43	(2.09 , 2.80)	1.91	(1.61 , 2.24)	2.10	(1.74 , 2.49)
2008059	2.73	(2.32 , 3.17)	2.38	(2.05 , 2.72)	6.47	(5.68 , 7.31)	4.98	(4.40 , 5.59)	2.30	(1.96 , 2.66)	2.61	(2.30 , 2.93)	2.12	(1.81 , 2.47)	2.25	(1.94 , 2.57)
2008047	2.78	(2.41 , 3.18)	2.46	(2.13 , 2.81)	5.90	(5.21 , 6.63)	4.81	(4.24 , 5.41)	2.21	(1.90 , 2.53)	2.27	(1.99 , 2.57)	1.82	(1.55 , 2.11)	1.70	(1.43 , 1.98)
2008194	3.27	(2.87 , 3.70)	2.27	(1.95 , 2.61)	7.70	(6.91 , 8.52)	5.20	(4.60 , 5.82)	2.75	(2.42 , 3.11)	2.47	(2.17 , 2.79)	2.55	(2.23 , 2.89)	2.25	(1.95 , 2.57)
2008232	2.06	(1.74 , 2.40)	2.03	(1.73 , 2.35)	5.58	(4.91 , 6.28)	4.64	(4.08 , 5.23)	2.26	(1.96 , 2.59)	2.61	(2.30 , 2.93)	2.36	(2.05 , 2.69)	2.45	(2.14 , 2.79)
2008173	2.17	(1.81 , 2.56)	2.33	(2.01 , 2.68)	6.28	(5.50 , 7.11)	5.12	(4.53 , 5.75)	2.20	(1.87 , 2.55)	2.39	(2.10 , 2.70)	2.45	(2.11 , 2.82)	2.14	(1.84 , 2.45)
2008120	2.29	(1.95 , 2.65)	2.47	(2.14 , 2.83)	6.36	(5.65 , 7.12)	5.69	(5.07 , 6.35)	2.45	(2.13 , 2.79)	2.72	(2.40 , 3.05)	2.20	(1.91 , 2.52)	2.38	(2.07 , 2.72)
2008262	2.67	(2.31 , 3.05)	2.31	(1.96 , 2.68)	6.42	(5.70 , 7.18)	5.10	(4.46 , 5.77)	2.17	(1.87 , 2.49)	2.40	(2.09 , 2.74)	1.96	(1.68 , 2.26)	2.11	(1.79 , 2.46)
2008187	2.16	(1.84 , 2.51)	2.11	(1.80 , 2.43)	5.52	(4.86 , 6.23)	4.80	(4.23 , 5.40)	1.76	(1.49 , 2.05)	2.16	(1.88 , 2.46)	1.81	(1.54 , 2.10)	1.89	(1.61 , 2.19)
2008051	3.11	(2.72 , 3.53)	2.54	(2.18 , 2.93)	6.54	(5.82 , 7.30)	5.27	(4.62 , 5.95)	2.75	(2.42 , 3.11)	2.58	(2.25 , 2.93)	2.25	(1.95 , 2.57)	2.09	(1.77 , 2.44)

Family	cohumulone 16 months *		cohumulone 28 months *		humulone + adhumulone 16 months *		humulone + adhumulone 28 months *		colupulone 16 months *		colupulone 28 months *		lupulone + adlupulone 16 months *		lupulone + adlupulone 28 months *	
2008198	2.36	(2.03 , 2.73)	2.23	(1.92 , 2.57)	5.38	(4.73 , 6.08)	4.74	(4.17 , 5.34)	2.04	(1.75 , 2.35)	2.19	(1.91 , 2.49)	1.74	(1.48 , 2.03)	1.78	(1.51 , 2.07)
2008054	1.89	(1.65 , 2.15)	2.20	(1.91 , 2.51)	4.87	(4.36 , 5.41)	4.68	(4.15 , 5.23)	1.60	(1.39 , 1.83)	2.36	(2.09 , 2.65)	1.57	(1.36 , 1.79)	2.01	(1.75 , 2.30)
2008244	2.64	(2.33 , 2.97)	2.49	(2.16 , 2.85)	6.81	(6.18 , 7.48)	5.38	(4.78 , 6.02)	2.26	(1.99 , 2.55)	2.66	(2.35 , 2.99)	2.05	(1.80 , 2.32)	2.22	(1.92 , 2.54)
2008177	2.66	(2.26 , 3.09)	2.13	(1.82 , 2.46)	6.41	(5.62 , 7.24)	4.98	(4.40 , 5.60)	2.20	(1.88 , 2.56)	2.45	(2.15 , 2.76)	2.08	(1.76 , 2.42)	2.41	(2.10 , 2.75)
2008117	3.07	(2.71 , 3.45)	2.70	(2.38 , 3.04)	5.84	(5.21 , 6.51)	5.01	(4.46 , 5.58)	2.26	(1.98 , 2.57)	2.52	(2.24 , 2.82)	1.68	(1.44 , 1.94)	1.84	(1.59 , 2.11)
2008016	2.14	(1.88 , 2.42)	2.59	(2.25 , 2.95)	5.79	(5.24 , 6.38)	5.61	(4.99 , 6.26)	2.29	(2.03 , 2.56)	2.60	(2.29 , 2.92)	2.46	(2.20 , 2.73)	2.29	(1.99 , 2.62)
2008267	2.41	(2.07 , 2.78)	2.53	(2.19 , 2.89)	6.83	(6.09 , 7.61)	5.29	(4.69 , 5.92)	2.06	(1.77 , 2.37)	2.32	(2.03 , 2.63)	2.13	(1.84 , 2.44)	1.91	(1.63 , 2.21)
2008040	3.20	(2.76 , 3.67)	2.41	(2.06 , 2.79)	7.26	(6.42 , 8.15)	5.02	(4.39 , 5.69)	2.38	(2.04 , 2.75)	2.28	(1.97 , 2.61)	2.14	(1.82 , 2.48)	1.87	(1.57 , 2.20)
2008218	2.95	(2.62 , 3.29)	2.70	(2.36 , 3.07)	7.94	(7.25 , 8.66)	5.94	(5.30 , 6.61)	2.22	(1.96 , 2.51)	2.39	(2.10 , 2.70)	2.23	(1.97 , 2.51)	2.06	(1.77 , 2.37)
2008070	2.64	(2.28 , 3.02)	1.94	(1.62 , 2.28)	6.38	(5.67 , 7.14)	5.11	(4.47 , 5.78)	2.53	(2.20 , 2.87)	2.03	(1.74 , 2.34)	2.50	(2.18 , 2.84)	2.05	(1.74 , 2.39)
2008102	3.29	(2.85 , 3.76)	2.47	(2.14 , 2.83)	7.20	(6.36 , 8.08)	5.23	(4.64 , 5.86)	2.50	(2.15 , 2.88)	2.52	(2.22 , 2.84)	2.29	(1.96 , 2.64)	2.19	(1.89 , 2.51)
2008078	2.93	(2.51 , 3.38)	2.43	(2.07 , 2.81)	7.58	(6.72 , 8.48)	4.97	(4.34 , 5.64)	2.41	(2.07 , 2.78)	2.57	(2.24 , 2.91)	2.38	(2.04 , 2.74)	2.07	(1.75 , 2.41)
2008074	2.52	(2.22 , 2.84)	2.59	(2.27 , 2.92)	6.47	(5.85 , 7.13)	5.87	(5.28 , 6.49)	1.94	(1.69 , 2.20)	2.27	(2.00 , 2.55)	2.04	(1.79 , 2.31)	2.10	(1.83 , 2.39)

f.

Family	α-acid 16 months		α-acid 28 months *		β-acid 16 months *		β-acid 28 months *	
2008003	9.74	(8.76 , 10.72)	7.78	(6.94 , 8.66)	4.40	(3.86 , 4.98)	4.71	(4.17 , 5.29)
2008190	7.50	(6.60 , 8.41)	6.97	(6.18 , 7.82)	3.85	(3.37 , 4.36)	4.42	(3.89 , 4.98)
2008209	10.58	(9.50 , 11.66)	7.95	(7.10 , 8.85)	4.22	(3.65 , 4.84)	4.62	(4.08 , 5.19)
2008151	10.24	(9.16 , 11.32)	8.06	(7.21 , 8.97)	4.65	(4.04 , 5.29)	4.86	(4.31 , 5.44)
2008039	8.58	(7.50 , 9.66)	7.82	(6.92 , 8.78)	3.62	(3.09 , 4.19)	4.52	(3.95 , 5.13)
2008152	10.29	(9.45 , 11.13)	6.92	(6.13 , 7.76)	4.84	(4.33 , 5.37)	4.59	(4.05 , 5.16)
2008001	9.19	(8.29 , 10.09)	8.24	(7.38 , 9.15)	4.05	(3.57 , 4.57)	4.48	(3.95 , 5.04)
2008073	7.17	(6.19 , 8.15)	7.22	(6.35 , 8.14)	3.80	(3.30 , 4.34)	4.33	(3.77 , 4.92)
2008168	9.70	(8.62 , 10.78)	6.92	(6.13 , 7.76)	5.15	(4.51 , 5.83)	4.62	(4.08 , 5.19)
2008161	9.82	(8.85 , 10.80)	7.51	(6.74 , 8.33)	4.22	(3.69 , 4.79)	4.82	(4.30 , 5.36)
2008290	9.75	(8.85 , 10.66)	7.86	(7.01 , 8.75)	4.04	(3.55 , 4.56)	4.31	(3.79 , 4.86)
2008208	9.60	(8.52 , 10.68)	7.54	(6.72 , 8.41)	4.18	(3.61 , 4.80)	4.56	(4.03 , 5.13)
2008251	8.92	(7.84 , 10.00)	7.28	(6.47 , 8.14)	4.65	(4.05 , 5.30)	4.56	(4.03 , 5.13)
2008052	7.85	(7.00 , 8.69)	7.19	(6.39 , 8.04)	3.55	(3.12 , 4.01)	3.98	(3.48 , 4.51)

Family	α-acid 16 months		α-acid 28 months *		β-acid 16 months *		β-acid 28 months *	
2008037	7.56	(6.58 , 8.54)	7.35	(6.53 , 8.21)	3.64	(3.14 , 4.16)	4.18	(3.67 , 4.72)
2008154	10.38	(9.40 , 11.36)	8.58	(7.70 , 9.51)	4.64	(4.08 , 5.23)	4.76	(4.21 , 5.34)
2008136	10.32	(9.53 , 11.11)	7.87	(7.08 , 8.70)	4.67	(4.20 , 5.17)	4.61	(4.10 , 5.14)
2008096	7.55	(6.76 , 8.35)	7.26	(6.45 , 8.12)	3.85	(3.42 , 4.30)	4.23	(3.71 , 4.77)
2008071	9.74	(8.89 , 10.58)	7.33	(6.56 , 8.13)	4.29	(3.81 , 4.79)	4.24	(3.76 , 4.75)
2008124	9.32	(8.24 , 10.40)	7.41	(6.53 , 8.34)	4.48	(3.89 , 5.12)	4.33	(3.77 , 4.93)
2008145	10.75	(10.00 , 11.50)	8.22	(7.41 , 9.08)	4.95	(4.49 , 5.43)	4.67	(4.17 , 5.21)
2008084	8.26	(7.42 , 9.10)	7.87	(6.96 , 8.83)	4.39	(3.91 , 4.90)	4.88	(4.28 , 5.51)
2008083	10.02	(9.04 , 11.00)	8.42	(7.55 , 9.34)	4.56	(4.01 , 5.15)	4.39	(3.87 , 4.95)
2008056	8.73	(7.75 , 9.71)	7.42	(6.54 , 8.35)	4.79	(4.22 , 5.40)	4.84	(4.25 , 5.47)
2008261	9.65	(8.75 , 10.56)	7.78	(6.94 , 8.67)	4.44	(3.93 , 4.99)	4.46	(3.93 , 5.02)
2008157	9.01	(7.93 , 10.09)	7.02	(6.22 , 7.86)	4.25	(3.67 , 4.87)	4.22	(3.71 , 4.77)
2008006	8.56	(7.58 , 9.54)	8.32	(7.46 , 9.24)	4.48	(3.93 , 5.06)	4.58	(4.05 , 5.15)
2008240	8.82	(7.98 , 9.66)	8.50	(7.63 , 9.43)	4.69	(4.20 , 5.21)	4.60	(4.06 , 5.17)
2008103	8.85	(7.77 , 9.93)	8.28	(7.47 , 9.14)	4.18	(3.61 , 4.79)	4.91	(4.39 , 5.46)
2008148	9.18	(8.33 , 10.02)	7.73	(6.95 , 8.56)	4.39	(3.91 , 4.90)	4.57	(4.07 , 5.10)
2008236	8.20	(7.41 , 8.99)	6.99	(6.20 , 7.83)	3.79	(3.36 , 4.23)	4.18	(3.67 , 4.73)
2008259	8.68	(7.60 , 9.76)	7.64	(6.75 , 8.59)	4.10	(3.53 , 4.70)	4.37	(3.81 , 4.97)
2008191	8.20	(7.29 , 9.10)	7.06	(6.31 , 7.85)	4.02	(3.54 , 4.54)	4.32	(3.84 , 4.84)
2008242	7.67	(6.76 , 8.57)	6.64	(5.92 , 7.41)	4.87	(4.33 , 5.43)	4.47	(3.97 , 4.99)
2008248	6.57	(5.66 , 7.47)	7.08	(6.28 , 7.92)	3.71	(3.24 , 4.20)	4.27	(3.76 , 4.82)
2008010	9.10	(8.19 , 10.00)	7.69	(6.86 , 8.57)	3.85	(3.38 , 4.36)	4.40	(3.88 , 4.96)
2008263	9.36	(8.45 , 10.26)	7.90	(7.05 , 8.79)	4.50	(3.99 , 5.05)	4.69	(4.15 , 5.26)
2008153	9.62	(8.82 , 10.41)	7.68	(6.89 , 8.50)	4.14	(3.70 , 4.61)	4.47	(3.97 , 4.99)
2008119	8.44	(7.46 , 9.42)	7.30	(6.49 , 8.16)	3.85	(3.34 , 4.39)	4.24	(3.73 , 4.79)
2008206	9.08	(8.17 , 9.98)	7.94	(7.14 , 8.78)	4.20	(3.70 , 4.73)	4.62	(4.11 , 5.15)
2008139	6.74	(5.89 , 7.58)	6.69	(5.92 , 7.52)	4.79	(4.29 , 5.33)	4.43	(3.91 , 4.99)
2008106	8.32	(7.42 , 9.23)	7.37	(6.55 , 8.23)	3.77	(3.30 , 4.27)	4.76	(4.22 , 5.34)
2008122	7.96	(6.88 , 9.04)	8.02	(7.17 , 8.92)	4.51	(3.92 , 5.15)	4.85	(4.30 , 5.44)
2008155	8.41	(7.50 , 9.31)	7.96	(7.12 , 8.86)	3.98	(3.50 , 4.49)	4.77	(4.22 , 5.35)
2008088	9.55	(8.65 , 10.45)	8.68	(7.79 , 9.61)	4.49	(3.98 , 5.04)	4.98	(4.43 , 5.58)
2008111	8.70	(7.62 , 9.78)	7.65	(6.75 , 8.59)	4.38	(3.79 , 5.01)	4.78	(4.19 , 5.41)
2008202	9.68	(8.89 , 10.47)	7.82	(6.98 , 8.71)	4.06	(3.62 , 4.52)	4.49	(3.96 , 5.05)
2008013	8.22	(7.32 , 9.12)	7.19	(6.38 , 8.04)	4.13	(3.64 , 4.65)	4.32	(3.80 , 4.87)
2008279	10.65	(9.57 , 11.73)	7.40	(6.52 , 8.33)	4.73	(4.12 , 5.38)	4.33	(3.77 , 4.92)
2008007	9.69	(8.71 , 10.66)	8.04	(7.18 , 8.94)	4.02	(3.50 , 4.57)	4.22	(3.71 , 4.77)

Family	α-acid 16 months		α-acid 28 months *		β-acid 16 months *		β-acid 28 months *	
2008090	10.23	(9.39 , 11.07)	8.44	(7.62 , 9.30)	4.20	(3.73 , 4.70)	4.45	(3.96 , 4.97)
2008055	8.85	(8.01 , 9.69)	7.24	(6.43 , 8.09)	4.98	(4.46 , 5.52)	4.70	(4.15 , 5.27)
2008271	9.19	(8.35 , 10.03)	7.69	(6.86 , 8.57)	3.88	(3.43 , 4.36)	4.20	(3.68 , 4.74)
2008035	8.71	(7.81 , 9.61)	7.73	(6.90 , 8.62)	4.17	(3.68 , 4.70)	4.55	(4.02 , 5.12)
2008212	8.29	(7.31 , 9.27)	7.95	(7.04 , 8.92)	5.04	(4.46 , 5.66)	4.87	(4.28 , 5.51)
2008149	11.00	(10.10 , 11.90)	7.44	(6.62 , 8.30)	5.07	(4.52 , 5.65)	4.50	(3.97 , 5.06)
2008170	8.74	(7.90 , 9.58)	7.84	(7.00 , 8.73)	3.99	(3.54 , 4.48)	4.11	(3.60 , 4.64)
2008247	8.61	(7.53 , 9.69)	8.16	(7.30 , 9.07)	3.86	(3.31 , 4.45)	4.35	(3.83 , 4.91)
2008110	9.17	(8.09 , 10.25)	7.48	(6.60 , 8.42)	4.33	(3.75 , 4.95)	4.63	(4.05 , 5.24)
2008043	7.10	(6.35 , 7.85)	6.85	(6.11 , 7.63)	4.05	(3.64 , 4.49)	3.99	(3.52 , 4.48)
2008188	8.68	(7.60 , 9.76)	7.36	(6.48 , 8.29)	4.73	(4.12 , 5.38)	4.72	(4.14 , 5.34)
2008080	8.33	(7.25 , 9.41)	7.35	(6.47 , 8.28)	4.53	(3.94 , 5.17)	4.30	(3.75 , 4.90)
2008239	8.25	(7.17 , 9.33)	7.64	(6.81 , 8.52)	4.62	(4.02 , 5.27)	5.06	(4.50 , 5.66)
2008075	10.53	(9.63 , 11.44)	7.43	(6.66 , 8.24)	4.82	(4.29 , 5.38)	4.71	(4.20 , 5.25)
2008114	8.74	(7.76 , 9.73)	6.94	(6.15 , 7.77)	4.42	(3.87 , 5.00)	4.83	(4.28 , 5.41)
2008166	8.57	(7.59 , 9.55)	7.02	(6.23 , 7.87)	3.84	(3.34 , 4.39)	3.98	(3.48 , 4.51)
2008252	9.11	(8.14 , 10.09)	7.44	(6.62 , 8.31)	4.18	(3.66 , 4.75)	4.50	(3.97 , 5.07)
2008150	9.65	(8.90 , 10.40)	7.56	(6.68 , 8.51)	3.73	(3.33 , 4.16)	4.44	(3.87 , 5.04)
2008009	10.31	(9.40 , 11.21)	8.18	(7.38 , 9.04)	3.84	(3.37 , 4.35)	4.06	(3.59 , 4.56)
2008081	11.79	(10.71 , 12.87)	8.81	(7.85 , 9.82)	5.08	(4.45 , 5.76)	4.96	(4.36 , 5.60)
2008041	10.41	(9.57 , 11.25)	8.08	(7.28 , 8.93)	4.22	(3.75 , 4.72)	4.31	(3.82 , 4.82)
2008061	8.23	(7.39 , 9.07)	7.60	(6.71 , 8.55)	4.04	(3.58 , 4.53)	4.42	(3.86 , 5.02)
2008087	9.36	(8.28 , 10.44)	7.95	(7.04 , 8.91)	4.20	(3.63 , 4.82)	4.85	(4.26 , 5.48)
2008147	10.76	(10.01 , 11.51)	8.63	(7.80 , 9.50)	4.14	(3.71 , 4.58)	4.85	(4.33 , 5.40)
2008258	11.04	(10.25 , 11.84)	7.92	(7.08 , 8.82)	6.40	(5.85 , 6.98)	6.09	(5.47 , 6.74)
2008130	9.30	(8.39 , 10.20)	8.40	(7.52 , 9.32)	3.93	(3.45 , 4.43)	4.40	(3.88 , 4.96)
2008100	10.00	(9.09 , 10.90)	8.08	(7.17 , 9.06)	4.43	(3.92 , 4.98)	5.15	(4.54 , 5.80)
2008132	10.25	(9.27 , 11.23)	7.27	(6.40 , 8.19)	4.74	(4.17 , 5.34)	4.58	(4.00 , 5.19)
2008256	9.77	(8.79 , 10.75)	6.89	(6.04 , 7.79)	6.16	(5.51 , 6.84)	5.65	(5.01 , 6.33)
2008093	7.92	(7.13 , 8.71)	6.96	(6.21 , 7.74)	3.44	(3.04 , 3.87)	4.13	(3.65 , 4.63)
2008108	8.14	(7.35 , 8.93)	6.94	(6.15 , 7.78)	4.60	(4.13 , 5.09)	4.53	(4.00 , 5.09)
2008179	8.88	(8.03 , 9.72)	7.84	(7.00 , 8.73)	4.75	(4.25 , 5.27)	4.79	(4.25 , 5.37)
2008192	9.10	(8.12 , 10.08)	7.06	(6.21 , 7.97)	5.81	(5.18 , 6.47)	4.59	(4.01 , 5.20)
2008089	10.09	(9.11 , 11.07)	7.74	(6.90 , 8.62)	4.43	(3.88 , 5.01)	4.41	(3.88 , 4.96)
2008064	9.40	(8.32 , 10.48)	7.66	(6.77 , 8.61)	3.96	(3.41 , 4.56)	4.44	(3.87 , 5.04)
2008269	8.55	(7.47 , 9.63)	7.61	(6.65 , 8.65)	4.38	(3.80 , 5.01)	4.54	(3.92 , 5.21)

Family	α-acid 16 months		α-acid 28 months *		β-acid 16 months *		β-acid 28 months *	
2008059	9.38	(8.29 , 10.46)	7.40	(6.58 , 8.27)	4.44	(3.85 , 5.07)	4.85	(4.30 , 5.43)
2008047	8.81	(7.82 , 9.79)	7.33	(6.51 , 8.19)	4.07	(3.54 , 4.63)	4.06	(3.56 , 4.60)
2008194	11.39	(10.41 , 12.36)	7.51	(6.68 , 8.38)	5.29	(4.69 , 5.92)	4.71	(4.16 , 5.28)
2008232	7.86	(6.88 , 8.83)	6.77	(5.99 , 7.60)	4.62	(4.06 , 5.21)	5.01	(4.45 , 5.60)
2008173	8.67	(7.58 , 9.75)	7.50	(6.68 , 8.38)	4.65	(4.04 , 5.30)	4.54	(4.00 , 5.10)
2008120	8.80	(7.82 , 9.78)	8.15	(7.29 , 9.06)	4.66	(4.09 , 5.26)	5.07	(4.50 , 5.66)
2008262	9.42	(8.44 , 10.40)	7.45	(6.57 , 8.39)	4.16	(3.63 , 4.72)	4.53	(3.96 , 5.14)
2008187	8.12	(7.14 , 9.10)	6.99	(6.19 , 7.83)	3.61	(3.12 , 4.14)	4.10	(3.59 , 4.63)
2008051	9.94	(8.96 , 10.92)	7.81	(6.91 , 8.77)	5.01	(4.43 , 5.63)	4.70	(4.12 , 5.32)
2008198	7.98	(7.00 , 8.95)	7.06	(6.26 , 7.90)	3.84	(3.33 , 4.38)	4.05	(3.55 , 4.59)
2008054	7.04	(6.25 , 7.84)	6.95	(6.21 , 7.74)	3.22	(2.83 , 3.63)	4.40	(3.91 , 4.92)
2008244	9.62	(8.77 , 10.46)	7.88	(7.03 , 8.77)	4.33	(3.86 , 4.84)	4.88	(4.33 , 5.47)
2008177	9.23	(8.15 , 10.32)	7.19	(6.38 , 8.04)	4.31	(3.73 , 4.94)	4.82	(4.27 , 5.40)
2008117	9.15	(8.25 , 10.06)	7.75	(6.96 , 8.57)	4.01	(3.53 , 4.53)	4.43	(3.94 , 4.96)
2008016	8.14	(7.35 , 8.94)	8.17	(7.31 , 9.08)	4.74	(4.27 , 5.24)	4.87	(4.32 , 5.46)
2008267	9.47	(8.49 , 10.45)	7.82	(6.98 , 8.71)	4.20	(3.67 , 4.77)	4.28	(3.76 , 4.82)
2008040	10.73	(9.65 , 11.81)	7.48	(6.60 , 8.42)	4.54	(3.94 , 5.18)	4.22	(3.67 , 4.81)
2008218	11.13	(10.29 , 11.97)	8.58	(7.69 , 9.51)	4.46	(3.98 , 4.97)	4.46	(3.93 , 5.02)
2008070	9.32	(8.34 , 10.30)	7.20	(6.34 , 8.12)	5.07	(4.48 , 5.69)	4.14	(3.60 , 4.73)
2008102	10.77	(9.69 , 11.85)	7.72	(6.88 , 8.60)	4.80	(4.19 , 5.46)	4.72	(4.18 , 5.29)
2008078	10.84	(9.76 , 11.92)	7.45	(6.57 , 8.39)	4.78	(4.17 , 5.43)	4.67	(4.09 , 5.29)
2008074	9.17	(8.33 , 10.01)	8.41	(7.59 , 9.27)	4.01	(3.55 , 4.49)	4.39	(3.89 , 4.91)

g.

Family	cohumulone (% of α-acid) 16 months *		cohumulone (% of α-acid) 28 months *		α-acid:β-acid 16 months *		α-acid:β-acid 28 months *		α-acid:total resin 16 months *		α-acid:total resin 28 months *	
2008003	0.31	(0.28 , 0.33)	0.33	(0.31 , 0.36)	2.29	(2.03 , 2.55)	1.78	(1.55 , 2.01)	0.68	(0.65 , 0.71)	0.62	(0.58 , 0.66)
2008190	0.28	(0.25 , 0.30)	0.29	(0.27 , 0.32)	2.04	(1.81 , 2.27)	1.61	(1.39 , 1.84)	0.66	(0.63 , 0.69)	0.60	(0.56 , 0.63)
2008209	0.30	(0.27 , 0.32)	0.32	(0.30 , 0.35)	2.43	(2.15 , 2.73)	1.80	(1.57 , 2.03)	0.70	(0.66 , 0.73)	0.63	(0.59 , 0.67)
2008151	0.29	(0.26 , 0.32)	0.29	(0.27 , 0.32)	2.16	(1.90 , 2.44)	1.73	(1.50 , 1.96)	0.67	(0.64 , 0.71)	0.62	(0.58 , 0.66)
2008039	0.33	(0.30 , 0.36)	0.31	(0.28 , 0.34)	2.47	(2.18 , 2.77)	1.83	(1.58 , 2.09)	0.70	(0.67 , 0.73)	0.63	(0.59 , 0.67)

Family	cohumulone (% of α-acid)		cohumulone (% of α-acid)		α-acid:β-acid		α-acid:β-acid		α-acid:total resin		α-acid:total resin	
	16 months *		28 months *		16 months *		28 months *		16 months *		28 months *	
2008152	0.27	(0.25 , 0.29)	0.30	(0.28 , 0.33)	2.11	(1.90 , 2.34)	1.54	(1.32 , 1.77)	0.67	(0.64 , 0.69)	0.59	(0.55 , 0.62)
2008001	0.30	(0.28 , 0.33)	0.30	(0.28 , 0.33)	2.29	(2.05 , 2.54)	1.97	(1.73 , 2.21)	0.69	(0.66 , 0.71)	0.65	(0.61 , 0.69)
2008073	0.30	(0.27 , 0.32)	0.32	(0.30 , 0.35)	1.95	(1.72 , 2.20)	1.74	(1.49 , 1.99)	0.65	(0.62 , 0.68)	0.62	(0.58 , 0.66)
2008168	0.28	(0.25 , 0.30)	0.31	(0.28 , 0.33)	1.95	(1.70 , 2.21)	1.59	(1.37 , 1.82)	0.64	(0.61 , 0.67)	0.58	(0.54 , 0.61)
2008161	0.27	(0.25 , 0.30)	0.29	(0.27 , 0.31)	2.32	(2.06 , 2.59)	1.63	(1.42 , 1.84)	0.69	(0.66 , 0.72)	0.59	(0.56 , 0.62)
2008290	0.27	(0.25 , 0.29)	0.30	(0.27 , 0.32)	2.45	(2.20 , 2.71)	1.93	(1.70 , 2.17)	0.70	(0.67 , 0.73)	0.64	(0.61 , 0.68)
2008208	0.28	(0.25 , 0.30)	0.28	(0.26 , 0.31)	2.34	(2.06 , 2.63)	1.71	(1.48 , 1.94)	0.69	(0.66 , 0.72)	0.61	(0.58 , 0.65)
2008251	0.28	(0.26 , 0.31)	0.29	(0.27 , 0.32)	1.97	(1.72 , 2.24)	1.63	(1.41 , 1.86)	0.65	(0.62 , 0.68)	0.61	(0.57 , 0.64)
2008052	0.29	(0.27 , 0.31)	0.33	(0.30 , 0.35)	2.32	(2.09 , 2.56)	1.95	(1.72 , 2.19)	0.69	(0.66 , 0.71)	0.65	(0.61 , 0.68)
2008037	0.26	(0.24 , 0.28)	0.28	(0.25 , 0.30)	2.13	(1.89 , 2.39)	1.85	(1.62 , 2.09)	0.67	(0.64 , 0.70)	0.64	(0.60 , 0.68)
2008154	0.33	(0.31 , 0.36)	0.37	(0.35 , 0.40)	2.24	(1.99 , 2.51)	1.89	(1.66 , 2.13)	0.68	(0.65 , 0.71)	0.64	(0.60 , 0.68)
2008136	0.28	(0.26 , 0.30)	0.31	(0.29 , 0.34)	2.21	(2.00 , 2.43)	1.77	(1.56 , 1.98)	0.68	(0.66 , 0.71)	0.63	(0.59 , 0.66)
2008096	0.31	(0.29 , 0.33)	0.32	(0.30 , 0.35)	1.99	(1.80 , 2.20)	1.83	(1.60 , 2.07)	0.66	(0.63 , 0.68)	0.63	(0.60 , 0.67)
2008071	0.29	(0.27 , 0.31)	0.31	(0.29 , 0.33)	2.31	(2.09 , 2.55)	1.81	(1.60 , 2.03)	0.69	(0.66 , 0.71)	0.63	(0.60 , 0.67)
2008124	0.29	(0.26 , 0.31)	0.32	(0.29 , 0.35)	2.14	(1.88 , 2.42)	1.80	(1.55 , 2.06)	0.67	(0.64 , 0.70)	0.63	(0.59 , 0.67)
2008145	0.30	(0.28 , 0.32)	0.31	(0.29 , 0.33)	2.15	(1.95 , 2.36)	1.83	(1.62 , 2.05)	0.67	(0.65 , 0.70)	0.64	(0.60 , 0.67)
2008084	0.27	(0.25 , 0.29)	0.31	(0.28 , 0.34)	1.99	(1.78 , 2.20)	1.69	(1.44 , 1.94)	0.65	(0.62 , 0.67)	0.61	(0.57 , 0.65)
2008083	0.28	(0.25 , 0.30)	0.31	(0.28 , 0.33)	2.18	(1.93 , 2.44)	2.08	(1.84 , 2.33)	0.68	(0.65 , 0.71)	0.66	(0.62 , 0.70)
2008056	0.29	(0.26 , 0.31)	0.31	(0.28 , 0.33)	1.89	(1.66 , 2.13)	1.59	(1.35 , 1.85)	0.64	(0.61 , 0.67)	0.59	(0.55 , 0.63)
2008261	0.31	(0.29 , 0.33)	0.32	(0.30 , 0.35)	2.19	(1.95 , 2.43)	1.82	(1.59 , 2.05)	0.68	(0.65 , 0.71)	0.63	(0.60 , 0.67)
2008157	0.29	(0.27 , 0.32)	0.32	(0.30 , 0.35)	2.18	(1.92 , 2.47)	1.72	(1.49 , 1.95)	0.67	(0.64 , 0.71)	0.62	(0.59 , 0.66)
2008006	0.27	(0.25 , 0.30)	0.33	(0.31 , 0.36)	1.93	(1.69 , 2.17)	1.96	(1.73 , 2.21)	0.65	(0.62 , 0.68)	0.64	(0.61 , 0.68)
2008240	0.32	(0.30 , 0.34)	0.32	(0.29 , 0.34)	1.93	(1.73 , 2.15)	1.98	(1.74 , 2.22)	0.64	(0.61 , 0.66)	0.65	(0.61 , 0.69)
2008103	0.26	(0.24 , 0.29)	0.30	(0.28 , 0.33)	2.17	(1.90 , 2.45)	1.73	(1.52 , 1.95)	0.67	(0.64 , 0.71)	0.62	(0.59 , 0.66)
2008148	0.31	(0.28 , 0.33)	0.33	(0.31 , 0.36)	2.13	(1.91 , 2.36)	1.80	(1.59 , 2.02)	0.66	(0.63 , 0.68)	0.61	(0.58 , 0.65)
2008236	0.36	(0.34 , 0.38)	0.36	(0.34 , 0.39)	2.20	(2.00 , 2.42)	1.70	(1.48 , 1.94)	0.68	(0.66 , 0.70)	0.61	(0.58 , 0.65)
2008259	0.29	(0.26 , 0.32)	0.31	(0.28 , 0.34)	2.16	(1.90 , 2.44)	1.86	(1.61 , 2.12)	0.67	(0.64 , 0.71)	0.64	(0.59 , 0.68)
2008191	0.38	(0.35 , 0.41)	0.39	(0.37 , 0.42)	2.09	(1.86 , 2.33)	1.69	(1.48 , 1.90)	0.67	(0.64 , 0.69)	0.62	(0.58 , 0.65)
2008242	0.31	(0.29 , 0.34)	0.30	(0.28 , 0.32)	1.69	(1.49 , 1.90)	1.51	(1.31 , 1.72)	0.61	(0.58 , 0.64)	0.58	(0.55 , 0.61)
2008248	0.26	(0.24 , 0.28)	0.27	(0.25 , 0.30)	1.88	(1.66 , 2.10)	1.76	(1.53 , 2.00)	0.63	(0.61 , 0.66)	0.61	(0.57 , 0.64)
2008010	0.28	(0.26 , 0.30)	0.34	(0.31 , 0.36)	2.43	(2.18 , 2.68)	1.87	(1.64 , 2.11)	0.70	(0.67 , 0.73)	0.64	(0.60 , 0.67)
2008263	0.28	(0.25 , 0.30)	0.32	(0.30 , 0.35)	2.11	(1.88 , 2.35)	1.76	(1.53 , 1.99)	0.67	(0.64 , 0.70)	0.63	(0.59 , 0.66)
2008153	0.28	(0.26 , 0.30)	0.31	(0.29 , 0.33)	2.34	(2.13 , 2.57)	1.79	(1.58 , 2.01)	0.69	(0.67 , 0.72)	0.63	(0.60 , 0.67)
2008119	0.30	(0.27 , 0.32)	0.32	(0.29 , 0.34)	2.29	(2.03 , 2.55)	1.79	(1.56 , 2.03)	0.69	(0.66 , 0.72)	0.63	(0.59 , 0.67)
2008206	0.34	(0.31 , 0.36)	0.33	(0.31 , 0.36)	2.22	(1.99 , 2.47)	1.79	(1.58 , 2.01)	0.68	(0.65 , 0.71)	0.63	(0.60 , 0.66)
2008139	0.28	(0.26 , 0.30)	0.30	(0.28 , 0.33)	1.53	(1.35 , 1.72)	1.53	(1.31 , 1.76)	0.57	(0.55 , 0.60)	0.58	(0.55 , 0.62)

Family	cohumulone (% of α-acid) 16 months *		cohumulone (% of α-acid) 28 months *		α-acid:β-acid 16 months *		α-acid:β-acid 28 months *		α-acid:total resin 16 months *		α-acid:total resin 28 months *	
2008106	0.29	(0.27 , 0.31)	0.32	(0.29 , 0.34)	2.26	(2.02 , 2.51)	1.59	(1.37 , 1.82)	0.69	(0.66 , 0.71)	0.59	(0.56 , 0.63)
2008122	0.31	(0.28 , 0.34)	0.32	(0.29 , 0.34)	1.85	(1.60 , 2.11)	1.76	(1.54 , 2.00)	0.63	(0.60 , 0.66)	0.61	(0.58 , 0.65)
2008155	0.36	(0.33 , 0.38)	0.33	(0.30 , 0.35)	2.20	(1.96 , 2.44)	1.74	(1.52 , 1.98)	0.68	(0.65 , 0.70)	0.62	(0.58 , 0.66)
2008088	0.29	(0.26 , 0.31)	0.32	(0.30 , 0.35)	2.13	(1.90 , 2.37)	1.81	(1.58 , 2.05)	0.67	(0.64 , 0.70)	0.63	(0.59 , 0.67)
2008111	0.27	(0.25 , 0.30)	0.30	(0.27 , 0.33)	2.02	(1.77 , 2.30)	1.66	(1.42 , 1.92)	0.66	(0.63 , 0.69)	0.61	(0.57 , 0.65)
2008202	0.33	(0.31 , 0.35)	0.35	(0.32 , 0.37)	2.38	(2.17 , 2.61)	1.82	(1.59 , 2.05)	0.70	(0.67 , 0.72)	0.63	(0.59 , 0.67)
2008013	0.25	(0.23 , 0.28)	0.29	(0.26 , 0.31)	2.11	(1.89 , 2.35)	1.75	(1.52 , 1.99)	0.66	(0.63 , 0.69)	0.61	(0.57 , 0.65)
2008279	0.31	(0.29 , 0.34)	0.34	(0.31 , 0.37)	2.20	(1.93 , 2.48)	1.79	(1.54 , 2.05)	0.68	(0.65 , 0.71)	0.63	(0.59 , 0.67)
2008007	0.31	(0.29 , 0.34)	0.33	(0.30 , 0.35)	2.46	(2.20 , 2.74)	2.10	(1.86 , 2.35)	0.70	(0.67 , 0.73)	0.66	(0.62 , 0.70)
2008090	0.32	(0.30 , 0.34)	0.33	(0.30 , 0.35)	2.39	(2.16 , 2.63)	2.02	(1.80 , 2.24)	0.70	(0.67 , 0.72)	0.66	(0.62 , 0.69)
2008055	0.27	(0.25 , 0.29)	0.30	(0.28 , 0.33)	1.82	(1.63 , 2.03)	1.59	(1.37 , 1.82)	0.63	(0.60 , 0.65)	0.59	(0.55 , 0.62)
2008271	0.30	(0.28 , 0.32)	0.33	(0.30 , 0.35)	2.41	(2.18 , 2.65)	1.98	(1.75 , 2.23)	0.69	(0.67 , 0.72)	0.65	(0.61 , 0.69)
2008035	0.28	(0.26 , 0.30)	0.31	(0.28 , 0.33)	2.07	(1.85 , 2.31)	1.78	(1.55 , 2.02)	0.66	(0.63 , 0.69)	0.63	(0.59 , 0.66)
2008212	0.29	(0.27 , 0.32)	0.32	(0.29 , 0.35)	1.73	(1.51 , 1.97)	1.70	(1.46 , 1.96)	0.61	(0.58 , 0.64)	0.61	(0.57 , 0.65)
2008149	0.36	(0.33 , 0.38)	0.33	(0.31 , 0.36)	2.15	(1.92 , 2.39)	1.71	(1.48 , 1.94)	0.67	(0.65 , 0.70)	0.61	(0.58 , 0.65)
2008170	0.27	(0.25 , 0.29)	0.31	(0.28 , 0.33)	2.29	(2.06 , 2.52)	2.09	(1.85 , 2.33)	0.68	(0.66 , 0.71)	0.66	(0.62 , 0.70)
2008247	0.27	(0.25 , 0.30)	0.34	(0.31 , 0.37)	2.28	(2.01 , 2.57)	2.03	(1.79 , 2.27)	0.69	(0.65 , 0.72)	0.65	(0.62 , 0.69)
2008110	0.31	(0.28 , 0.34)	0.33	(0.30 , 0.36)	2.18	(1.91 , 2.46)	1.68	(1.44 , 1.94)	0.67	(0.64 , 0.71)	0.61	(0.57 , 0.65)
2008043	0.27	(0.25 , 0.29)	0.32	(0.30 , 0.34)	1.84	(1.66 , 2.04)	1.79	(1.58 , 2.01)	0.63	(0.61 , 0.66)	0.63	(0.60 , 0.67)
2008188	0.30	(0.27 , 0.33)	0.35	(0.32 , 0.38)	1.89	(1.65 , 2.16)	1.62	(1.38 , 1.88)	0.64	(0.61 , 0.67)	0.60	(0.56 , 0.64)
2008080	0.27	(0.25 , 0.30)	0.31	(0.28 , 0.34)	1.96	(1.71 , 2.23)	1.80	(1.55 , 2.06)	0.64	(0.61 , 0.67)	0.63	(0.59 , 0.67)
2008239	0.31	(0.29 , 0.34)	0.34	(0.31 , 0.36)	1.86	(1.62 , 2.13)	1.56	(1.34 , 1.78)	0.63	(0.60 , 0.66)	0.58	(0.55 , 0.62)
2008075	0.27	(0.25 , 0.29)	0.30	(0.28 , 0.33)	2.15	(1.92 , 2.39)	1.62	(1.42 , 1.84)	0.68	(0.65 , 0.70)	0.60	(0.57 , 0.64)
2008114	0.25	(0.23 , 0.27)	0.28	(0.26 , 0.31)	2.08	(1.84 , 2.33)	1.47	(1.25 , 1.69)	0.66	(0.63 , 0.69)	0.57	(0.53 , 0.60)
2008166	0.36	(0.33 , 0.39)	0.33	(0.31 , 0.36)	2.33	(2.07 , 2.60)	1.93	(1.70 , 2.17)	0.69	(0.66 , 0.72)	0.64	(0.60 , 0.68)
2008252	0.28	(0.26 , 0.30)	0.28	(0.26 , 0.31)	2.27	(2.02 , 2.53)	1.75	(1.52 , 1.99)	0.67	(0.64 , 0.70)	0.61	(0.58 , 0.65)
2008150	0.31	(0.29 , 0.33)	0.33	(0.30 , 0.36)	2.59	(2.38 , 2.82)	1.79	(1.54 , 2.05)	0.72	(0.69 , 0.74)	0.63	(0.59 , 0.67)
2008009	0.26	(0.24 , 0.28)	0.28	(0.26 , 0.30)	2.76	(2.50 , 3.03)	2.26	(2.03 , 2.49)	0.72	(0.69 , 0.75)	0.67	(0.64 , 0.71)
2008081	0.28	(0.25 , 0.31)	0.30	(0.27 , 0.33)	2.26	(1.99 , 2.54)	1.88	(1.62 , 2.14)	0.68	(0.65 , 0.71)	0.64	(0.60 , 0.68)
2008041	0.34	(0.31 , 0.36)	0.29	(0.26 , 0.31)	2.48	(2.24 , 2.72)	2.01	(1.79 , 2.23)	0.70	(0.68 , 0.73)	0.66	(0.62 , 0.69)
2008061	0.28	(0.26 , 0.30)	0.31	(0.28 , 0.34)	2.10	(1.89 , 2.33)	1.82	(1.57 , 2.08)	0.67	(0.64 , 0.70)	0.63	(0.59 , 0.67)
2008087	0.30	(0.27 , 0.33)	0.34	(0.32 , 0.37)	2.24	(1.97 , 2.53)	1.71	(1.46 , 1.96)	0.68	(0.65 , 0.72)	0.61	(0.57 , 0.66)
2008147	0.31	(0.29 , 0.33)	0.32	(0.29 , 0.34)	2.61	(2.39 , 2.84)	1.87	(1.66 , 2.09)	0.71	(0.69 , 0.74)	0.64	(0.60 , 0.67)
2008258	0.26	(0.24 , 0.28)	0.30	(0.28 , 0.32)	1.74	(1.56 , 1.94)	1.41	(1.20 , 1.64)	0.61	(0.59 , 0.64)	0.55	(0.51 , 0.58)
2008130	0.29	(0.26 , 0.31)	0.32	(0.30 , 0.35)	2.39	(2.15 , 2.65)	2.10	(1.86 , 2.34)	0.70	(0.67 , 0.72)	0.66	(0.62 , 0.70)
2008100	0.27	(0.24 , 0.29)	0.31	(0.28 , 0.34)	2.26	(2.03 , 2.51)	1.65	(1.40 , 1.91)	0.68	(0.66 , 0.71)	0.60	(0.57 , 0.65)

Family	cohumulone (% of α-acid) 16 months *		cohumulone (% of α-acid) 28 months *		α-acid:β-acid 16 months *		α-acid:β-acid 28 months *		α-acid:total resin 16 months *		α-acid:total resin 28 months *	
2008132	0.30	(0.27 , 0.32)	0.31	(0.28 , 0.34)	2.13	(1.89 , 2.39)	1.63	(1.39 , 1.89)	0.67	(0.64 , 0.70)	0.60	(0.56 , 0.64)
2008256	0.30	(0.28 , 0.33)	0.32	(0.29 , 0.35)	1.67	(1.45 , 1.90)	1.37	(1.14 , 1.62)	0.60	(0.57 , 0.63)	0.51	(0.47 , 0.54)
2008093	0.30	(0.28 , 0.32)	0.32	(0.30 , 0.34)	2.40	(2.18 , 2.62)	1.80	(1.59 , 2.02)	0.70	(0.67 , 0.72)	0.63	(0.59 , 0.66)
2008108	0.29	(0.27 , 0.31)	0.31	(0.29 , 0.33)	1.83	(1.64 , 2.02)	1.59	(1.37 , 1.82)	0.61	(0.59 , 0.64)	0.59	(0.55 , 0.62)
2008179	0.24	(0.22 , 0.26)	0.28	(0.26 , 0.30)	1.95	(1.74 , 2.17)	1.72	(1.49 , 1.95)	0.65	(0.62 , 0.67)	0.61	(0.58 , 0.65)
2008192	0.34	(0.31 , 0.37)	0.32	(0.29 , 0.34)	1.67	(1.46 , 1.90)	1.58	(1.34 , 1.83)	0.60	(0.57 , 0.63)	0.58	(0.54 , 0.62)
2008089	0.29	(0.27 , 0.31)	0.32	(0.29 , 0.34)	2.25	(2.00 , 2.52)	1.81	(1.58 , 2.04)	0.68	(0.66 , 0.71)	0.63	(0.60 , 0.67)
2008064	0.28	(0.26 , 0.31)	0.30	(0.27 , 0.33)	2.42	(2.14 , 2.72)	1.81	(1.56 , 2.07)	0.70	(0.66 , 0.73)	0.63	(0.59 , 0.67)
2008269	0.34	(0.31 , 0.36)	0.31	(0.28 , 0.35)	1.94	(1.69 , 2.21)	1.77	(1.49 , 2.06)	0.64	(0.61 , 0.67)	0.62	(0.57 , 0.67)
2008059	0.30	(0.27 , 0.32)	0.33	(0.30 , 0.35)	2.14	(1.88 , 2.42)	1.57	(1.35 , 1.80)	0.67	(0.64 , 0.70)	0.59	(0.55 , 0.63)
2008047	0.32	(0.30 , 0.35)	0.35	(0.33 , 0.38)	2.21	(1.96 , 2.48)	1.92	(1.69 , 2.16)	0.68	(0.65 , 0.71)	0.64	(0.60 , 0.68)
2008194	0.29	(0.27 , 0.32)	0.30	(0.27 , 0.32)	2.05	(1.81 , 2.31)	1.64	(1.42 , 1.87)	0.66	(0.63 , 0.69)	0.61	(0.57 , 0.64)
2008232	0.27	(0.25 , 0.29)	0.30	(0.27 , 0.32)	1.79	(1.57 , 2.03)	1.38	(1.17 , 1.60)	0.62	(0.59 , 0.65)	0.54	(0.51 , 0.58)
2008173	0.26	(0.23 , 0.28)	0.31	(0.29 , 0.34)	1.91	(1.66 , 2.18)	1.73	(1.50 , 1.96)	0.64	(0.61 , 0.68)	0.62	(0.58 , 0.65)
2008120	0.26	(0.24 , 0.29)	0.30	(0.27 , 0.32)	1.96	(1.72 , 2.21)	1.67	(1.45 , 1.91)	0.65	(0.62 , 0.68)	0.61	(0.57 , 0.65)
2008262	0.29	(0.27 , 0.32)	0.31	(0.28 , 0.33)	2.28	(2.03 , 2.55)	1.71	(1.46 , 1.97)	0.69	(0.66 , 0.72)	0.61	(0.57 , 0.65)
2008187	0.28	(0.26 , 0.30)	0.30	(0.27 , 0.32)	2.23	(1.98 , 2.50)	1.78	(1.55 , 2.01)	0.68	(0.65 , 0.71)	0.63	(0.59 , 0.66)
2008051	0.32	(0.29 , 0.34)	0.33	(0.30 , 0.36)	1.98	(1.74 , 2.23)	1.74	(1.49 , 2.00)	0.65	(0.62 , 0.68)	0.62	(0.58 , 0.66)
2008198	0.30	(0.28 , 0.33)	0.32	(0.29 , 0.34)	2.13	(1.89 , 2.39)	1.85	(1.62 , 2.09)	0.67	(0.64 , 0.70)	0.64	(0.60 , 0.67)
2008054	0.28	(0.26 , 0.30)	0.31	(0.29 , 0.34)	2.22	(2.01 , 2.44)	1.62	(1.41 , 1.83)	0.68	(0.66 , 0.71)	0.59	(0.56 , 0.62)
2008244	0.28	(0.26 , 0.30)	0.31	(0.29 , 0.34)	2.27	(2.05 , 2.50)	1.71	(1.49 , 1.95)	0.68	(0.66 , 0.71)	0.61	(0.57 , 0.65)
2008177	0.29	(0.26 , 0.32)	0.29	(0.26 , 0.31)	2.16	(1.90 , 2.44)	1.53	(1.31 , 1.75)	0.67	(0.64 , 0.71)	0.58	(0.55 , 0.62)
2008117	0.34	(0.31 , 0.36)	0.36	(0.34 , 0.39)	2.28	(2.04 , 2.53)	1.79	(1.58 , 2.01)	0.69	(0.66 , 0.71)	0.63	(0.60 , 0.67)
2008016	0.27	(0.25 , 0.29)	0.31	(0.29 , 0.34)	1.75	(1.57 , 1.95)	1.74	(1.52 , 1.98)	0.62	(0.60 , 0.64)	0.62	(0.58 , 0.66)
2008267	0.26	(0.24 , 0.28)	0.32	(0.30 , 0.35)	2.22	(1.97 , 2.49)	1.92	(1.69 , 2.16)	0.68	(0.65 , 0.71)	0.64	(0.61 , 0.68)
2008040	0.30	(0.27 , 0.33)	0.33	(0.30 , 0.36)	2.35	(2.08 , 2.65)	1.92	(1.67 , 2.19)	0.69	(0.66 , 0.72)	0.64	(0.60 , 0.68)
2008218	0.27	(0.25 , 0.29)	0.31	(0.29 , 0.33)	2.42	(2.19 , 2.66)	2.07	(1.83 , 2.31)	0.70	(0.67 , 0.73)	0.66	(0.62 , 0.70)
2008070	0.28	(0.26 , 0.31)	0.24	(0.21 , 0.26)	1.93	(1.70 , 2.18)	1.87	(1.61 , 2.13)	0.64	(0.61 , 0.67)	0.64	(0.59 , 0.68)
2008102	0.30	(0.28 , 0.33)	0.32	(0.30 , 0.35)	2.19	(1.93 , 2.48)	1.72	(1.49 , 1.95)	0.68	(0.65 , 0.71)	0.62	(0.58 , 0.66)
2008078	0.28	(0.25 , 0.31)	0.34	(0.31 , 0.37)	2.20	(1.93 , 2.48)	1.66	(1.41 , 1.91)	0.68	(0.64 , 0.71)	0.61	(0.57 , 0.65)
2008074	0.28	(0.26 , 0.30)	0.30	(0.28 , 0.32)	2.30	(2.08 , 2.54)	2.07	(1.85 , 2.30)	0.69	(0.66 , 0.72)	0.66	(0.63 , 0.70)
