# Supplementary material for: Quantitative genetic parameters for yield, plant growth and cone chemical traits in hop (Humulus lupulus L.)
Source: BMC Genet. 2014 Feb 13;15:22. doi: 10.1186/1471-2156-15-22 (PMC3927220; doi:10.1186/1471-2156-15-22)
Supplement: Additional file 2 — Comparisons between quantitative genetic parameters calculated for hop in this study and previous studies. Estimates of quantitative genetic parameters of hop cone chemical traits and yield calculated in this study are compared to the results of previous studies. a. refers to the number of families and estimates of narrow-sense heritability calculated for cone chemical traits and yield. The values reported from this study are averages of data from two seasons for cone chemical traits and one season for yield. b. refers to additive genetic correlations between the traits α-acid, β-acid and cone yield from previous studies of hop, compared to the values determined in this study. Correlations statistically different to zero (P < 0.05) are shown in bold. [file 1471-2156-15-22-S2.pdf]

Additional file 2 Comparisons between quantitative genetic parameters calculated for hop in this study and previous studies.

Estimates of quantitative genetic parameters of hop cone chemical traits and yield calculated in this study are compared to the results of previous studies. **a.** refers to the number of families and estimates of narrow-sense heritability calculated for cone chemical traits and yield. The values reported from this study are averages of data from two seasons for cone chemical traits and one season for yield. **b.** refers to additive genetic correlations between the traits α-acid, β-acid and cone yield from previous studies of hop, compared to the values determined in this study. Correlations statistically different to zero ($P < 0.05$) are shown in bold.

a.

**Study**	***n* families**	**cohumulone**	**colupulone**	**α-acid**	**β-acid**	**α-acid:β-acid**	**yield**
This study	107-108	0.23	0.18	0.22	0.18	0.21	0.00
Henning et al. (2005)	25	0.87	0.89	0.76	0.57	-	0.71
Murakami (1999)	12	-	-	0.50	0.75	0.00	-
Henning et al. (1997a)	14	-	-	0.88	0.35	-	0.20

b.

**Study**	**α-acid x β-acid**	**α-acid x yield**	**β-acid x yield**
This study – season 1	**0.48**	**-**	**-**
This study – season 2	-0.08	**-0.93**	**-0.63**
Henning et al. (2005)	**0.71**	0.28	**0.73**
Murakami (1999)	-	-	-
Henning et al. (1997a)	**-0.42**	**-0.66**	**0.84**
Henning et al. (1997b)	**-0.74**	-0.12	0.04
