# Supplementary material for: Quantitative genetic parameters for yield, plant growth and cone chemical traits in hop (Humulus lupulus L.)
Source: BMC Genet. 2014 Feb 13;15:22. doi: 10.1186/1471-2156-15-22 (PMC3927220; doi:10.1186/1471-2156-15-22)
Supplement: Additional file 3 — The female parents of a progeny trial used to investigate quantitative genetic variation in hop. 'Hop accession’ refers to the cultivar name or accession number of the hop accessions used as a female parent. 'Origin’ refers to the country where the hop accessions were produced. [file 1471-2156-15-22-S3.pdf]

Additional file 3 The female parents of a progeny trial used to investigate quantitative genetic variation in hop.
'Hop accession' refers to the cultivar name or accession number of the hop accessions used as a female parent. 'Origin' refers to the country where the hop accessions were produced.

Hop accession	Origin
21055	USA
21194	USA
00-999	Australia
15-72-1	unknown
17/54/12	UK
21055 (2n)	USA
27/70/4	UK
30-74-32	Australia
71-75-1	Australia
8428-036	Australia
8451-077	Australia
89-012-017	Australia
89-012-024	Australia
89-012-052	Australia
89-022-020	Australia
89-022-033	Australia
89-103-005	Australia
89-123-001	Australia
89-124-018	Australia
89-134-022	Australia
89-135-011	Australia
89-135-016	Australia
90-014-095	Australia
91-004-013	Australia
91-008-046	Australia
91-021-020	Australia
91-031-021	Australia
94-138-006 (2n)	Australia
97-113-003	Australia
AF 3/26	South Africa
AHIL	Slovenia
AQUILA	USA
AURORA	Slovenia
B23	Australia
B24/23	Australia
BANNER	USA
BH 2/72	South Africa
BOBEK	Slovenia
BREWERS GOLD	UK
BRUNY ISLAND	Australia
BUKET	Slovenia
BULLION	UK
C10	Australia
CHINOOK	USA
CLUSTER	USA
COLUMBUS	USA
D6	Australia
E2	USA
E-85-16	Australia
E-85-20	Australia
EASTERN GOLD	Japan
ED-85-36	Australia
EG-85-17	Australia
EI-85-33	Australia

Hop accession	Origin
ELSASSER	France
EN-85-32	Australia
EP-86-4	Australia
EROICA	USA
EX FRF	Australia
F-84-19	Australia
FK21	Japan
G-85-9	Australia
GALENA	USA
GOLDING	UK
HALLERTAU	Germany
HERSBRUCKER	Germany
HERSBRUCKER G	Germany
JAPANESE ROGUE	Japan
K56	USA
K-85-9	Australia
L1	USA
L1 EX FRF	USA
LA-85-70	Australia
LB-85-2	Australia
ME-85-33	Australia
NORTHERN BREWER	UK
NUGGET	USA
OM26	UK
PERLE	Germany
S-5524	Australia
SAAZ 36	Czech Republic
SHINSHUWASE	Japan
SMOOTHCONE	New Zealand
SOUTHERN BREWER	South Africa
STYRIAN	Australia
SWISS TETTNANG	Germany
TILLICUM	USA
U-85-10	Australia
UNKNOWN 09	Australia
V-85-9	Australia
VOJVODINA	Slovenia
W-86-26	Australia
WYE CHALLENGER	UK
WYE NORTHDOWN	UK
WYE TARGET	UK
YEOMAN	UK
ZENITH	UK

Each accession was used as a female parent once, except for accessions 21194, 21055(2n), NUGGET, CLUSTER and SWISS TETTNANG which were used twice; and individuals 21055 and 94-138-006 (2n) which were used four times.
